# Genome wide analysis of the complete GlnR nitrogen-response regulon in *Mycobacterium smegmatis*

**DOI:** 10.1186/1471-2164-14-301

**Published:** 2013-05-04

**Authors:** Victoria A Jenkins, Geraint R Barton, Brian D Robertson, Kerstin J Williams

**Affiliations:** 1 MRC Centre for Molecular Bacteriology and Infection, Imperial College London, South Kensington, London SW7 2AZ, UK; 2 Centre for Integrative Systems Biology and Bioinformatics, Imperial College London, South Kensington, London SW7 2AZ, UK

## Abstract

**Background:**

Nitrogen is an essential element for bacterial growth and an important component of biological macromolecules. Consequently, responding to nitrogen limitation is critical for bacterial survival and involves the interplay of signalling pathways and transcriptional regulation of nitrogen assimilation and scavenging genes. In the soil dwelling saprophyte *Mycobacterium smegmatis* the OmpR-type response regulator GlnR is thought to mediate the transcriptomic response to nitrogen limitation. However, to date only ten genes have been shown to be in the GlnR regulon, a vastly reduced number compared to other organisms.

**Results:**

We investigated the role of GlnR in the nitrogen limitation response and determined the entire GlnR regulon*,* by combining expression profiling of *M. smegmatis* wild type and *glnR* deletion mutant, with GlnR-specific chromatin immunoprecipitation and high throughput sequencing. We identify 53 GlnR binding sites during nitrogen limitation that control the expression of over 100 genes, demonstrating that GlnR is the regulator controlling the assimilation and utilisation of nitrogen. We also determine a consensus GlnR binding motif and identify key residues within the motif that are required for specific GlnR binding.

**Conclusions:**

We have demonstrated that GlnR is the global nitrogen response regulator in *M. smegmatis,* directly regulating the expression of more than 100 genes. GlnR controls key nitrogen stress survival processes including primary nitrogen metabolism pathways, the ability to utilise nitrate and urea as alternative nitrogen sources, and the potential to use cellular components to provide a source of ammonium. These studies further our understanding of how mycobacteria survive nutrient limiting conditions.

## Background

Mycobacteria belong to the GC-rich Actinomycetes, and as a genus contain diverse species including human pathogens, such as *Mycobacterium tuberculosis* and *Mycobacterium leprae,* as well as free-living non-pathogenic soil bacteria such as *Mycobacterium smegmatis*[1]. Mycobacteria must compete for nutrients and adapt to changing environments in order to survive, and nitrogen is essential for the synthesis of cellular macromolecules such as amino acids, nucleotides and cell wall components [2]. Consequently, bacteria have developed complex systems that allow them to sense internal and external nitrogen levels and adjust their metabolism accordingly. The metabolic response to nitrogen limitation has been extensively studied in *E. coli*[3-6] which is often proposed as the prokaryotic model for this. However the regulation of nitrogen metabolism has also been studied in other Actinomycetes, such as *Corynebacterium glutamicum* and *Streptomyces* species, and the signals, regulation and response mechanisms are different in these organisms, both compared to *E. coli* and to each other [7-13]. Therefore, it is likely that mycobacteria also possess unique responses to nitrogen limitation, but this has not been studied in detail.

*M. smegmatis*, presumably as a consequence of living in the soil, contains the largest number of genes predicted to be involved in nitrogen metabolism within the genus [14]. Notably it contains three ammonium transporters (Amt1, AmtA and AmtB) in the cell wall, more than any other mycobacterial species, suggesting ammonium is an important nitrogen source for this organism [14]. Once ammonium has entered the cell via diffusion across the cytoplasmic membrane or by protein-dependent transport, it is assimilated into the major biosynthetic nitrogen donors L-glutamate and L-glutamine through one of two pathways, depending on nitrogen availability. The low ammonium affinity glutamate dehydrogenase (GDH) enzyme is favourable in situations of nitrogen excess, whereas during nitrogen limitation the energy-requiring, higher affinity glutamine synthetase/glutamate synthase (glutamine:2-oxoglutarate aminotransferase) (GS/GOGAT) enzymes are required to meet the metabolic needs of the cell (for mycobacterial nitrogen metabolism reviews see [15,16]). Not only does nitrogen limitation lead to the switching of biosynthetic pathways, it also induces the expression of several key mycobacterial nitrogen metabolism genes, including the *amtB* operon encoding the AmtB ammonium transporter, a GlnK (PII) signalling protein and an adenylyl transferase (GlnD), the two other ammonium transporters *amt1* and *amtA*, glutamine synthetase (*glnA1*) and glutamate synthase (*gltBD*) [17-19]. Post translational modifications of key nitrogen control enzymes also occurs in response to nitrogen limitation. GlnD adenylylates the GlnK (PII) signalling protein on a conserved tyrosine residue in response to nitrogen limitation [20] which causes the PII protein to dissociate from AmtB porin channel, where it is bound [21,22], permitting increased ammonium influx [23]. The GS enzyme is also post-translationally modified during nitrogen limitation, undergoing de-adenylylation by GlnE. The de-adenylylated GS enzyme is fully active [24] ensuring maximal glutamine and glutamate synthesis occurs during times of nitrogen austerity. However, there are still many important gaps in our knowledge of nitrogen metabolism and its regulation in mycobacteria. For instance, the signal of nitrogen cellular status is unknown. Recent studies in our laboratory have shown that the intracellular ratio of 2-oxoglutarate:glutamine in *M. smegmatis* greatly increases during nitrogen limitation and decreases when nitrogen is replenished, suggesting this may be the intracellular signal in mycobacteria [25]. However, how this signal is detected and transmitted into transcriptional and post-translational responses is unknown. The role of the PII proteins in mycobacterial nitrogen control is also unclear. In *E. coli* PII-UMP controls the activity of the NtrC response regulator [26], yet in mycobacteria PII-AMP does not mediate the transcriptional response to nitrogen limitation [20]. Finally, the regulator(s) responsible for the transcriptional response to nitrogen limitation in *M. smegmatis* and the genes that make up this response are currently unknown.

In enteric bacteria, the transcriptional response to nitrogen limitation is mediated by the NtrBC two-component system [4,6], which activates the expression of over 100 genes [4,6]. In *C. glutamicum*, the TetR-type response regulator AmtR controls the transcriptional of at least 33 genes [9,17], and in *Streptomyces,* the OmpR-type response regulator GlnR control nitrogen metabolism [27], at least 50 nitrogen response genes in *S. coelicolor* and at least 44 genes in *S. venezuelae*[28-30]. *M. smegmatis* does not contain an NtrBC homolog, but does contain homologs of both *S. coelicolor* GlnR (MSMEG5784; 60% identity) and *C. glutamicum* AmtR (MSMEG4300; 42% identity) [14]. To date no role has been reported for AmtR in mycobacteria, and no *C. glutamicum* AmtR binding site motifs have been identified in any mycobacterial genome [14]. However, the *S. coelicolor* GlnR binding site motif has been identified in mycobacteria with three highly conserved *cis* elements located upstream of *M. smegmatis amtB-glnK-glnD* operon, *amt1* and *glnA1* genes [14]. Experimental confirmation of these binding sites led to the assignment of these five genes to the *M. smegmatis* GlnR regulon [31]. We have recently shown that *M. smegmatis* GlnR also regulates the expression of *amtA*, *nirB/D* (nitrite reductase) and *gltB/D* in response to nitrogen stress [32]. However, given the number of nitrogen metabolism-related genes in the *M. smegmatis* genome, it is likely that many more are GlnR-regulated, or that there are additional nitrogen response regulators.

Therefore the aim of this study was to apply a global approach to the *in vivo* identification of GlnR regulated genes in *M. smegmatis*. We combined genome-wide expression profiling, comparing a *glnR* mutant to the wild-type strain during nitrogen limited growth, with global analysis of GlnR-DNA interactions by Chromatin Immunoprecipitation (ChIP) and high-throughput sequencing (ChIP-seq). We show that GlnR is the global nitrogen regulator in mycobacteria and plays a key role in regulating the assimilation and utilisation of nitrogen, controlling the expression of over 100 genes. We demonstrate that GlnR can control the expression of divergent genes, and that it functions as both an activator and repressor of transcription. We also identify the consensus DNA-binding motif found in all the GlnR binding sites and determine key nucleotides in the motif for specific GlnR binding.

## Results

### GlnR is the global regulator of gene expression in response to nitrogen limitation

We studied the expression profiles of *M. smegmatis* wild type and Δ*glnR* deletion mutant [32] grown in nitrogen limiting conditions, in order to identify the genes under GlnR control. *M. smegmatis* wild type and Δ*glnR* mutant were harvested one hour after nitrogen run-out, total RNA was extracted and cDNA hybridised to the *M. smegmatis* microarray. Data was normalised and genes were considered significantly differentially expressed when they showed greater than 2-fold difference in expression between the wild type and Δ*glnR* mutant with an FDR corrected p-value of <0.01. Fully annotated microarray data have been deposited in BμG@Sbase (accession number E-BUGS-143; http://bugs.sgul.ac.uk/E-BUGS-143) and also ArrayExpress (accession number E-BUGS-143). The 10 previously identified GlnR-regulated genes were all confirmed to be under GlnR control during nitrogen stress (i.e. differential expression in the wild type compared to the Δ*glnR* mutant), but in addition a total of 392 genes were significantly up-regulated and 291 significantly down regulated (Additional file 1: Table S1). This indicates that GlnR mediates (directly or indirectly) the expression of over 680 genes.

### Identification of GlnR binding sites across the genome during nitrogen limitation

In order to identify which of the genes identified by transcriptional profiling are directly regulated by GlnR we used ChIP-seq to identify the location of GlnR binding sites in the genome during nitrogen limitation. Cells were grown in 1 mM (limiting) or 30 mM (excess) ammonium sulphate, and DNA-protein complexes were cross-linked one hour after ammonium depletion; nitrogen excess samples were cross-linked at the same time point, cells were then lysed and the DNA sheared by sonication. GlnR-bound DNA fragments were immunoprecipitated using affinity-purified anti-GlnR polyclonal antibody. We performed quantitative PCR on the glutamine synthetase (*glnA1*) and nitrite reductase (*nirB*) promoter regions to confirm the enrichment of GlnR binding regions in nitrogen limited cells compared to nitrogen replete; a gene thought not to be GlnR regulated (MSMEG3224) was included as a negative control (Additional file 2: Figure S1).

Immunoprecipitated DNA was then prepared for sequencing using the Illumina ChIP-seq library kit, and DNA libraries sequenced using an Illumina HiSeq2000, which generated approximately 160 million reads per sample that were then mapped to the *M. smegmatis* genome using Bowtie [33]. All ChIP-seq data files have been deposited into ArrayExpress (accession number E-MTAB-1456). GlnR binding regions were identified using the peak-calling algorithm SISSRs (Site Identification for Short Sequence Reads) [34], with peaks defined as significant if they showed greater than 5-fold enrichment in the immunoprecipitated sample compared to the input control at a p value of < 0.005. This identified 53 GlnR binding sites during nitrogen limitation (Table 1), 5 of which were also observed in nitrogen excess conditions (Table 2), but with much lower peak intensity values. For example a GlnR binding site was identified under both conditions upstream of *glnA* (MSMEG4290) with a peak intensity value of 6.3 in nitrogen excess and 184.7 in nitrogen limitation (Table 2). All GlnR binding sites were located in the promoter regions of genes, except peak number 52, which was located within MSMEG6817.

**Table 1:** **GlnR binding regions identified by ChIP-seq and corresponding gene expression fold change (wild type vs *glnR *deletion strain) in *M. smegmatis *during nitrogen limitation**

**Peak no.**^**a**^	**Coordinates**^**b**^	**Peak intensity**^**c**^	**Adjacent gene (s)**^**d**^	**Fold change in gene expression**^**e**^	**Gene annotation**
1	501431-501471	8.4	MSMEG0427*	76.4	*nirB* Nitrite reductase, large subunit
2	508651-508691	42.9	MSMEG0432*	18.3	*nnaR* Transcriptional regulator
3	510091-510131	8.4	MSMEG0433	24.6	*narK3* Nitrate extrusion protein
4	647871-647911	27.1	MSMEG0572*	263.4	Putative uncharacterised protein
5	864391-864431	6.1	MSMEG0780L*	23.0	Phosphotransferase enzyme family protein
			MSMEG0781R	8.4	Amino acid permease
6	1121631-1121671	54.3	MSMEG1052	6.3	Amino acid carrier protein
7	1142851-1142891	6.5	MSMEG1078L	-3.8	Hydrolase
			MSMEG1079R*	3.4	Putative uncharacterised protein
8	1146711-1146751	71.9	MSMEG1082	277.4	Putative response regulator, LuxR family
9	1238491-1238531	19.3	MSMEG1177L	10.7	Cytosine/purines/uracil/thiamine/allantoin permease
			MSMEG1178R	3.5	Transcriptional regulator
10	1385631-1385671	6.48	MSMEG1292L*	2.4	Dehydrogenase protein
			MSMEG1293R*	4.2	Xanthine/uracil permeases family protein
11	1684231-1684271	64.6	MSMEG1597	2.8	Transcription factor WhiB
12	1832291-1832331	46.5	MSMEG1738	-13.2	Probable conserved transmembrane protein
13	1965171-1965211	19.3	MSMEG1886	No DE	Fatty acid desaturase
14	2000471-2000511	10.9	MSMEG1919	No DE	Transcription factor WhiB
15	2070111-2070151	9.9	MSMEG1987*	120.7	Putative uncharacterised protein
16	2081471-2081511	19.5	MSMEG1999	-2.1	Putative uncharacterised protein
17	2260871-2260911	39.9	MSMEG2183L	2.3	Conserved hypothetical protein
			MSMEG2184R*	38.8	Amino acid permease
18	2414891-2414931	67.9	MSMEG2332	10.1^g^	Amino acid carrier protein
19	2508191-2508231	101.5	MSMEG2425*	98.8	*amtB* Ammonium transporter
20	2592931-2592971	18.6	MSMEG2506*	-4.1	Carboxyvinyl-carboxyphosphonate phosphorylmutase
21	2608351-2608391	171.1	MSMEG2522*	165.9	Efflux ABC transporter, permease protein
22	2612531-2612571	331.2	MSMEG2526	782.4	Copper amine oxidase
23	2655531-2655571	56.3	MSMEG2570*	50.8	Xanthine/uracil permease
24	3048291-3048331	105.9	MSMEG2982*	583.8	Putative periplasmic binding protein
25	3206851-3206891	8.7	MSMEG3131L	-1.44	Polypeptide: AMP-binding protein
			MSMEG3132R	No DE	Polypeptide: DNA-binding protein
26	3237471-3237511	6.5	MSMEG3166	No DE	Enzyme: beta-lactamase
27	3471571-3471611	8.2	MSMEG3400*	228.0	Glutamyl-tRNA(Gln) amidotransferase subunit A
28	4043191-4043231	22.8	MSMEG3975	2.1	Putative regulatory protein, PucR family
29	4069251-4069291	58.9	MSMEG3995	9.7	N-carbomoyl-L-amino acid amidohydrolase
30	4070051-4070091	13.2	MSMEG3996L	8.3	*hydA* Dihydropyrimidinase
			MSMEG3997R	6.5	Regulatory protein, PucR family
31	4082411-4082451	77.2	MSMEG4008*	49.2	Oxidoreductase, 2OG-Fe(II) oxygenase family protein
32	4136531-4136571	7.4	MSMEG4063	No DE	Polypeptide: amidohydrolase
33	4290471-4290511	8.0	MSMEG4206	115.7	Molybdopterin oxidoreductase
34	4374791-4374831	184.7	MSMEG4290	20.0	*glnA* Glutamine synthetase
35	4381891-4381931	49.8	MSMEG4294	12.6	*glnA* Glutamine synthetase, type I
36	4580191-4580231	384.4	MSMEG4501	103.3	Sodium:dicarboxylate symporter
37	4722511-4722551	17.1	MSMEG4635*	102.0	*amtA* Ammonium transporter
38	4726751-4726791	63.6	MSMEG4639*	57.3	Putative uncharacterised protein
39	4729431-4729471	11.1	MSMEG4643	No DE	Resuscitation-promoting factor
40	4729931-4729971	34.4	MSMEG4643	No DE	Resuscitation-promoting factor
41	5183411-5183451	57.5	MSMEG5084*	27.1	Glycosyl transferase, group 2 family protein
42	5440611-5440651	233.9	MSMEG5358	14.9	Acetamidase/Formamidase family protein
43	5442051-5442091	27.2	MSMEG5360*	29.1	Formate/nitrate transporter
44	5651011-5651051	18.6	MSMEG5561	1.40	HPP family protein
45	5840591-5840631	11.6	MSMEG5765	4.1	*glbN* Globin
46	6177591-6177631	31.6	MSMEG6116	24.8	Putative allantoicase
47	6323551-6323591	23.7	MSMEG6259	255.9	*amt1* Ammonium transporter
48	6714291-6714331	16.3	MSMEG6660	8.1	Cytosine/purine/uracil/thiamine/allantoin permease
49	6747051-6747091	9.9	MSMEG6695L	No DE	Cytochrome P450
			MSMEG6697R	No DE	IS1096, tnpA protein
50	6782771-6782811	17.7	MSMEG6735*	128.3	Amino acid permease, putative
51	6865371-6865411	199.7	MSMEG6816	385.3	Molybdopterin oxidoreductase
*52*	*6867931-6867971*	*12.7*	*N/A*	*N/A*	*N/A*
53	6930751-6930791	10.8	MSMEG6881	5.8	Transcriptional regulator, GntR family

^**a**^assigned peak number, ^b^peak coordinates on the *M. smegmatis* genome, ^c^fold enrichment of each peak compared to the input control calculated using SISSRs, ^d^adjacent gene(s) to peak, ^e^fold change in gene expression (WT vs Δ*gln*R) and ^g^fold change in gene expression normalized to *sigA* from qRT-PCR (WT vs Δ*gln*R). L = left and R = right indicates the direction of the gene in relation to GlnR binding where GlnR is proposed to control divergent genes. Genes in operons are denoted by *. Peaks that represent binding sites with no corresponding differential expression of adjacent genes are labelled no DE.

**Table 2:** **Five GlnR binding sites identified in *M. smegmatis *during nitrogen excess**

**Peak**	**Coordinates**	**Peak intensity in N excess**	**Peak intensity in N limitation**	**Gene ID**	**Gene description**
1	1832291 - 1832331	6.7	46.5	MSMEG1738	Probable transmembrane protein
2	2508171 - 2508211	5.38	101.46	MSMEG2425*	*amtB* Ammonium transporter
3	4374771-4374811	6.27	184.71	MSMEG4290	*glnA* Glutamine synthetase
4	4381891 - 4381931	6.94	49.84	MSMEG4294	*glnA *Glutamine synthetase, type I
5	5651011 -5651051	5.51	18.6	MSMEG5561	HPP family protein

Fold enrichment of each GlnR binding sites (peaks) observed in nitrogen excess with peak intensity in nitrogen limitation given for comparison. Genes in operons are denoted by *.

**Figure 1:**
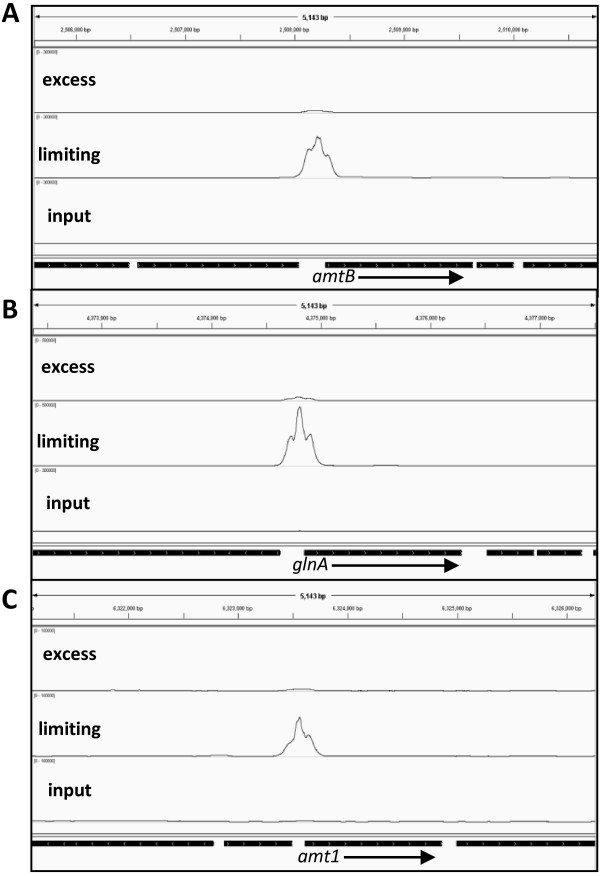
**GlnR binding sites identified by ChIP-seq during nitrogen limitation. **Examples of peaks obtained for genes known to be GlnR-regulated. Binding sites were visualised by aligning the 160 million sequence reads to the *M. smegmatis *genome using IGV. The upper track indicates ChIP-seq data for GlnR immunoprecipitated DNA in nitrogen excess conditions, middle track shows the ChIP-seq data for GlnR immunoprecipitated DNA in nitrogen limiting conditions and the total DNA input control is shown in the bottom track. GlnR binding sites were identified upstream of (**A**) *amtB***, (B**) *glnA1 *and **(C**) *amt1.*

**Figure 2:**
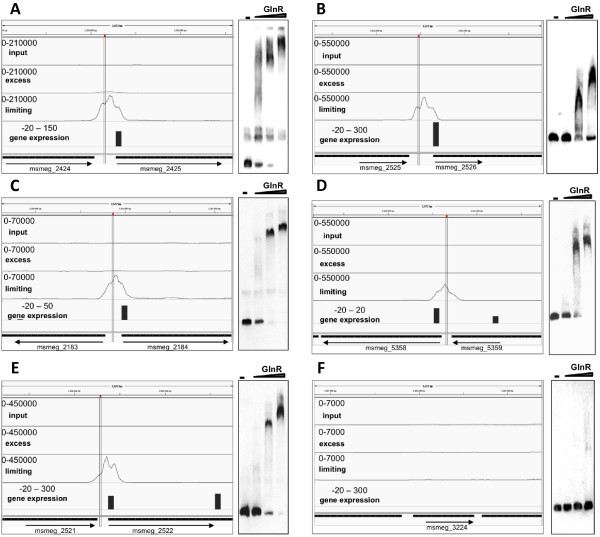
**Novel GlnR binding sites identified upstream of differentially expressed genes, with corresponding EMSA to confirm specific GlnR binding. **EMSA were performed by incubating increasing amounts of His-GlnR recombinant protein with labelled DNA corresponding to the promoter regions of the genes downstream of the GlnR binding site. GlnR binding was visualised in IGV. The top track represents GlnR binding in nitrogen excess, the second track represents GlnR binding in nitrogen limiting conditions , and the third track represents input control DNA. Bar height is representative of fold change in gene expression in the WT compared to the Δ*glnR *mutant in nitrogen limitation. Levels of gene expression are indicated in the bottom track. Vertical lines through the peak indicate GlnR binding sites. **(A**). Peak 9, MSMEG2425 (*amtB*),** (B**). Peak 22, MSMEG2526, **(C**). Peak 17, MSMEG2184,** (D**). Peak 42, MSMEG5358,** (E**). Peak 21, MSMEG2522 and **(F**). Negative control, MSMEG3224.

The identification of the three previously known GlnR binding sites (upstream of *amt1*, *amtB* and *glnA1*) in our ChIP-seq data (Figure 1) validated our approach. However, we used purified GlnR protein and electromobility shift assays (EMSA) to further validate four of the novel GlnR DNA binding regions identified in this study. DNA sequences (200 bp) representing the promoter regions of peak 19 (*amtB*, included as a positive control), peak 17 (MSMEG2184), peak 21 (MSMEG2522), peak 22 (MSMEG2526), and peak 42 (MSMEG5358), all showed specific GlnR binding, with the DNA/protein complex shift dependent on DNA concentration. The promoter region of MSMEG3224, a region not identified as a GlnR binding site in this study and included as a negative control, showed no GlnR binding (Figure 2).

### Delineation of the GlnR regulon during nitrogen limitation

In order to identify the genes controlled directly by GlnR and thus forming the GlnR regulon, we mapped the 53 binding sites onto the profile of transcripts regulated by GlnR during nitrogen limitation, using the Integrated Genome Viewer [35,36], examples of which can be seen in Figure 2 (all 53 binding sites in nitrogen limitation can be viewed in Additional file 3: Figure S2). Forty-four GlnR binding sites corresponded to the differential expression of 103 genes, 91 of which were located in 21 operons (Table 1). Interestingly as well as the 96 genes up-regulated by GlnR during nitrogen limitation, 7 genes (4 singles plus one operon of 3) were down regulated, indicating that GlnR functions as both an activator and repressor of transcription. GlnR binding also controlled the expression of 6 pairs of divergent genes (Table 1).

Genes adjacent to 9 of the 53 GlnR binding sites did not show any differential expression during nitrogen limitation (non-DE binding sites) (Table 1). One of those sites, peak 52, was the only one not in an intergenic region, but located in the 3’ end of MSMEG6817. Additional file 4: Figure S3 shows the binding site identified for peak 52, with little evidence of a clear peak; this is likely a mis-call by the SISSRs programme. To confirm this, rate limiting qPCR was performed using DNA immunoprecipitated from nitrogen limiting and excess conditions, and no enrichment was observed (data not shown), therefore this binding site was excluded from the GlnR regulon. Two binding sites (peaks 18 and 49) were upstream of genes not present in the microarray, therefore these genes were analysed by qRT-PCR. MSMEG2332 (adjacent to peak 18) showed significant differential expression in the WT compared to the Δ*glnR* mutant under nitrogen limitation (10.1 average fold change; p value <0.01; n = 3) and was therefore deemed to be part of the GlnR regulon, but the gene adjacent to peak 49, MSMEG6697, was not differentially expressed (data not shown).

In order to further investigate the other 8 binding sites showing no DE, rate limiting qPCR was performed on immunoprecipitated DNA from cells grown under nitrogen limiting and excess conditions. Additional file 5: Figure S4 shows enrichment of the 8 promoter regions in nitrogen limitation compared to nitrogen excess; GlnR binding to peak 13 was also confirmed by EMSA (Additional file 6: Figure S5). Therefore these 8 peaks may be part of the GlnR regulon, although it would appear that GlnR does not alter transcription of these genes under the conditions tested. The complete GlnR regulon, including these 8 putative members, is provided in Additional file 7: Table S2.

### Identification and analysis of the M. smegmatis GlnR consensus binding motif

**Figure 3:**
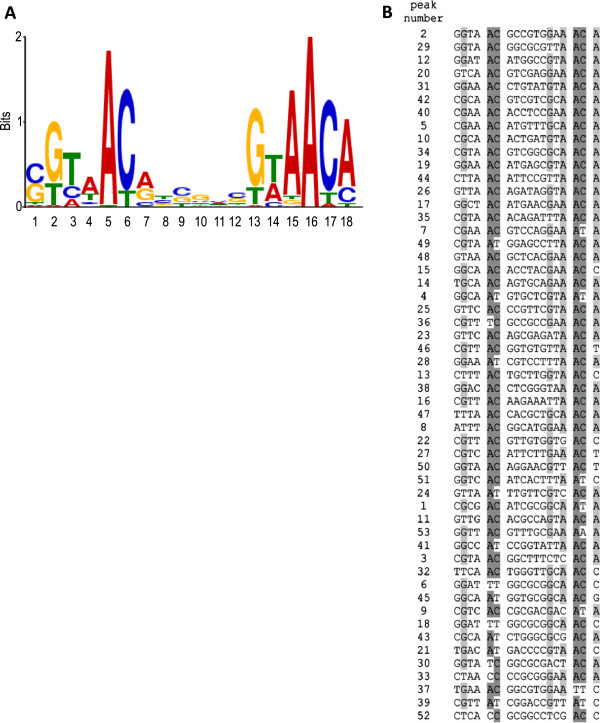
***M. smegmatis *GlnR consensus binding motif derived from the 53 GlnR binding regions identified during nitrogen limitation. **(**A**) MEME generated GlnR motif from 200 bp DNA sequences surrounding the 53 peaks. **(B**) Alignment of sequences in the 53 peaks with the MEME generated motif. Highly conserved residues are highlighted in dark grey, less conserved residues in light grey.

**Figure 4:**
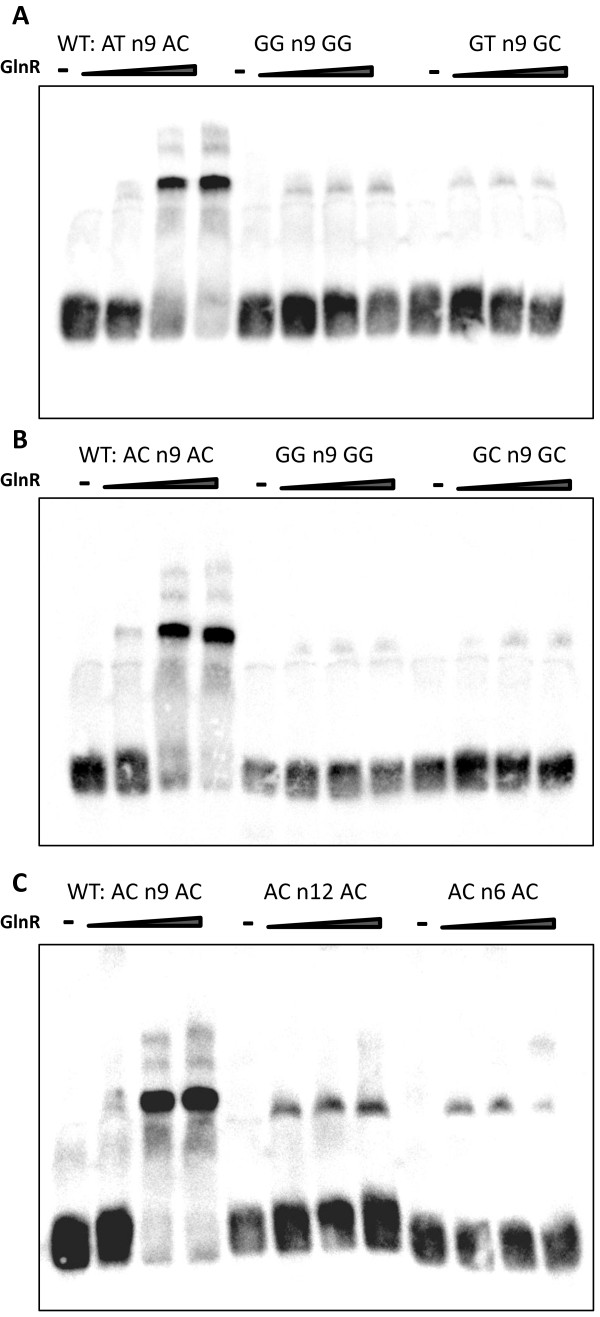
**Mutation of the GlnR binding motif adenosine residues and alteration of the distance between the residues both affect GlnR binding. **(**A**) 30 bp binding site sequence of peak 24, with the wild type sequence and with the AT n9 Ac residues mutated, (**B**) 30 bp binding site sequence of peak 2, with the wild type sequence and the conserved AC n9 AC residues mutated and (**C**) 30 bp binding site of peak 2, with the wild type sequence and the distance between AC n9 AC altered.

The nucleotide sequence (200 bp) for each of the enriched GlnR-binding regions was extracted using the R package Biostrings and submitted to the motif discovery tool Multiple EM (Expectation Maximization) for Motif Elicitation (MEME) [37] to identify a consensus GlnR binding motif. A consensus motif (AC/T-n9-AC) present once in all 53 GlnR binding sites was identified with an E value of 6.5 × 10^-30^ (Figure 3). No direct correlation was observed between either the specific GlnR binding sequence, or the proximity of the binding site to a gene start site, and the level of gene expression (Additional file 8: Table S1). To identify key residues required for specific GlnR binding we mutated the highly conserved AC-n9-AC and AT-n9-AC DNA binding motifs. Figure 4 shows that the highly conserved adenosine residues in the motif are critical as GlnR binding is abolished when these residues are mutated. Substitution of the AC dinucleotide, with either GG or GC, and the AT dinucleotide, with GG or GT, completely abolished GlnR binding (Figure 4A and 4B). The 9 base pair distance between these key adenosine residues was also investigated, and we found either increasing this to 12 nucleotides or decreasing it to 6 base pairs diminished GlnR binding (Figure 4C).

## Discussion

In this report we have combined transcriptomics and ChIP-seq to delineate the regulon controlled by the OmpR-type response regulator GlnR and to investigate the role *M. smegmatis* GlnR plays in regulating the transcriptomic response to nitrogen limitation.

### Delineation of the GlnR regulon

We compared the global expression profile of *M. smegmatis* wild type to a Δ*glnR* deletion mutant during nitrogen limitation, and found over 680 genes were significantly differentially expressed (Suppl Data File S1), with 392 genes up regulated and 291 down regulated. This large number of DE genes (approximately 10% of the genome) shows that a major GlnR-dependent transcriptomic response is initiated by *M. smegmatis* during nitrogen limitation. However, not all of these genes are directly regulated by GlnR, since the set includes 7 genes themselves annotated as response regulators, making the genes under the control of these other response regulators only indirectly controlled by GlnR. To identify the directly controlled genes we used ChIP-seq to identify GlnR-DNA binding sites and combined this with the transcriptional data.

ChIP-seq identified 53 GlnR binding sites in *M. smegmatis* during nitrogen limitation. Forty-four of these are upstream of GlnR-dependant transcripts identified in our microarray analysis; controlling 103 genes in total, including those predicted to be in operons (Additional file 7: Table S2). GlnR can act as a dual transcriptional regulator, both activating and repressing gene expression, as has been shown for other OmpR type regulators [29,38], with approximately 7% of the genes in the *M. smegmatis* GlnR regulon repressed during nitrogen limitation. GlnR also controlled the expression of 6 pairs of divergently transcribed genes (Table 1). The genes associated with two GlnR regulated genes (peaks 18 and 49) were missing absent from the microarray, but qRT-PCR showed MSMEG2332, encoding an amino acid carrier protein, was under GlnR control (increasing the regulon to 103 genes), whereas MSMEG6697, encoding a putative *tnpA* transposase, was not.

The remaining 9 GlnR-binding sites were not associated with GlnR-regulated transcripts. Of these, peak 52 is located within a coding region and visual inspection of the region indicated it was not a true peak (Additional file 4: Figure S4). This was also confirmed by rate limiting qPCR. Peaks 39 and 40 both appear in the promoter region of MSMEG4643, possibly indicating multiple GlnR binding sites for the regulation of this gene; rate limiting qPCR showed enrichment of this and the other 7 binding sites (Additional file 5: Figure S2). There are 10 genes downstream of these 8 GlnR-binding sites, 3 of which are down regulated, but less than 2 fold. The remaining 7 do not show any significant DE, but are included as putative members of the GlnR regulon by virtue of the binding sites upstream (Suppl Data File S2). In summary, we have demonstrated that the entire GlnR regulon during nitrogen limitation consists of a minimum of 103 genes.

GlnR also binds to 5 sites during nitrogen excess (Table 2), but with significantly lower peak intensity values than observed in nitrogen limitation. These genes may be required for general nitrogen metabolism under all conditions, with increased gene expression required during nitrogen limitation. We have confirmed this experimentally for one of these genes, *glnA1*, which shows a higher basal level of transcription in nitrogen excess compared to other nitrogen genes, but is still induced upon nitrogen limitation [32]. The absence of other GlnR DNA binding sites during nitrogen excess is intriguing, since *glnR* transcript levels do not differ significantly under high versus low nitrogen levels [31,32], yet under nitrogen stress GlnR protein binds to 52 sites. This could imply that the GlnR present in nitrogen-rich cells is inactive and is activated during nitrogen limitation, perhaps due to a post-translational modification (such as phosphorylation) and/or the binding of small molecules. Either of these processes could promote dimerization during nitrogen limitation to stabilise the protein, resulting in DNA binding. Alternatively GlnR could be sequestered in the cell, perhaps to the cell membrane [39], which would permit a rapid response to nitrogen stress. Studies to investigate how GlnR is activated during nitrogen limitation are in progress.

### Role of GlnR in primary nitrogen metabolism

As expected, genes that known or predicted to be involved in nitrogen metabolism form the majority in the GlnR regulon. We confirmed that the three ammonium transporters (*amt1*, *amtA* and the *amtB-glnK-glnD* operon) are GlnR-regulated during nitrogen limitation [31,32]; this presumably allows the cell to scavenge available ammonium from the surrounding environment. GS/GOGAT is the main ammonium assimilation pathway in most bacteria and the other members of this pathway (*glnA1*, *gltB* and *gltD*) are also up-regulated by GlnR. Interestingly the GDH enzyme (MSMEG5442), which is active until nitrogen becomes limiting, is not GlnR regulated. Two additional GDH homologs have been proposed (MSMEG4699 and MSMEG6272) [19], but neither are controlled by GlnR, so the mechanisms reducing the activity or levels of this enzyme in nitrogen limitation remain unknown.

Glutamine synthetase (GS) is a key nitrogen metabolism enzyme, identified as a potential drug target in *M. tuberculosis*[40-42]. Four GS are present in mycobacteria (*glnA1-glnA4*), with *M. smegmatis* containing at least 10 genes annotated as putative glutamine synthetases [14]. The *glnA1* and *glnA2* genes are found in all mycobacterial genomes together with *glnE*, which regulates glutamine synthetase activity [24]. Here we show that both *glnA1* (MSMEG4290) and *glnA2* (MSMEG4294) are under GlnR control but none of the other 8 GS homologs are GlnR regulated and the function of these enzymes is unknown.

### Role of GlnR in nitrogen scavenging

The largest category of genes in the GlnR regulon is nitrogen scavenging. This is logical from an evolutionary perspective, as the soil dwelling *M. smegmatis* encounters various nitrogen sources in the environment and must compete with other soil microbes for nutrients. Twenty-seven genes (over 25% of the GlnR regulon) encode nitrogen transporter and binding proteins. In addition to the three ammonium transporters, uptake systems for nitrate/nitrite (MSMEG0433), urea (MSMEG2978–2982), and amino acids/ peptides (MSMEG0781, MSMEG1052, MSMEG2522 and MSMEG 2524) are all up-regulated by GlnR in nitrogen limitation. The *M. smegmatis* genome also encodes enzymes involved in the complete degradation of urea to ammonium suggesting that urea is an important alternative nitrogen source during limiting conditions, however although these urea hydrolysis genes (MSMEG3623-3627) are up-regulated in *M. smegmatis* during nitrogen limitation (K. Williams, unpublished data), this is not controlled by GlnR.

A similar situation is observed for nitrate/nitrite uptake and assimilation in that *M. smegmatis* contains two nitrate/nitrite transporters, NarK (MSMEG5141) and NarK3 (MSMEG0433), with only NarK3 up-regulated by GlnR; NarK is constitutively expressed during nitrogen limitation (K. Williams, unpublished data). For nitrate to be assimilated it must be converted to ammonium via a two-step process; reduction of nitrate to nitrite by nitrate reductase (NarGHJI; MSMEG5137-5140) followed by reduction of nitrite to ammonium by nitrite reductase (NirBD; MSMEG0427-0428). As reported previously, and confirmed in this study, the nitrite reductase NirBD enzyme is up regulated by GlnR in nitrogen limitation [32], but the nitrate reductase enzyme is not. Therefore the uptake and assimilation of nitrite, not nitrate, appears to be an important nitrogen stress response in *M. smegmatis*. In this study we also identified a GlnR regulated transcriptional regulator, NnaR (MSMEG0432), the homologue of which in *S. coelicolor* is essential for GlnR function and growth on nitrate [43]. However, the precise role of this regulator and nitrate/nitrite respiration in the nitrogen stress response in *M. smegmatis* requires further investigation.

One further intriguing observation is that GlnR increases the expression of genes encoding enzymes predicted to be involved in processes that break down cellular components into ammonium. For example, an amine oxidase (MSMEG2526) which break down amines into ammonia and an aldehyde, a urea amidolyase (MSMEG2187) that converts urea to CO_2_ and ammonia, a deaminase (MSMEG1298) which breaks down nucleotides into nucleosides and ammonia, and several hydrolases which act on carbon-nitrogen bonds (MSMEG0571, MSMEG1078, MSMEG2189, MSMEG6733) are all GlnR regulated. These cellular components may either originate from other organisms in the environment, or from within the cell. For example, *E. coli* can use its own peptidoglycan D-Ala-D-Ala as a nitrogen source [4]. Therefore it is conceivable that in times of extreme nitrogen austerity, mycobacteria could use cellular components to provide the ammonium required for growth and survival in the short term until nitrogen again becomes available.

### Determination of the GlnR binding site motif and Key residues

MEME identified a 17 bp consensus GlnR binding sequence of Gn_2_AC-n_6_GnAACA present once in all the GlnR binding sites. Streptomyces has a 16 bp GlnR DNA binding motif [29,30], which is similar to the one identified here. Tiffert *et al.* (2008) proposed the existence of two GlnR motifs within the binding motif in *S. coelicolor*; an “a site” (gTnAc) and a highly conserved “b site” (GaAAc)–located 6 bp apart–in which the “b site” has a higher affinity for GlnR than the “a site”. However, the motif identified in *S. venezuelae*, GTnAC-n_6_-GTnAC only contains two copies of an “a site”. The *M. smegmatis* GlnR binding motif contains two different sites, a variable a-type site (Gn_2_AC) separated by 6 bp from a “b site” (GnAAC) that is highly similar to the *S. coelicolor* “b site” sequence. Pullan *et al.* suggested that conservation of the “b site” might be indicative of strong GlnR regulation in terms of gene expression. However, we did not find any correlation between the presence or absence of the b-site. For example MSMEG4501 and MSMEG5358 have the b-site, whilst MSMEG6816, MSMEG2982 and MSMEG2526 do not, yet all exhibit similar differential gene expression (Additional file 8: Table S1). Consequently the conserved “b site” is not the sole determinant of the strength of GlnR regulation, and additional transcription factors may contribute. However, there is a highly conserved AC-n_9_-AC motif present in both *M. smegmatis* and *Streptomyces*, and we have shown that spacing between the dinucleotides, as well as the presence of adenosine are both crucial for GlnR binding. The 9 bp distance between these adenosine residues represents one turn of the major groove of the DNA helix, ensuring both are available to interact with GlnR.

## Conclusions

In summary, we have demonstrated that GlnR is the global nitrogen response regulator in *M. smegmatis*, directly regulating the expression of more than 100 genes. GlnR controls key nitrogen stress survival processes including primary nitrogen metabolism pathways, the ability to utilise nitrate and urea as alternative nitrogen sources, and the potential to use cellular components to provide a source of ammonium. Although we have shown that GlnR plays a central role in nitrogen metabolism in mycobacteria, several questions remain unanswered. For instance, the mechanism of activation of GlnR is not known. GlnR is an orphan response regulator and the corresponding kinase (if one exists) has not yet been identified. Investigations are also in progress to identify the signal(s) indicating cellular nitrogen status and the mechanisms by which this signal is detected and translated into GlnR activation. These studies are important for furthering our understanding of how mycobacteria survive nutrient limiting conditions.

## Methods

### Growth conditions

*M. smegmatis mc*^*2*^*155* wild type (ATCC 700084) [44] and *M. smegmatis mc*^*2*^*155* Δ*glnR*[32] were used in this study. The *M. smegmatis* Δ*glnR* mutant was constructed by recombineering [45] replacing the entire *glnR* gene with a hygromycin resistance cassette [32]. *M. smegmatis* was grown aerobically in Middlebrook 7H9 liquid broth (supplemented with 0.2% glycerol, 0.05% Tween 80 and 10% OADC) at 37°C, 180 rpm. Optimised nitrogen limiting conditions have been described [25,32]. Briefly, an overnight culture of *M. smegmatis* was washed twice in nitrogen free Sauton’s medium (0.05% (w/v) KH_2_PO_4_, 0.05% (w/v) MgSO_4_, 0.2% (w/v) citric acid, 0.005% (w/v) ferric citrate, 0.2% (v/v) glycerol, 0.0001% (v/v) ZnSO_4_, 0.015% (v/v) Tyloxapol) and inoculated into Sauton’s nitrogen free medium, supplemented with 1 mM (nitrogen limiting) or 30 mM (nitrogen excess) ammonium sulphate (Ultra pure; Sigma) to a starting OD_600_ of 0.08 (Biochrom). Growth was monitored by OD_600_. Ammonium ions in the culture medium were quantified using an AquaQuant Ammonium detection kit (Merck).

### Purification of recombinant GlnR

The *M. smegmatis glnR* (MSMEG5784) and *M. tuberculosis glnR* (Rv0818) genes were PCR amplified from genomic DNA using specific primers (Additional file 9: Table S2). Digested fragments were cloned into pET28b (Novagen) to construct tagged protein with His-tag at the N-terminus and ligations transformed into BL21 (DE3) pLysS *E.coli* (Promega). Recombinant *E. coli* strains were cultivated at 37°C in LB broth supplemented with 50 μg ml^-1^ Kanamycin until mid-log phase, when 1 mM IPTG was added and incubation continued at 20°C for 3 hours. Cells were harvested, centrifuged and pellet re-suspended in lysis buffer (PBS, EDTA-free protease inhibitor tablet (Roche), 100 μg/ml lysozyme, 85.5 units deoxyribonuclease I (*Invitrogen*)) before probe sonication. Soluble protein extract was loaded onto a pre-charged nickel column (GE Healthcare) and purified via affinity chromatography using a FPLC AKTA Purifier (GE Healthcare). Pooled fractions containing His-GlnR were dialysed into storage buffer (10 mM Tris–HCl pH 8, 50 mM NaCl, 20% (v/v) glycerol, 0.1 mM EDTA) for antibody production or (10 mM Tris–HCl pH 8, 50 mM NaCl, 5% (v/v) glycerol) for gel shift assays. Protein concentration was determined using the BCA protein assay kit (Pierce) according to manufacturer’s instructions.

### Generation of GlnR polyclonal antibody and purification

Purified *M. tuberculosis* His-GlnR was used to raise polyclonal rabbit antibody (Eurogentec, Belgium). Polyclonal anti-GlnR serum was affinity purified using recombinant *M. smegmatis* His-GlnR. His-GlnR (50 μg) was separated via SDS PAGE, transferred to a nitrocellulose membrane and visualised with Ponceau S (Sigma). A membrane slice containing His-GlnR was blocked (PBS with 5% milk powder) for 1 hr at RT, followed by incubation overnight at 4°C with 5 ml serum diluted in 25 ml Block. The membrane was washed in PBS before the antibody was eluted with 100 mM glycine pH 2.7. The pH of the eluate was neutralised with 1.5 M Tris–HCl pH 8.8. Purified antibody was dialysed against PBS and stored at -20°C.

### Electromobility shift assay (EMSA)

To analyse GlnR binding to gene promoter regions, DNA fragments were PCR amplified from *M. smegmatis* genomic DNA and used in electromobility shift assays (Additional file 9: Table S4). To identify key nucleotides required for GlnR binding, complementary oligonucleotides were designed to mutate or alter the distance of key residues and annealed to generate DNA fragments for EMSAs (Additional file 9: Table S4). DNA fragments were labelled using a DIG Oligonucleotide 3’-End Labelling Kit (Roche). DNA:protein binding reactions contained 0.4 ng of labelled DNA, 0.5 μg poly d(A-T), 0–0.9 μg His-GlnR, 25 mM Hepes (pH 7.9), 150 mM NaCl, 2.5 mM MgCl_2_. The reaction mixture was incubated at 37°C for 15 min, before separation on a pre-run 6% DNA retardation gel (Invitrogen). Labelled DNA was transferred to a nylon membrane (Amersham) using a wet transfer XCell SureLock Blot module (*Invitrogen*). DNA was cross-linked to the membrane with a UV Stratalinker and membrane development proceeded according to manufacturer’s instructions (Roche). Bands were visualised using a LAS-3000 Fuji imager.

### Rate-limiting PCR

To identify enrichment in GlnR-immunoprecipitated DNA a rate-limiting PCR was performed. DNA was immunoprecipitated and purified as described under chromatin-immunoprecipitation. DNA sequences were amplified using primers listed in Additional file 9: Table S2. Reaction mixtures consisted of GlnR-immunoprecipitated DNA (0.3 ng), 1 × BioMix (Bioline), 1 μM of each primer and 5% (v/v) dimethyl sulfoxide (DMSO; Sigma). PCR was carried out in a thermocyler T3000 (Biometra); 95°C for 5 min, 23 cycles of 95°C 30 sec, 55°C 30 sec, 72°C 1 min, with final extension 72°C for 8 min. DNA was visualised on a 2% agarose gel.

### RNA isolation

*M. smegmatis* strains were grown in triplicate in nitrogen limiting conditions until external nitrogen was depleted. Total RNA was extracted from exponentially growing cells using the GTC/Trizol method [46]. Extracted RNA was purified using the RNeasy kit (Qiagen) and residual DNA removed by TURBO DNA-free (Ambion Life Technologies) treatment. Superase (ABI Life Technologies) was added and RNA was stored at -80°C. Quality and quantity of RNA was determined using a Bio-analyser (Agilent).

### Quantitative real-time PCR (qRT-PCR)

cDNA was amplified from 100 ng of RNA using the SuperScript III First-Strand Synthesis SuperMix (Invitrogen). qRT-PCR reactions were carried out in a final volume of 10 μl (1 μl of cDNA, 5 μl of TaqMan PCR master mix (Applied Biosystems), 0.5 μl TaqMan probe (Applied Biosystems)). Amplification was performed on an Applied Biosystems 7500 Real-Time System (50°C 5 min, 95°C 10 min, and 40 cycles of 95°C 15 sec, 60°C 1 min). Linear amplification and amplification efficiencies for each TaqMan primer/probe was determined. Real-time analysis was performed on RNA from three independent cultures and quantification of *sigA* expression served as an internal control. Fold change was calculated as a ratio of the arbitrary expression units, standardised to *sigA*. Statistical analysis of data was performed using a Student’s *t*-test, a *P* value of ≤ 0.01 was considered significant. Primers and Taqman probe sequences for each gene studied are given in Additional file 10: Table S5.

### Preparation of labelled cDNA from total RNA

Labelled cDNA was prepared from 1 μg total RNA using Cy3-dCTP (GE Healthcare) and SuperScript II reverse transcriptase with random hexamer primers (*Invitrogen*). Labelled cDNA was purified by Qiagen MinElute column, combined with 10× CGH blocking agent and 2× Hi-RPM hybridisation buffer (Agilent) and heated (95°C for 5 min) prior to loading onto microarray slides. Slides were incubated overnight in an Agilent rotating oven at 65°C, 20 rpm. After hybridization slides were washed (5 min at room temperature) with CGH Wash Buffer 1 (Agilent) and 1 min at 37°C with CGH Wash buffer 2 (Agilent). Slides were scanned immediately, using an Agilent High Resolution Microarray Scanner, at 2 μm resolution, 100% PMT. Scanned images were quantified using Feature Extraction software v 10.7.3.1.

### Microarray design

The microarray was constructed by determining all unique genes from the 6887 chromosomal predicted coding sequences of *M. smegmatis* strain MC2 155, downloaded from Ensembl Bacteria Release 5 (http://bacteria.ensembl.org/). Multiple optimal hybridisation 60-mer oligonucleotide sequences were designed (Oxford Gene Technologies), from which a minimal non-redundant subset of oligonucleotides were selected with target coverage of three 60-mers per gene. Arrays were manufactured on the Inkjet in-situ synthesized platform (Agilent) using the 8×60 k format.

### Statistical analyses of differential gene expression

Statistical analyses of the gene expression data was carried out using the statistical analysis software environment R together with packages available as part of the Bioconductor project (http://www.bioconductor.org). Data generated from the Agilent Feature Extraction software for each sample was imported into R. Replicate probes were mean summarised and quantile normalised using the pre-process Core R package. The limma R package [47] was used to compute empirical Bayes moderated *t*-statistics to identify differentially expressed genes between time points. Generated p-values were corrected for multiple testing using the Benjamini and Hochberg False Discovery Rate. A corrected p-value cut-off of less than 0.01 was used to determine significant differential expression.

### Chromatin-immunoprecipitation (ChIP)

#### Cell preparation and cross-linking

*M. smegmatis* was grown as specified before cross-linking with the addition of formaldehyde (Sigma) (1% (v/v)). Cross-linking proceeded for 20 min at 37°C, before glycine addition (125 mM) for 5 min at 37°C. Cells were harvested and washed twice with TBS. The pellet was frozen at -80°C until required. For DNA fragmentation the pellet was re-suspended in immunoprecipitation (IP) buffer (50 mM HEPES-KOH pH 7.5, 150 mM NaCl, 1 mM EDTA, 1% (v/v) Triton X-100, 0.1% (w/v) Na deoxycholate, 0.1% (w/v) SDS) supplemented with EDTA- free complete protease inhibitor cocktail (Roche), before sonication [100% amp, 30 sec pulses for 10 min] (Misonix Ultrasonic Processor S4000). Debris was removed by centrifugation and the supernatant recovered. A 100 μl sample was taken and stored at -20°C, this served as the ‘input’ sample and was subjected to protein degradation as described. The rest of the sample was used for immunoprecipitation.

#### Immunoprecipitation and elution of DNA

Purified rabbit anti-GlnR polyclonal antibody was added to the sonicated extract and incubated overnight at 4°C. Sheep anti-rabbit IgG Dynal beads (*Invitrogen*) were prepared by washing 2× PBS and 2× IP buffer, before bead saturation overnight in blocking solution (IP buffer, EDTA-free protease inhibitor tablet, 1 mg/ml BSA). Blocking solution was removed and bead-sonicated sample complex incubated for 3 hours at 4°C. To harvest the bead-antibody-DNA complex a magnet was used. The complex was then subject to a series of washing steps; 2× IP buffer, IP buffer plus 500 mM NaCl, wash II (10 mM Tris pH 8, 250 mM LiCl, 1 mM EDTA, 0.5% Nonidet-P40, 0.5% (w/v) Na deoxycholate), TE buffer (50 mM Tris, 10 mM EDTA pH 7.5). Elution of DNA was performed by addition of elution buffer (50 mM Tris–HCl pH 7.5, 10 mM EDTA, 1% (w/v) SDS) and incubation at 65°C for 40 min. Beads were separated by magnetism and the supernatant harvested. Elucidate was diluted 2-fold in nuclease free H_2_O (Qiagen), followed by protein degradation with the addition of 4 mg/ml Pronase and incubated: 42°C for 2 hours and 65°C for 6 hours. DNA was subsequently purified using the Qiagen MiniElute kit and DNA quantified using the dsDNA Qubit (*Invitrogen*).

#### Library preparation

DNA was prepared for next generation sequencing using the Illumina ChIP-seq DNA sample preparation kit according to the manufacturer’s protocol, with the addition of a second gel extraction step after PCR amplification, to remove excess primer dimers. DNA size and purity was confirmed by DNA Bioanalyser (Agilent) and sequencing conducted on an Illumina HiSeq2000 sequencer (MRC Clinical Sciences Centre, Hammersmith). All sequencing data have been deposited in ArrayExpress (accession number E-MTAB-1456).

#### Supporting data

The full microarray design is available in BμG@Sbase (A-BUGS-39) and also in ArrayExpress (ArrayExpress: A-BUGS-39). Fully annotated microarray data have been deposited in BμG@Sbase (accession number E-BUGS-143; http://bugs.sgul.ac.uk/E-BUGS-143) and also ArrayExpress (accession number E-BUGS-143). The other data sets supporting the results of this article are included within the article and its additional files.

## Competing interests

The authors declare that they have no competing interests.

## Authors’ contributions

VAJ and KJW performed the experiments and carried out the genome analysis, participated in the design of the study and drafted the manuscript. GRB carried out the bioinformatics analysis. BDR conceived the study, participated in its design and coordination, and drafted the manuscript. All authors read and approved the final manuscript.

## Supplementary Material

Additional file 1: Table S1 List of genes displaying differential expression by microarray during nitrogen limitation, comparing *M. smegmatis *WT vs Δ*glnR.* Click here for file

Additional file 2: Figure S1 Rate limiting qPCR confirmed enrichment of known GlnR regulated genes in nitrogen limiting conditions. (A) Promoter region of *glnA1, *(B) Promoter region of *nirB* and (C) Promoter region of MSMEG3224 (negative control). Rate-limiting PCR involving 23 cycles of amplification, with 0.3 ng of GlnR-immunoprecipitated DNA from nitrogen excess and limiting conditions. Input-excess and input-limiting represents the total DNA prior to immunoprecipitation from the excess and limiting samples respectively. Click here for file

Additional file 3: Figure S2 Screen shots from IGV of all 53 GlnR binding sites identified by ChIP-seq. Click here for file

Additional file 4: Figure S3 Screenshot from IGV showing peaks 51 and 52 and highlighting the mis-calling of peak 52 by SISSRs. Binding data was visualised using IGV. Upper track indicates ChIP-seq data from the Input sample representing the total DNA, middle track is nitrogen excess conditions and then ChIP-seq data from nitrogen limiting conditions. Aligned to the bottom track is the SISSRs value for the peaks highlighted by the vertical black bars. Click here for file

Additional file 5: Figure S4 Rate limiting qPCR confirmed enrichment of the 8 putative GlnR binding sites during nitrogen limitation. (A) Promoter region of MSMEG3224 (negative control), (B) Promoter region of peak 13, (C) Promoter region of peak 14, (D) Promoter region of peak 26, (E) Promoter region of peak 32, (F) Promoter region of peak 39, (G) Promoter region of peak 40, (H) Promoter region of peak 44 and (I) Promoter region of peak 49. Rate-limiting PCR involving 23 cycles of amplification, with 0.3 ng of GlnR-immunoprecipitated DNA from nitrogen excess and limiting conditions. Input excess and input limiting represents the total DNA subject to immunoprecipitation from the excess and limiting samples respectively. Click here for file

Additional file 6: Figure S5 Confirmation of specific GlnR binding to the 200 bp region representing peak 13 by EMSA with the corresponding peak in nitrogen limiting conditions in IGV. EMSAs were performed by incubating increasing amounts of His-GlnR recombinant protein with labelled DNA corresponding to the GlnR binding site peak 13. The addition of non-specific DNA did not affect GlnR binding, confirming this as a specific GlnR binding site. GlnR binding was visualised in IGV. Upper track indicates ChIP-seq data from the Input sample representing the total DNA, middle track is nitrogen excess conditions and the ChIP-seq data from nitrogen limiting conditions aligned at the third track. Levels of gene expression are indicated in the bottom track. Vertical line through the peak indicates the GlnR binding site. Click here for file

Additional file 7: Table S2 Complete list of genes in the M. smegmatis GlnR regulon. Click here for file

Additional file 8: Table S3 MEME-derived GlnR consensus binding site with corresponding ChIP-seq peak intensity and fold change in gene expression. Click here for file

Additional file 9: Table S4 Primer sequences used in this study. Click here for file

Additional file 10: Table S5 Custom Taqman *M. smegmatis *gene expression primer and probe sequences used in this study. Click here for file
