# Supplementary material for: Genome wide analysis of the complete GlnR nitrogen-response regulon in Mycobacterium smegmatis
Source: BMC Genomics. 2013 May 4;14:301. doi: 10.1186/1471-2164-14-301 (PMC3662644; doi:10.1186/1471-2164-14-301)
Supplement: Additional file 2: Figure S1 — Rate limiting qPCR confirmed enrichment of known GlnR regulated genes in nitrogen limiting conditions. (A) Promoter region of glnA1, (B) Promoter region of nirB and (C) Promoter region of MSMEG3224 (negative control). Rate-limiting PCR involving 23 cycles of amplification, with 0.3 ng of GlnR-immunoprecipitated DNA from nitrogen excess and limiting conditions. Input-excess and input-limiting represents the total DNA prior to immunoprecipitation from the excess and limiting samples respectively. [file 1471-2164-14-301-S2.pptx]

## Slide 1
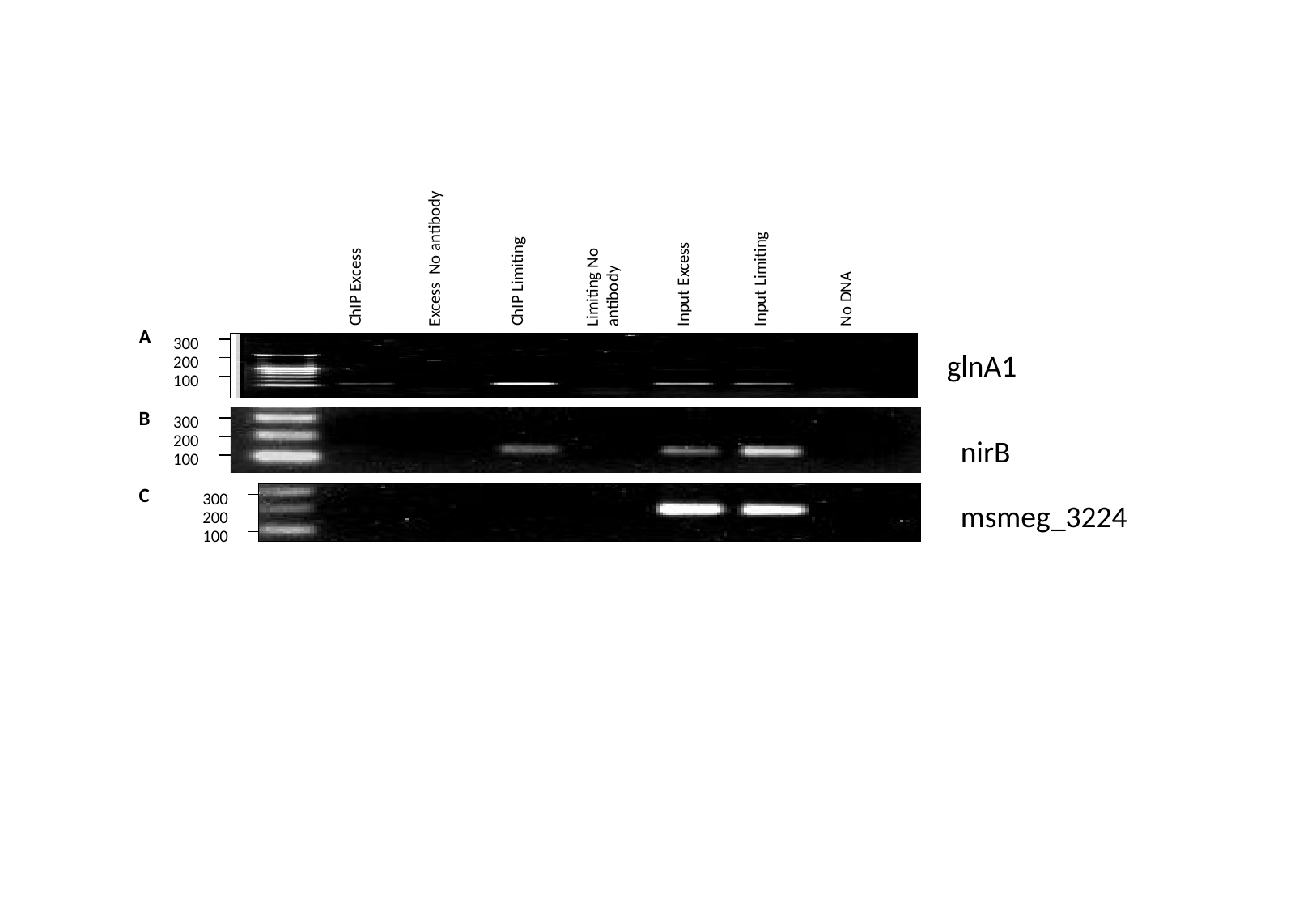

Limiting No antibody
Excess No antibody
ChIP Limiting
ChIP Excess
Input Excess
Input Limiting
No DNA
A
300
glnA1
200
100
B
300
200
nirB
100
C
300
msmeg_3224
200
100
