# Supplementary material for: Genome wide analysis of the complete GlnR nitrogen-response regulon in Mycobacterium smegmatis
Source: BMC Genomics. 2013 May 4;14:301. doi: 10.1186/1471-2164-14-301 (PMC3662644; doi:10.1186/1471-2164-14-301)
Supplement: Additional file 3: Figure S2 — Screen shots from IGV of all 53 GlnR binding sites identified by ChIP-seq. [file 1471-2164-14-301-S3.pptx]

## Slide 1
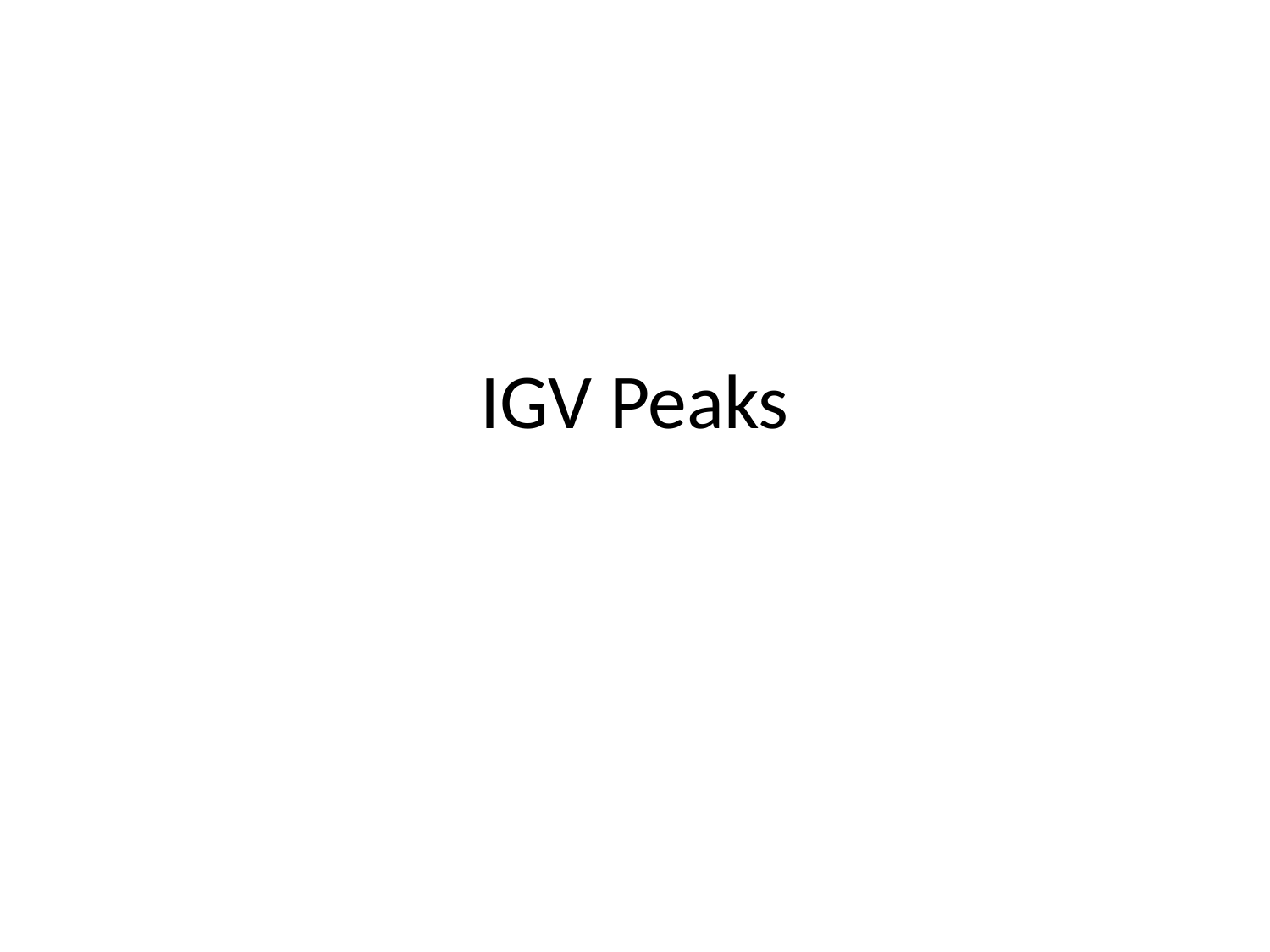

# IGV Peaks

## Slide 2
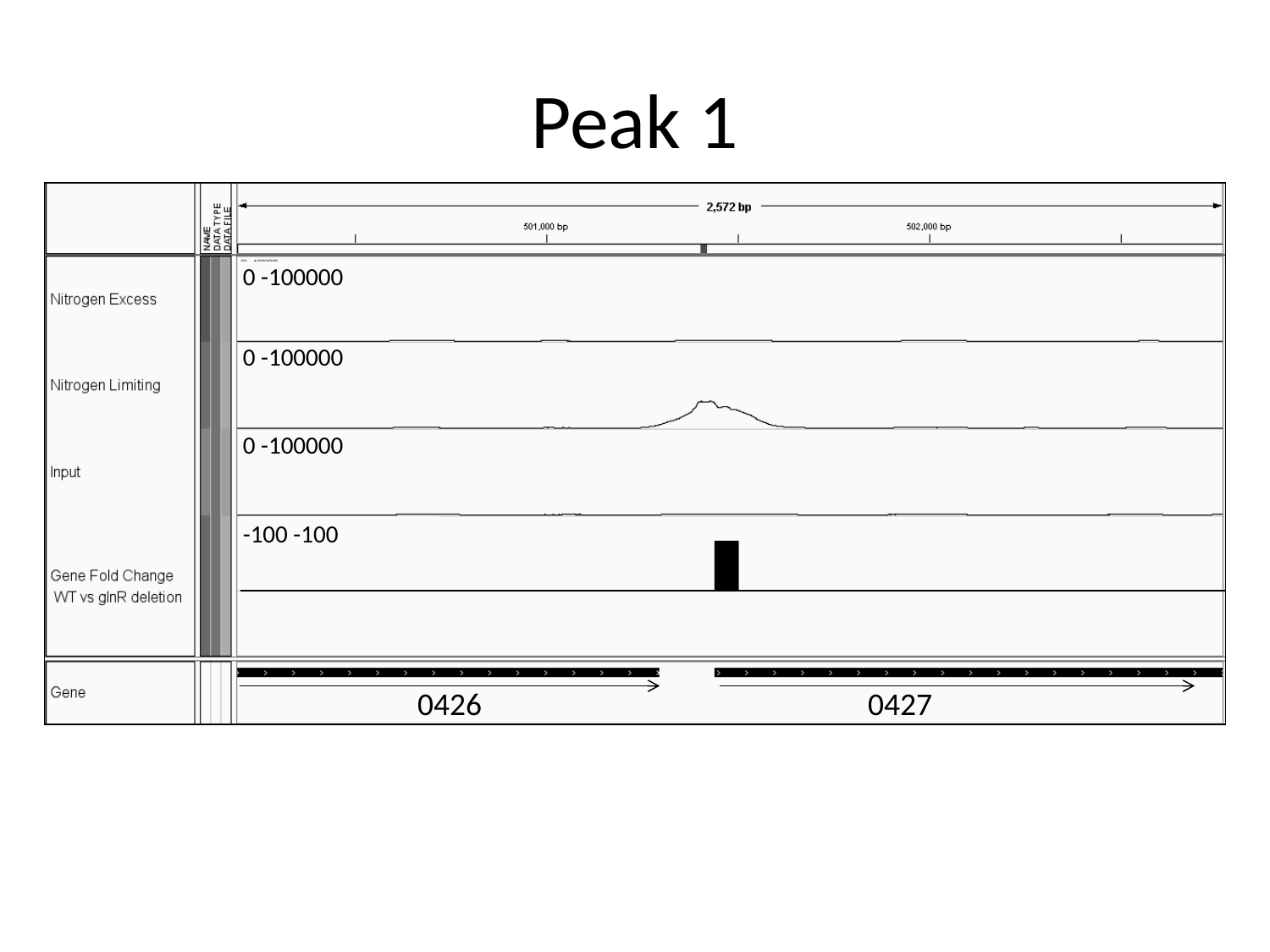

# Peak 1
0 -100000
0 -100000
0 -100000
-100 -100
0426
0427

## Slide 3
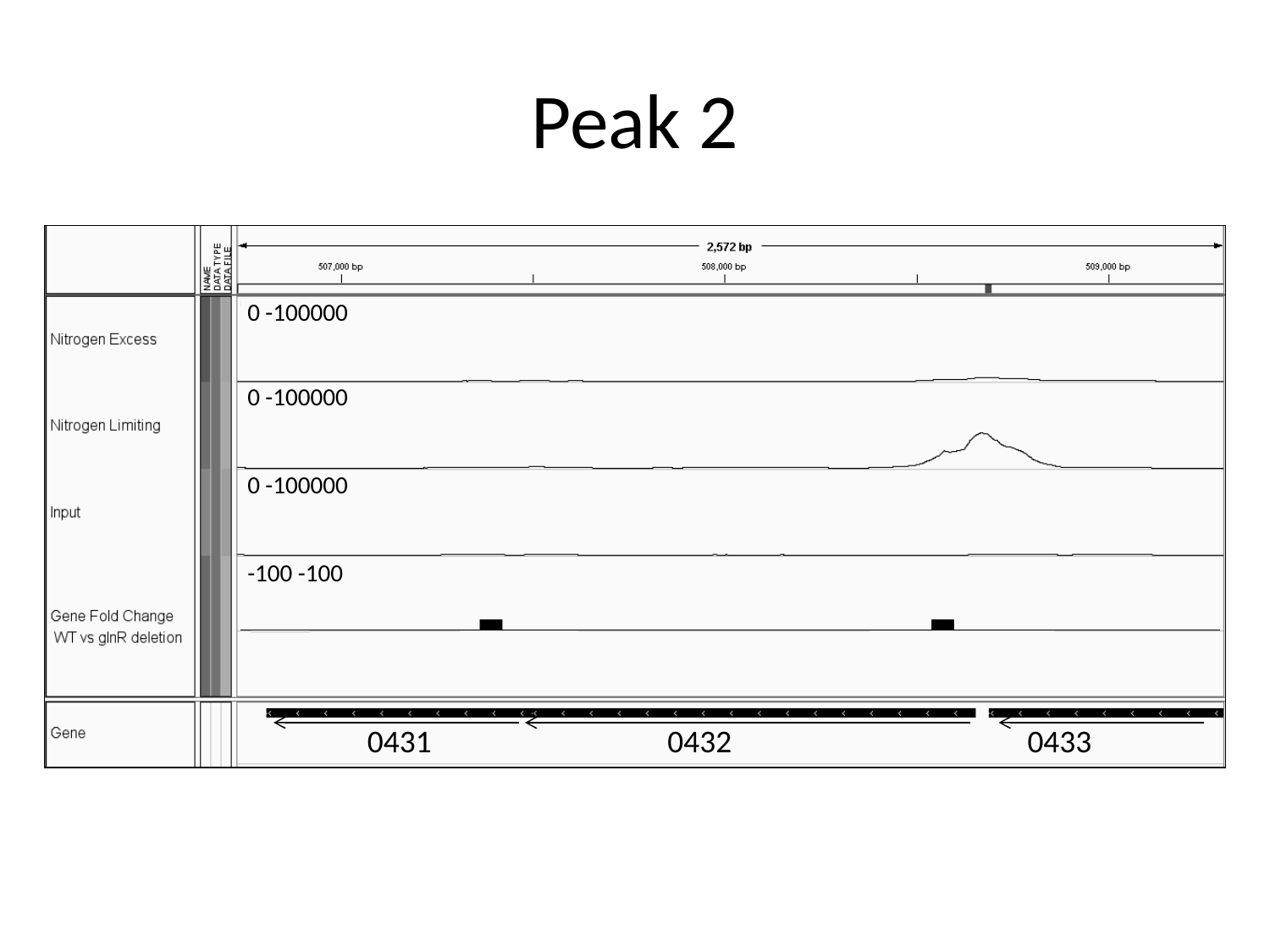

# Peak 2
0 -100000
0 -100000
0 -100000
-100 -100
0431
0432
0433

## Slide 4
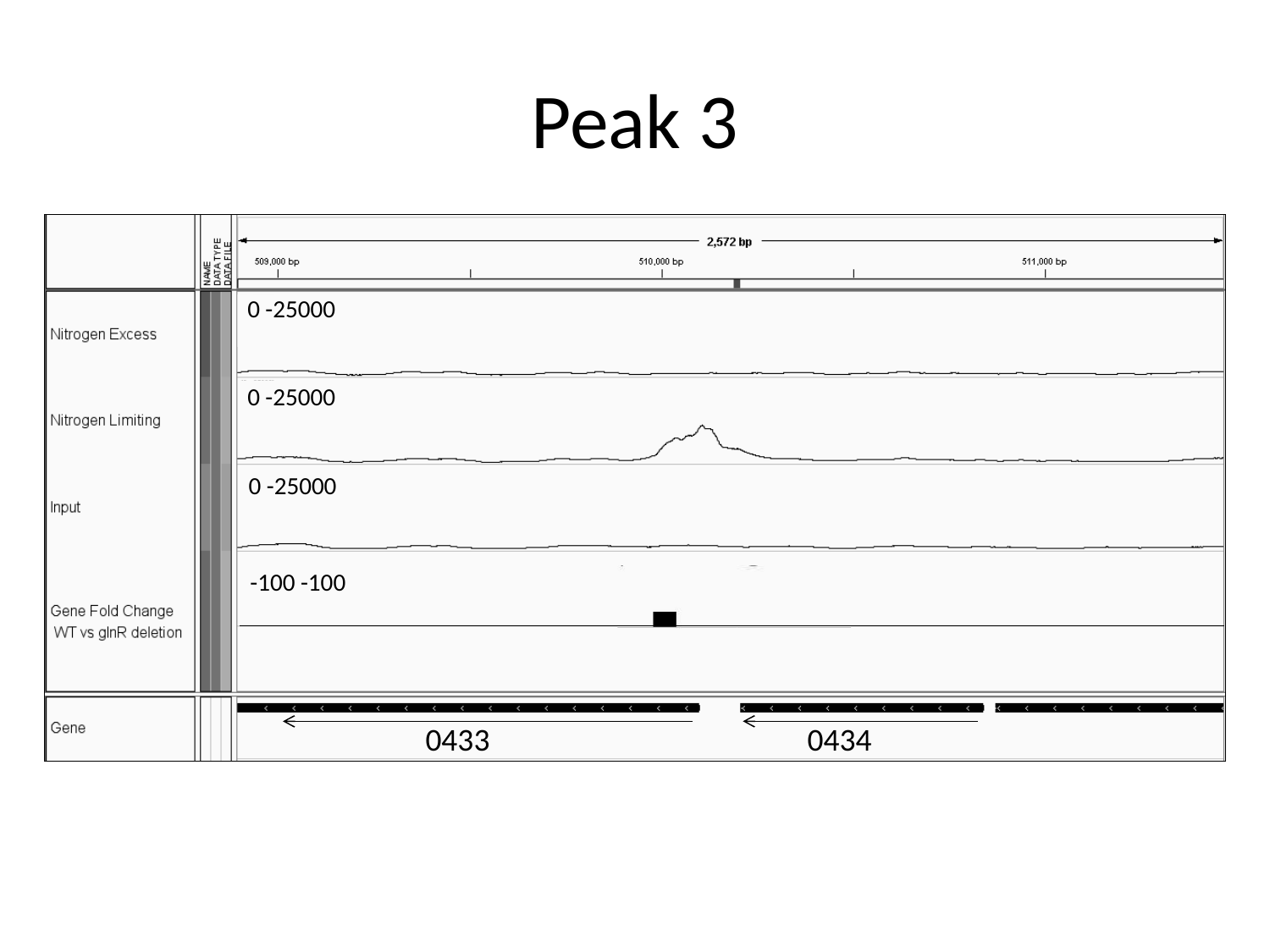

# Peak 3
0 -25000
0 -25000
0 -25000
-100 -100
0433
0434

## Slide 5
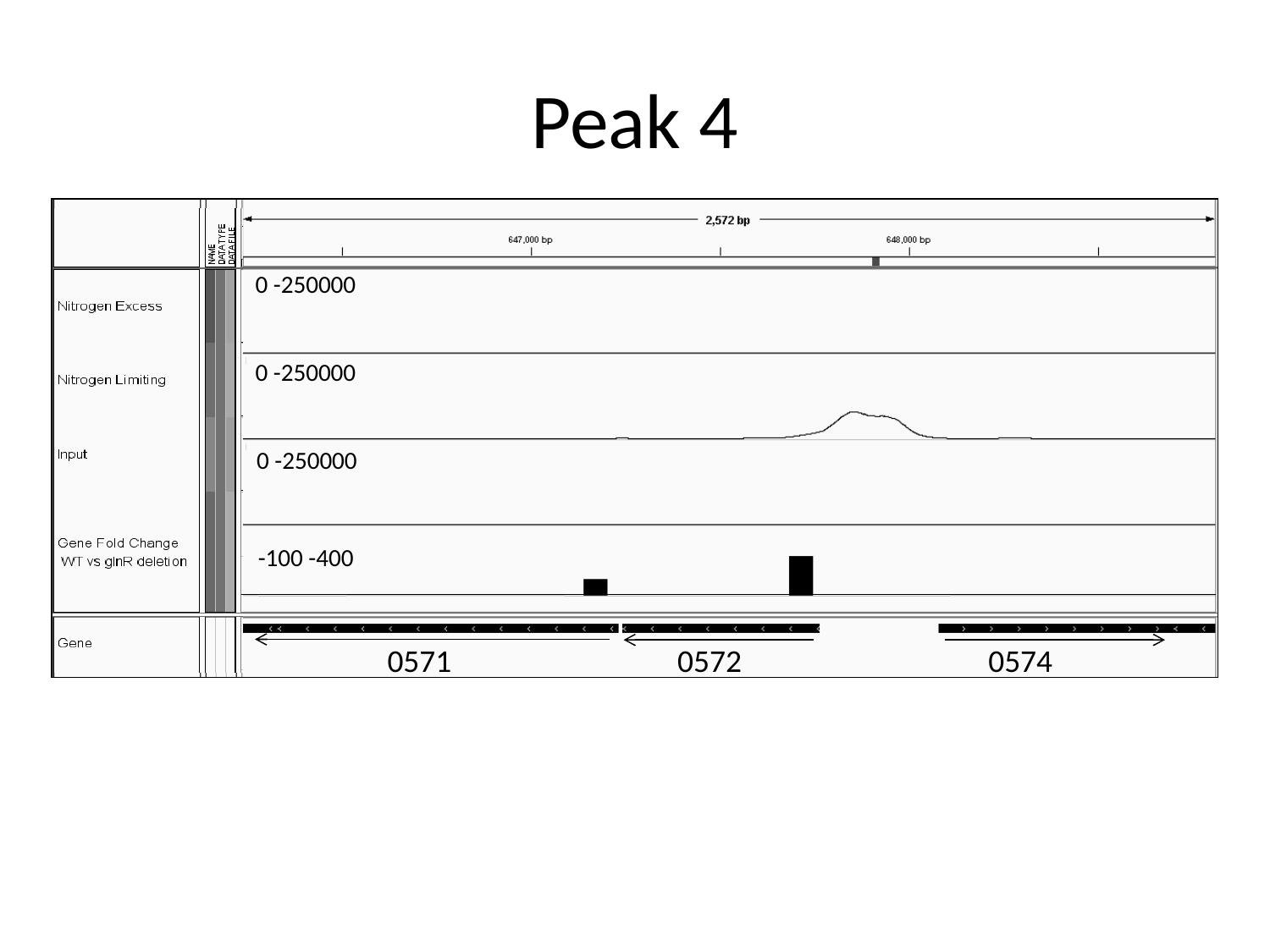

# Peak 4
0 -250000
0 -250000
0 -250000
-100 -400
0571
0572
0574

## Slide 6
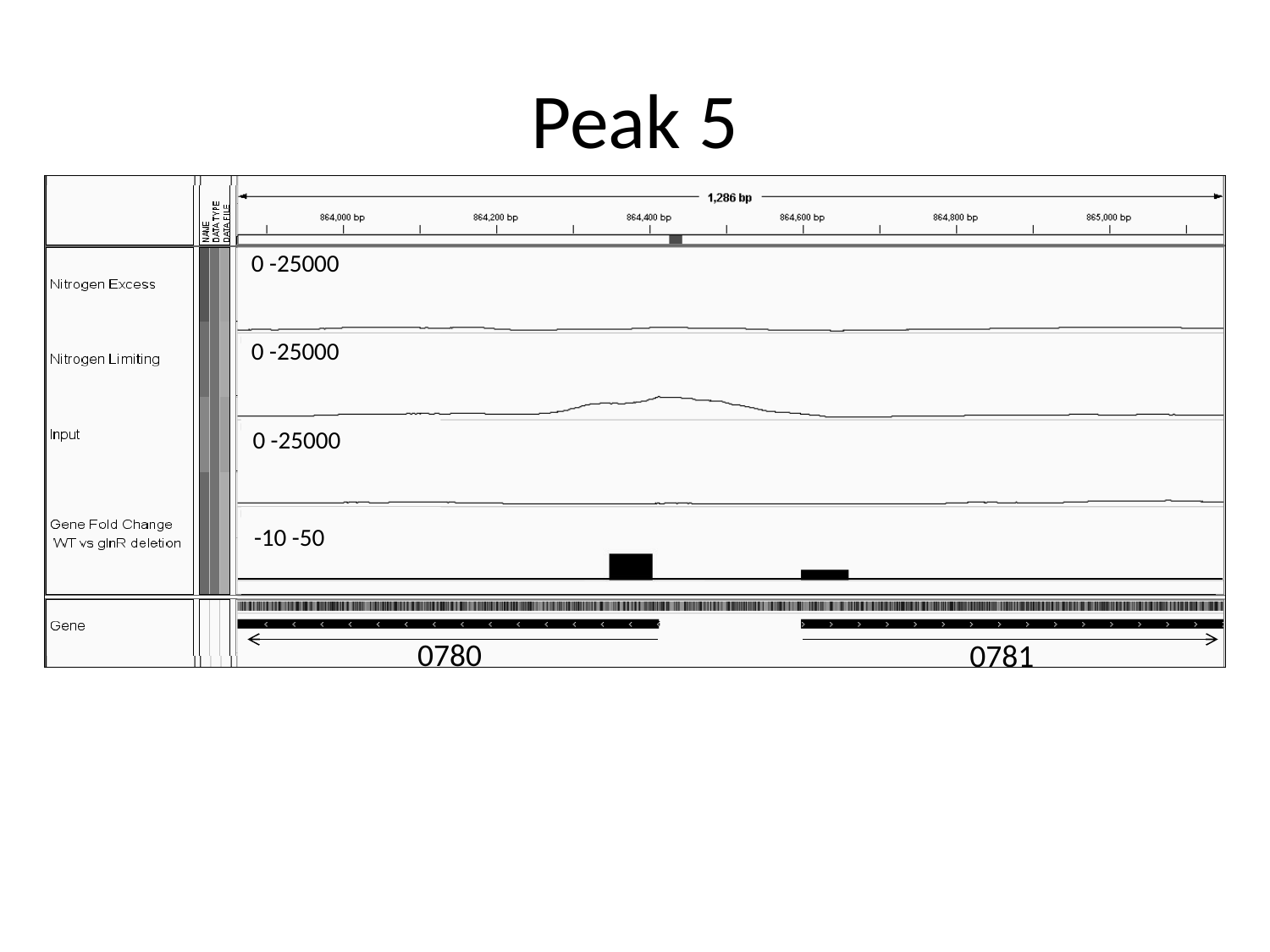

# Peak 5
0 -25000
0 -25000
0 -25000
-10 -50
0780
0781

## Slide 7
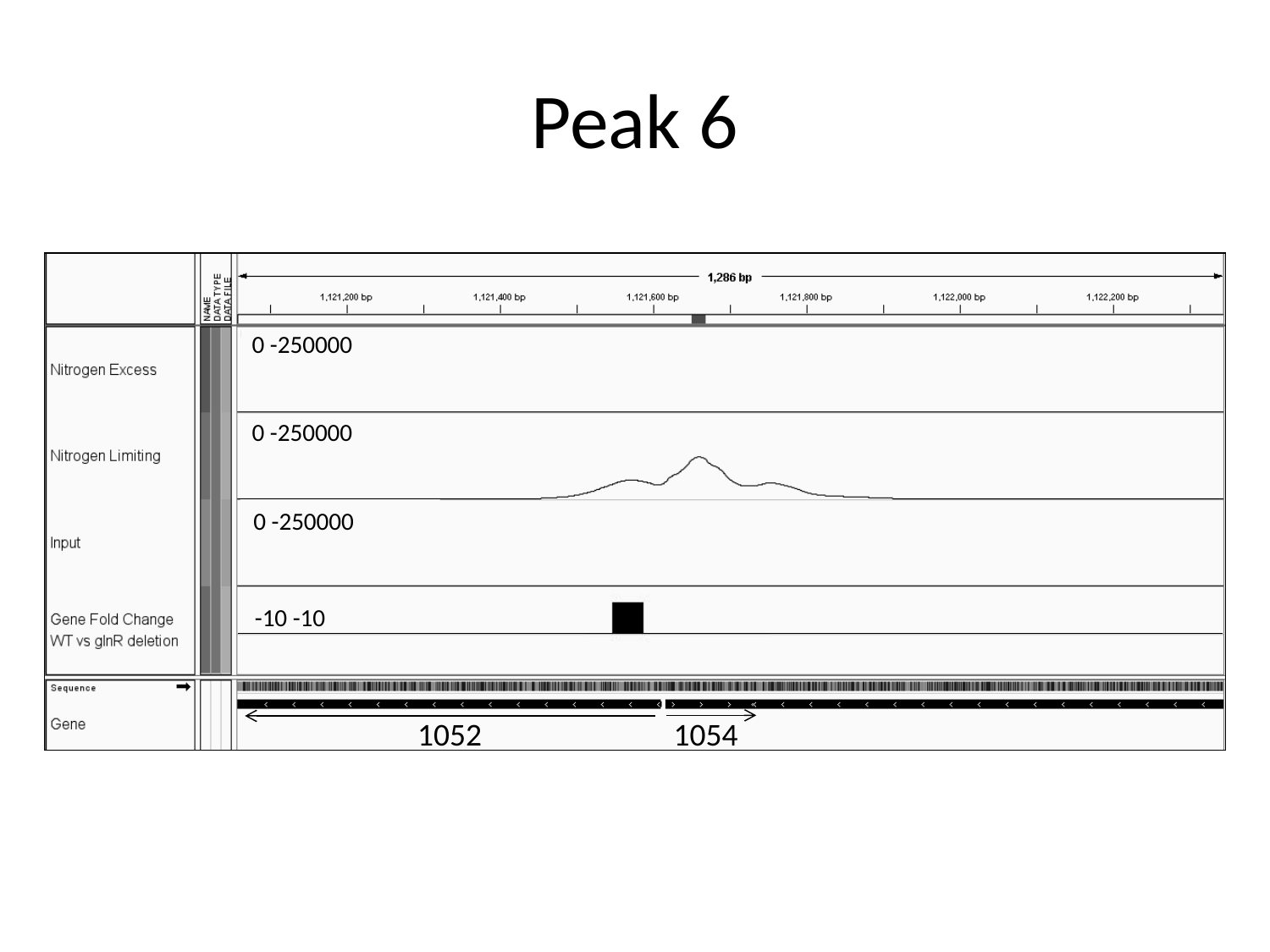

# Peak 6
0 -250000
0 -250000
0 -250000
-10 -10
1052
1054

## Slide 8
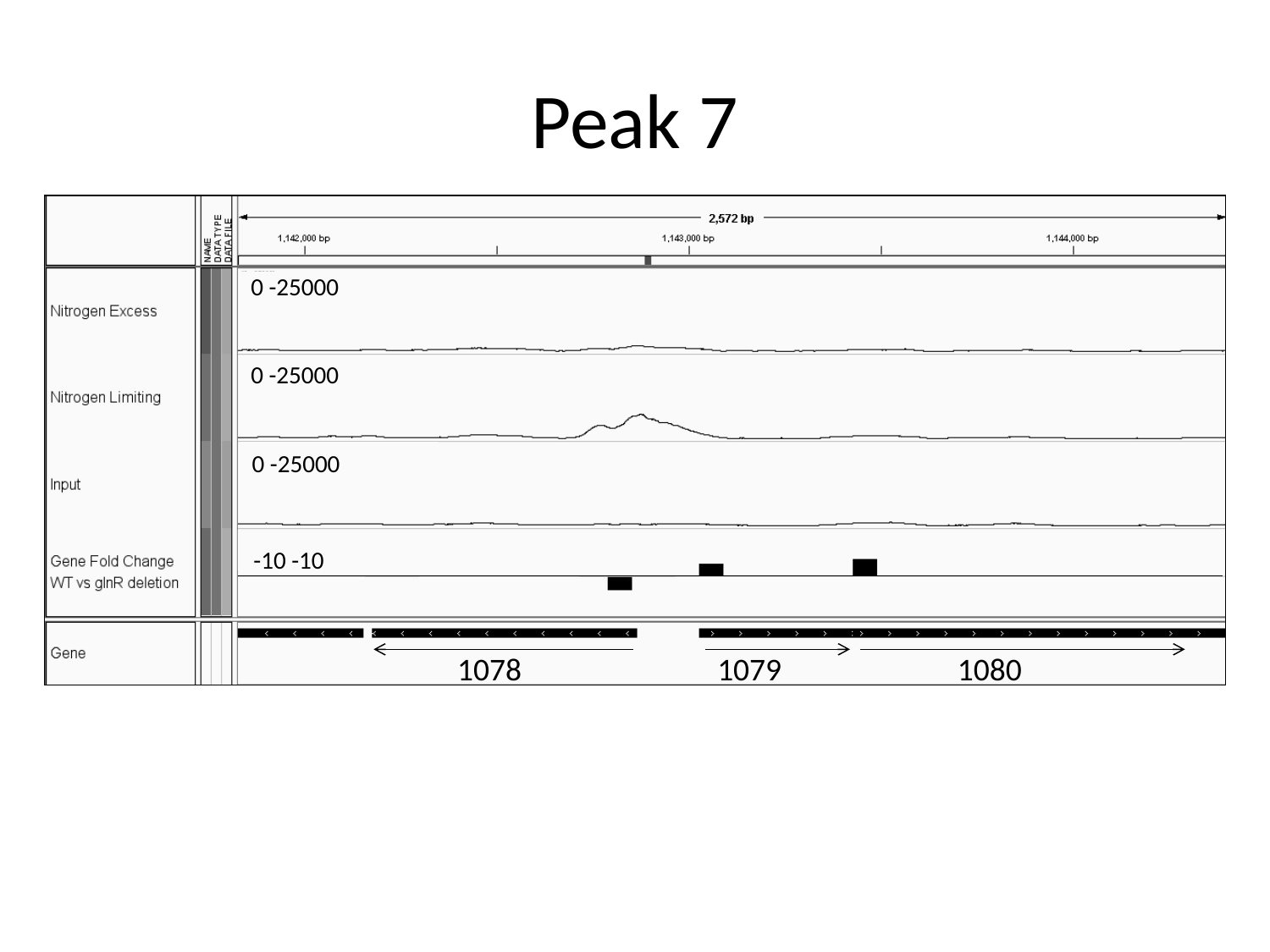

# Peak 7
0 -25000
0 -25000
0 -25000
-10 -10
1078
1079
1080

## Slide 9
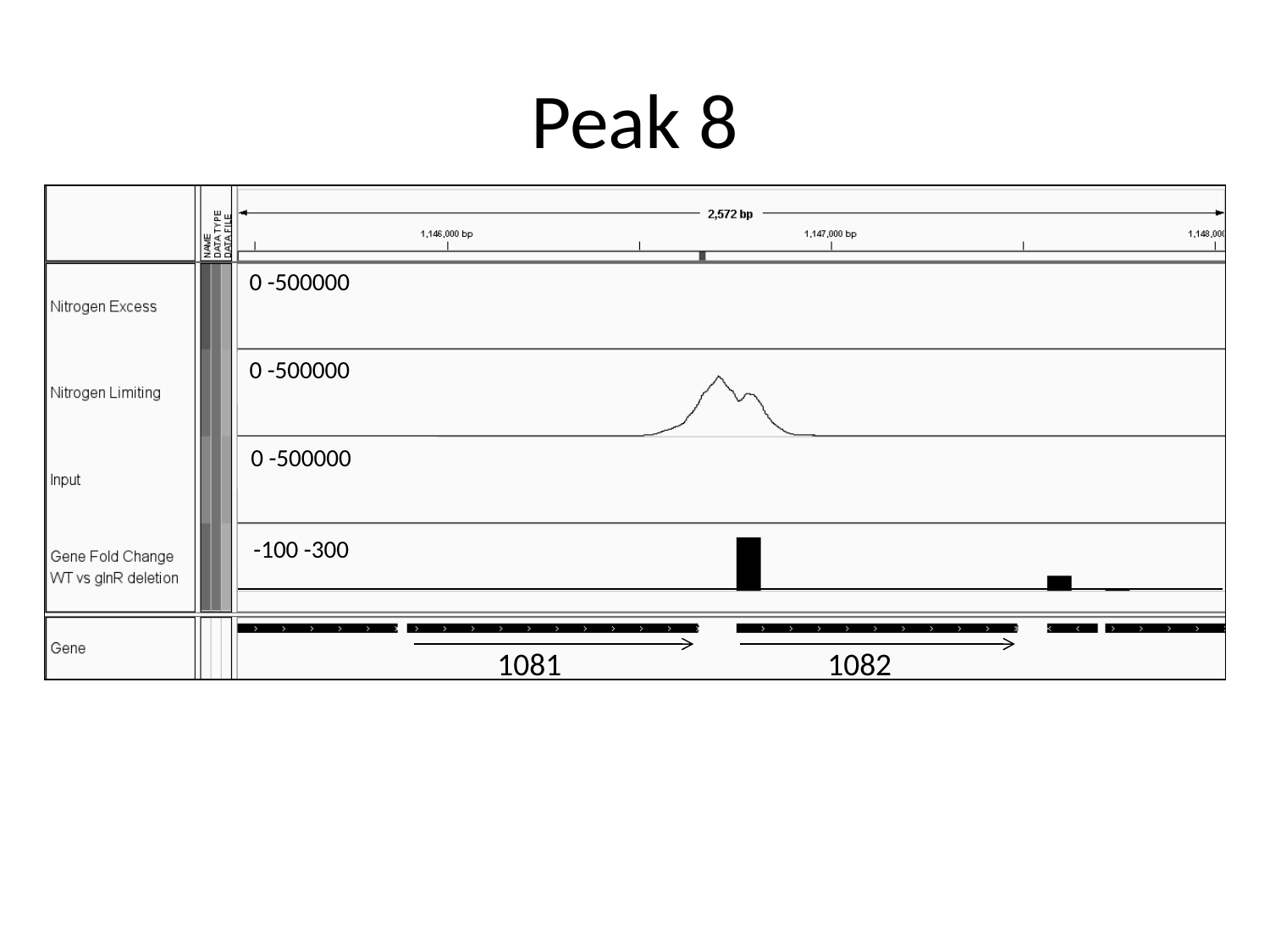

# Peak 8
0 -500000
0 -500000
0 -500000
-100 -300
1081
1082

## Slide 10
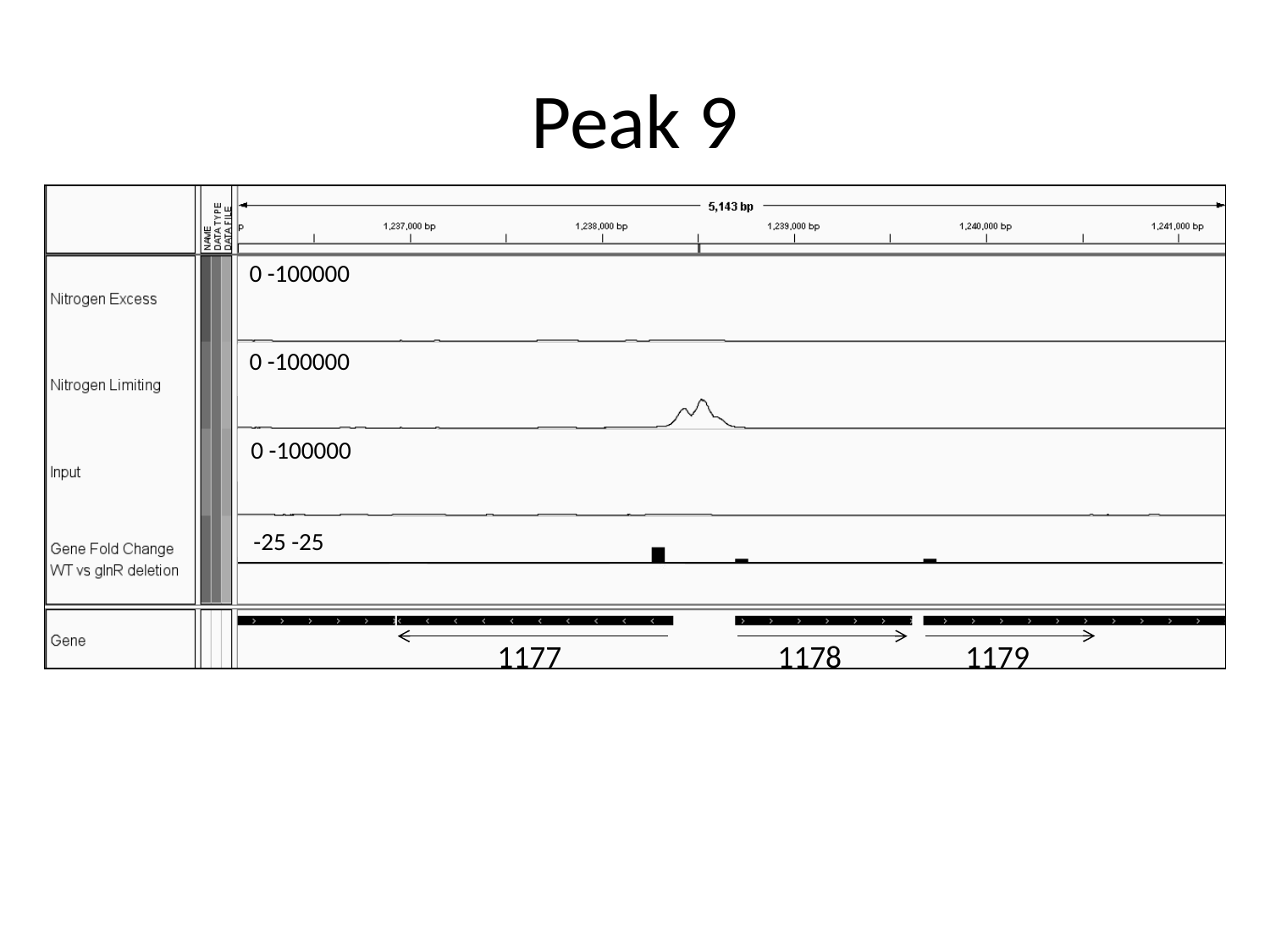

# Peak 9
0 -100000
0 -100000
0 -100000
-25 -25
1179
1177
1178

## Slide 11
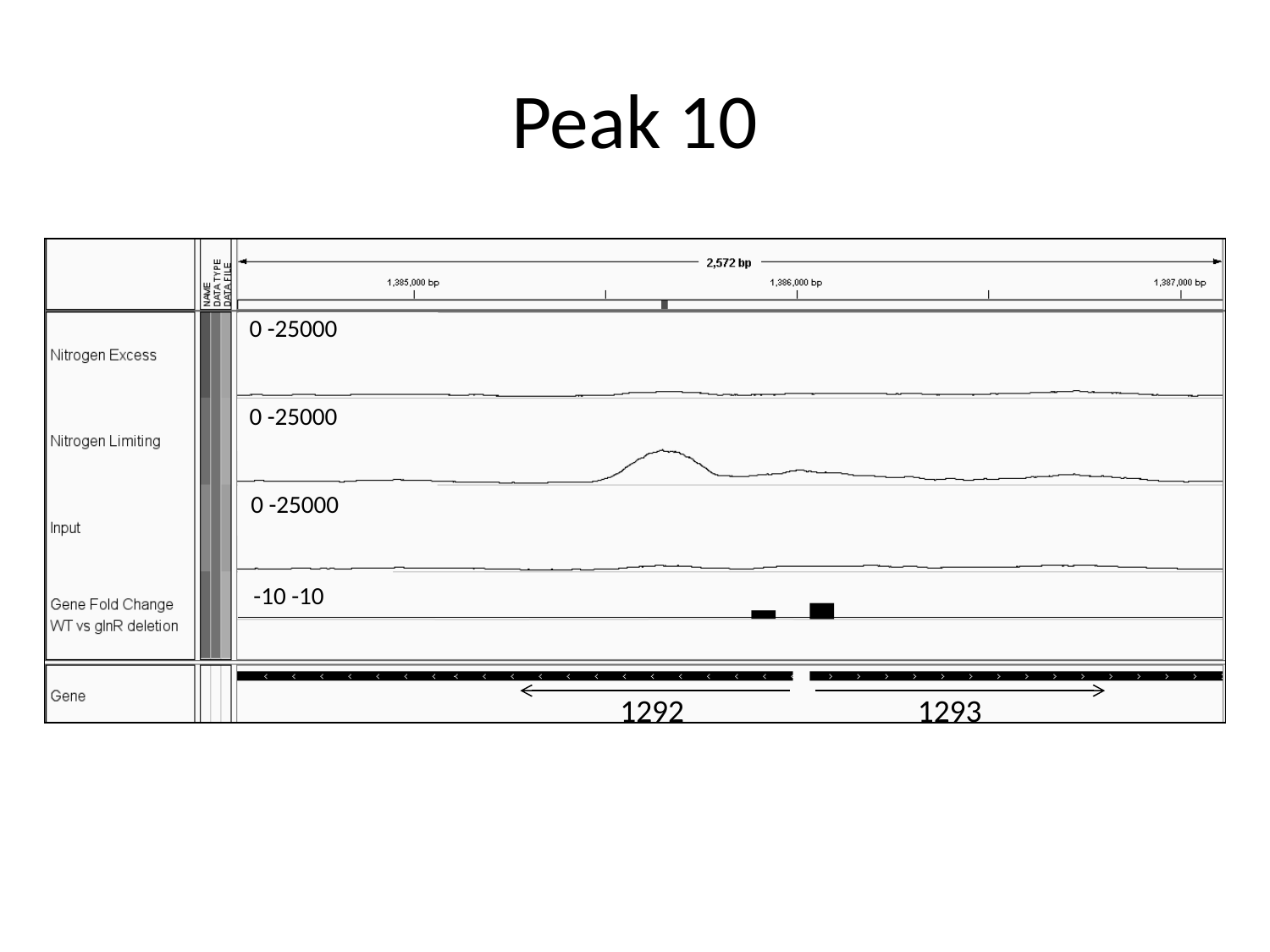

# Peak 10
0 -25000
0 -25000
0 -25000
-10 -10
1292
1293

## Slide 12
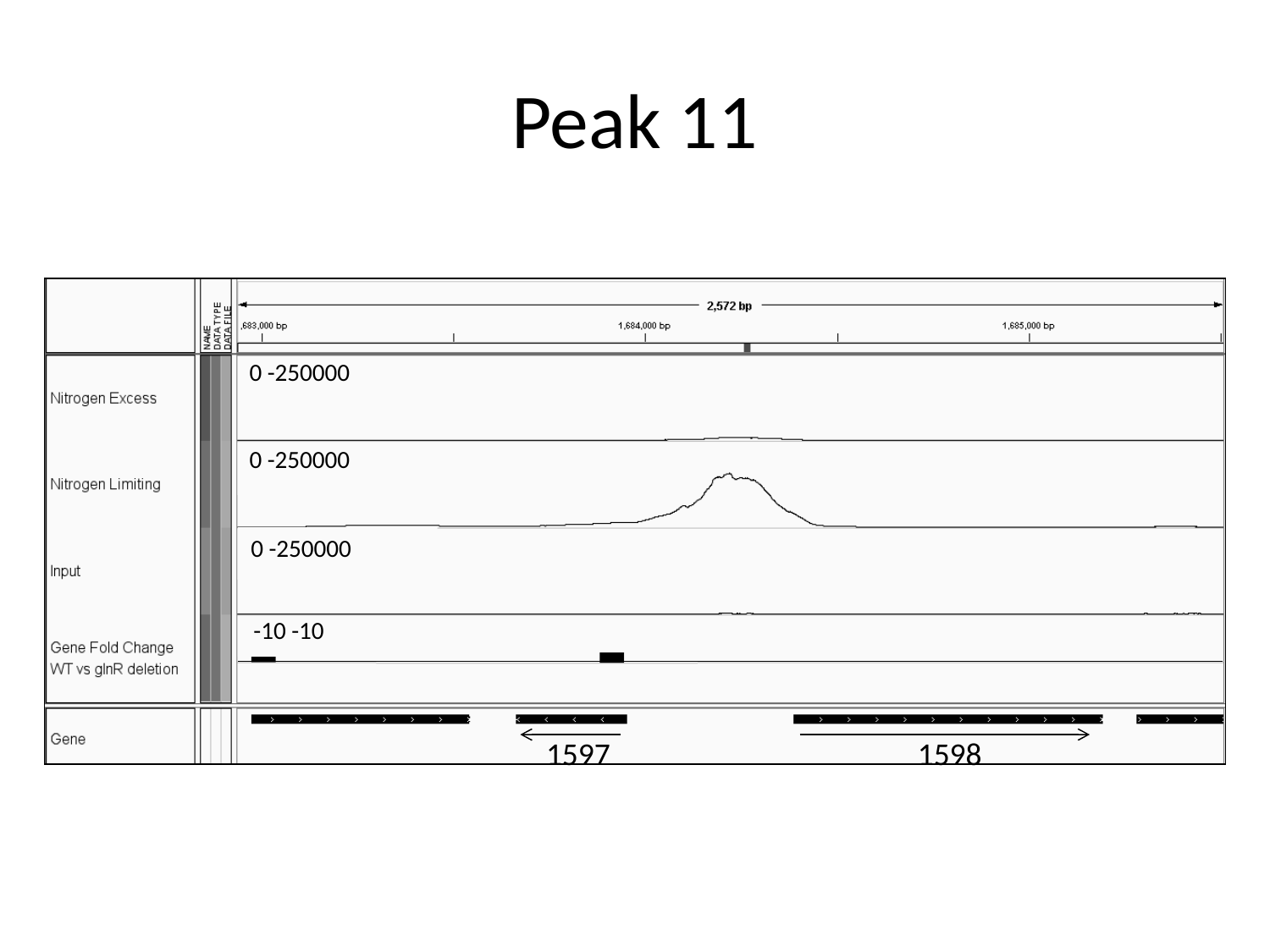

# Peak 11
0 -250000
0 -250000
0 -250000
-10 -10
1597
1598

## Slide 13
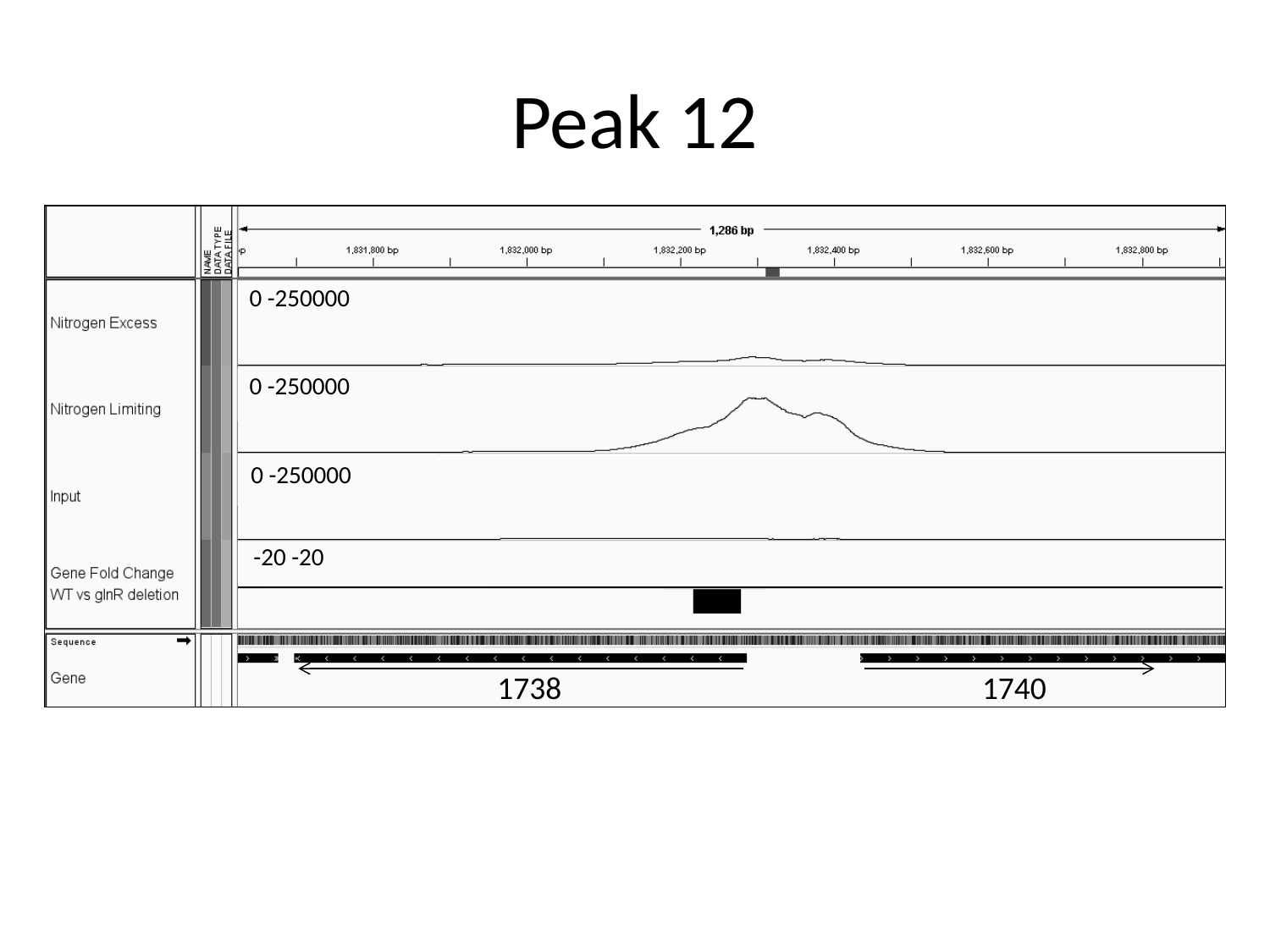

# Peak 12
0 -250000
0 -250000
0 -250000
-20 -20
1738
1740

## Slide 14
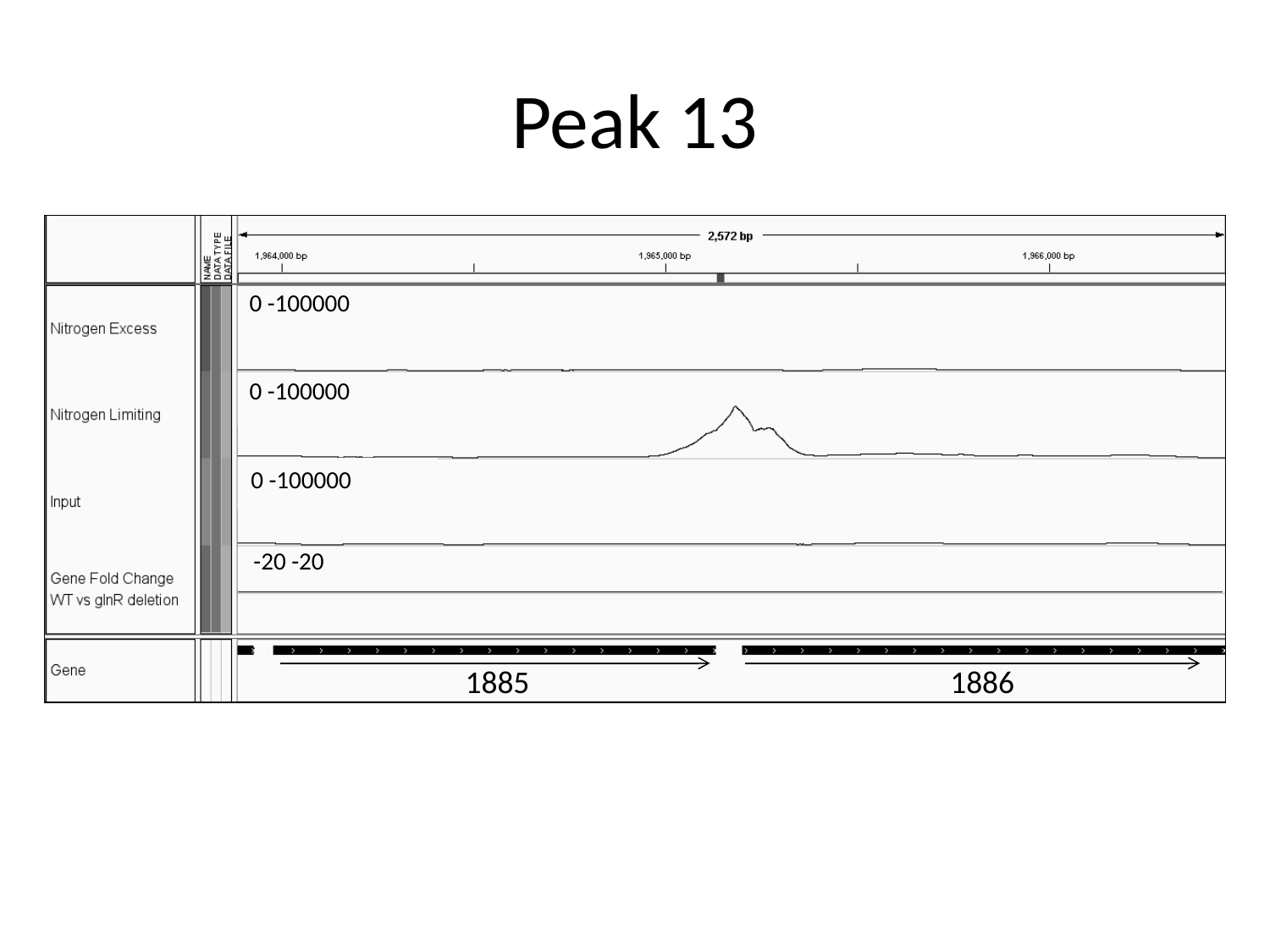

# Peak 13
0 -100000
0 -100000
0 -100000
-20 -20
1885
1886

## Slide 15
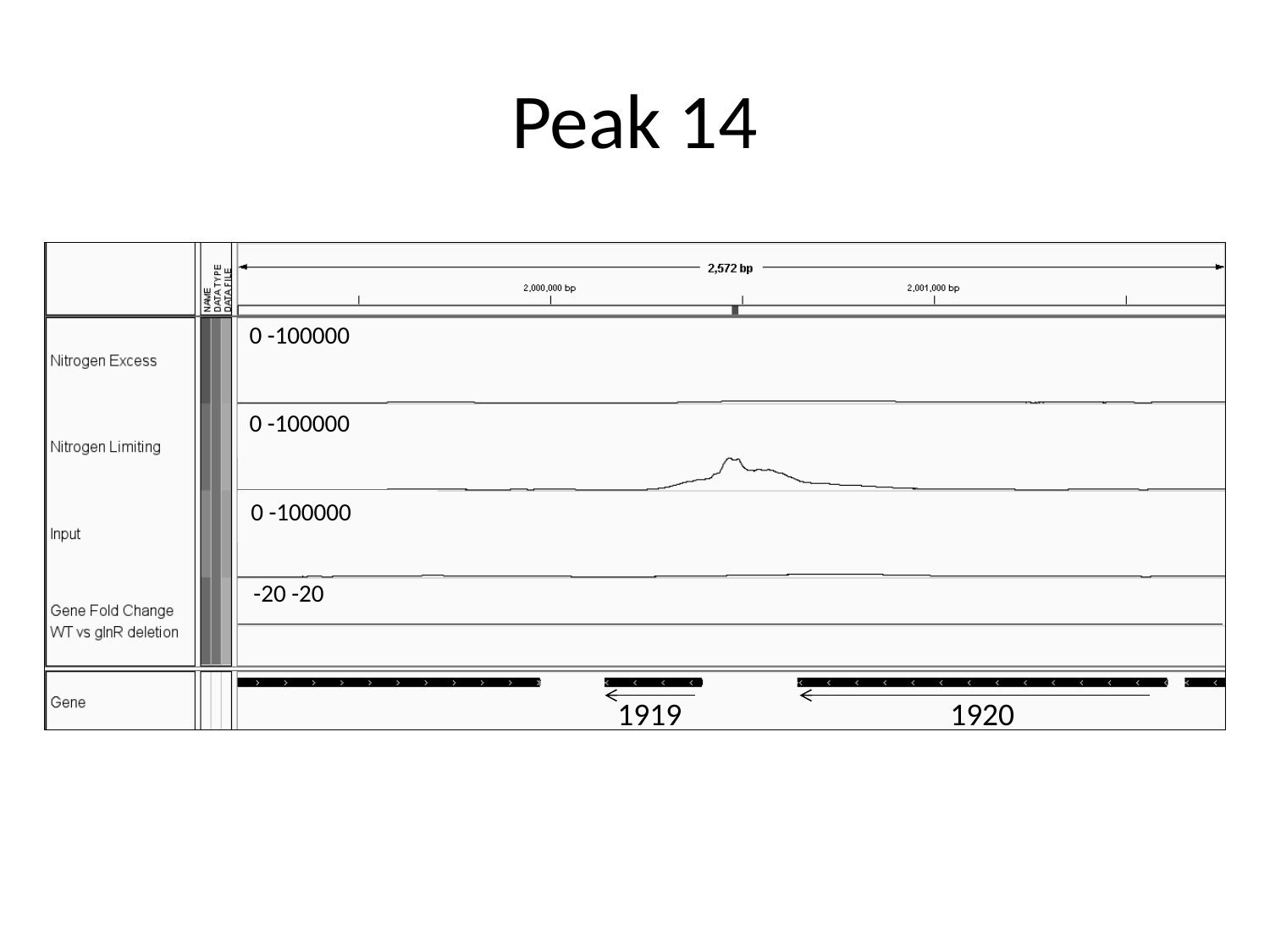

# Peak 14
0 -100000
0 -100000
0 -100000
-20 -20
1919
1920

## Slide 16
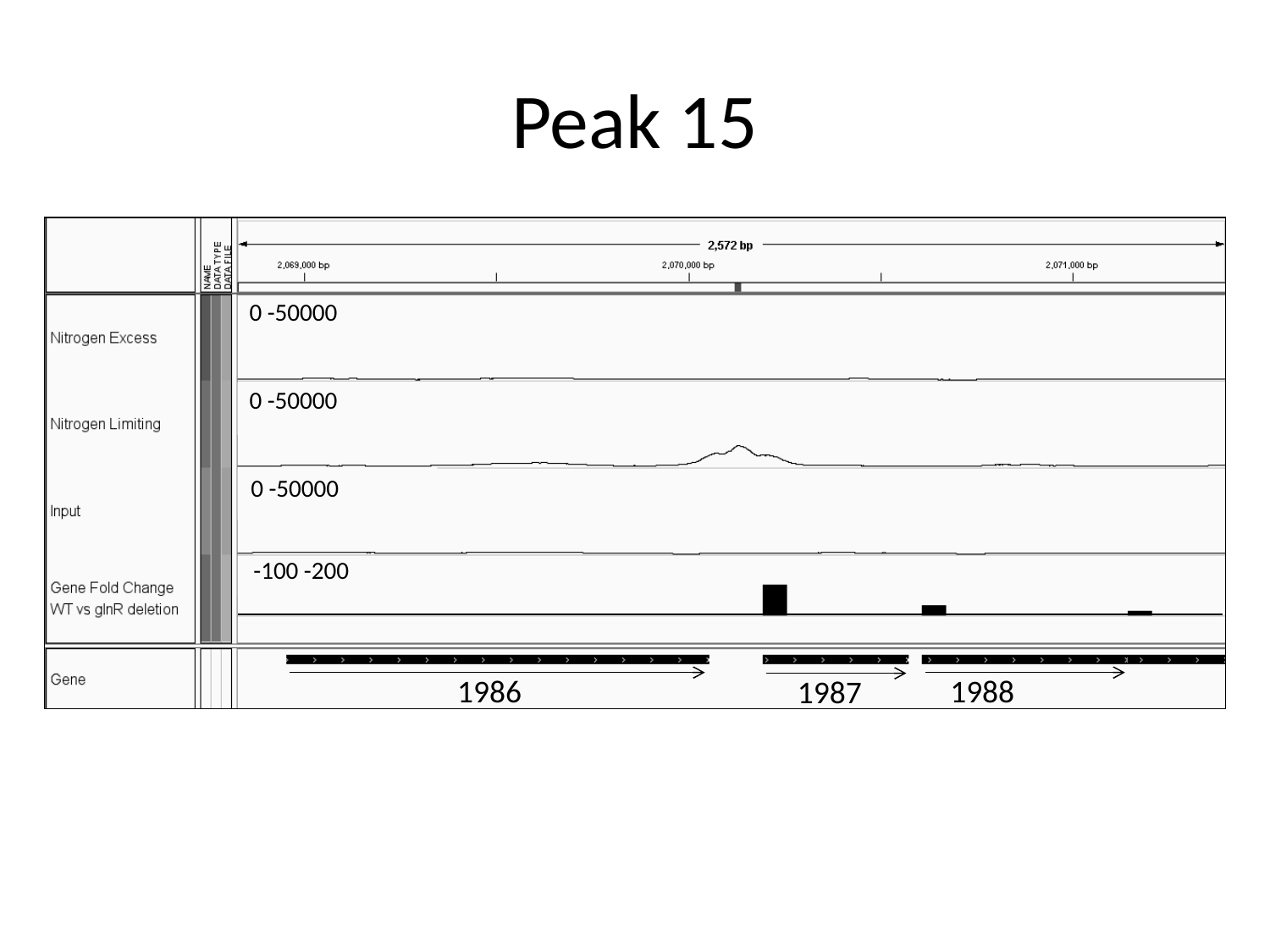

# Peak 15
0 -50000
0 -50000
0 -50000
-100 -200
1986
1988
1987

## Slide 17
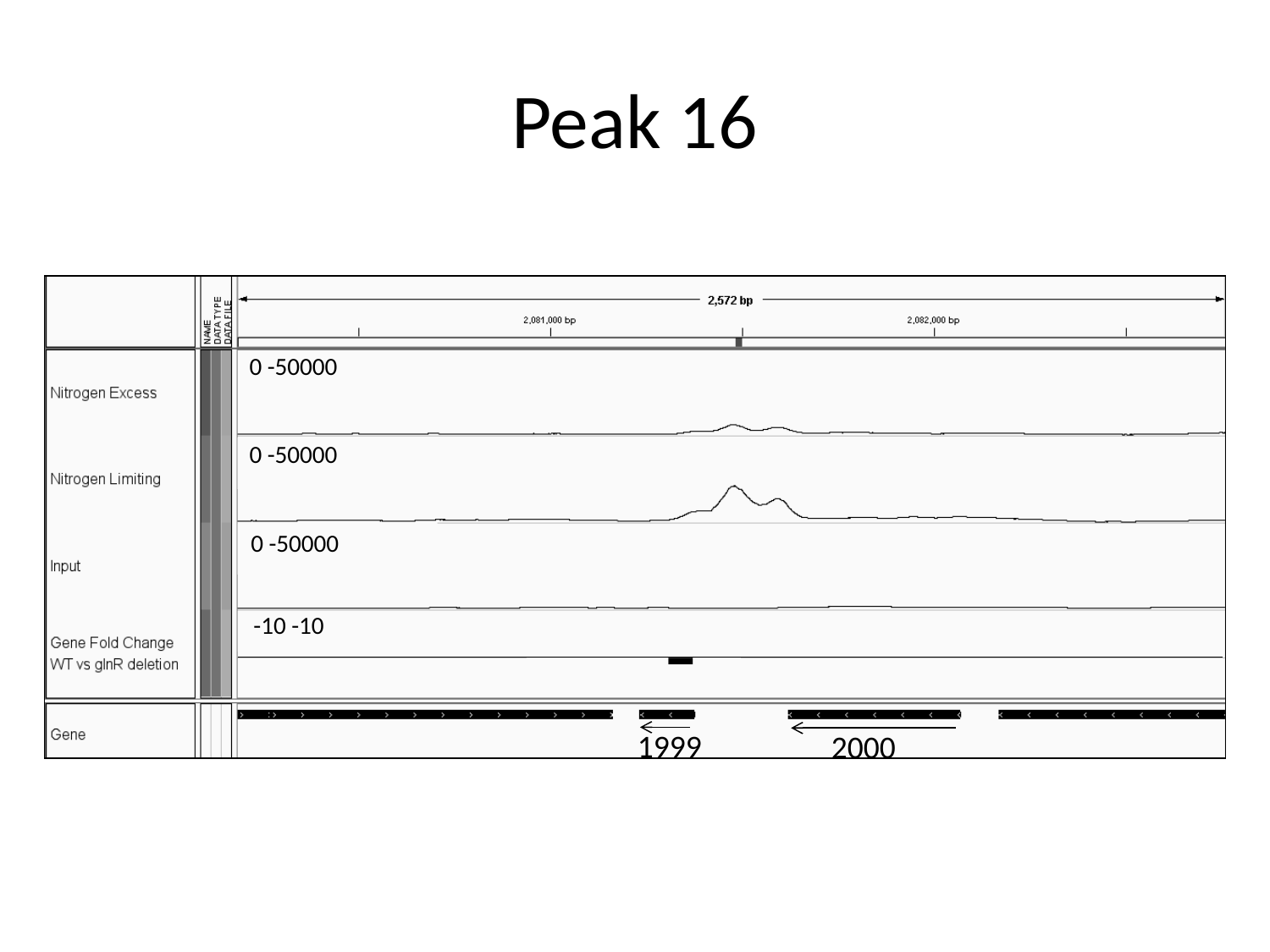

# Peak 16
0 -50000
0 -50000
0 -50000
-10 -10
1999
2000

## Slide 18
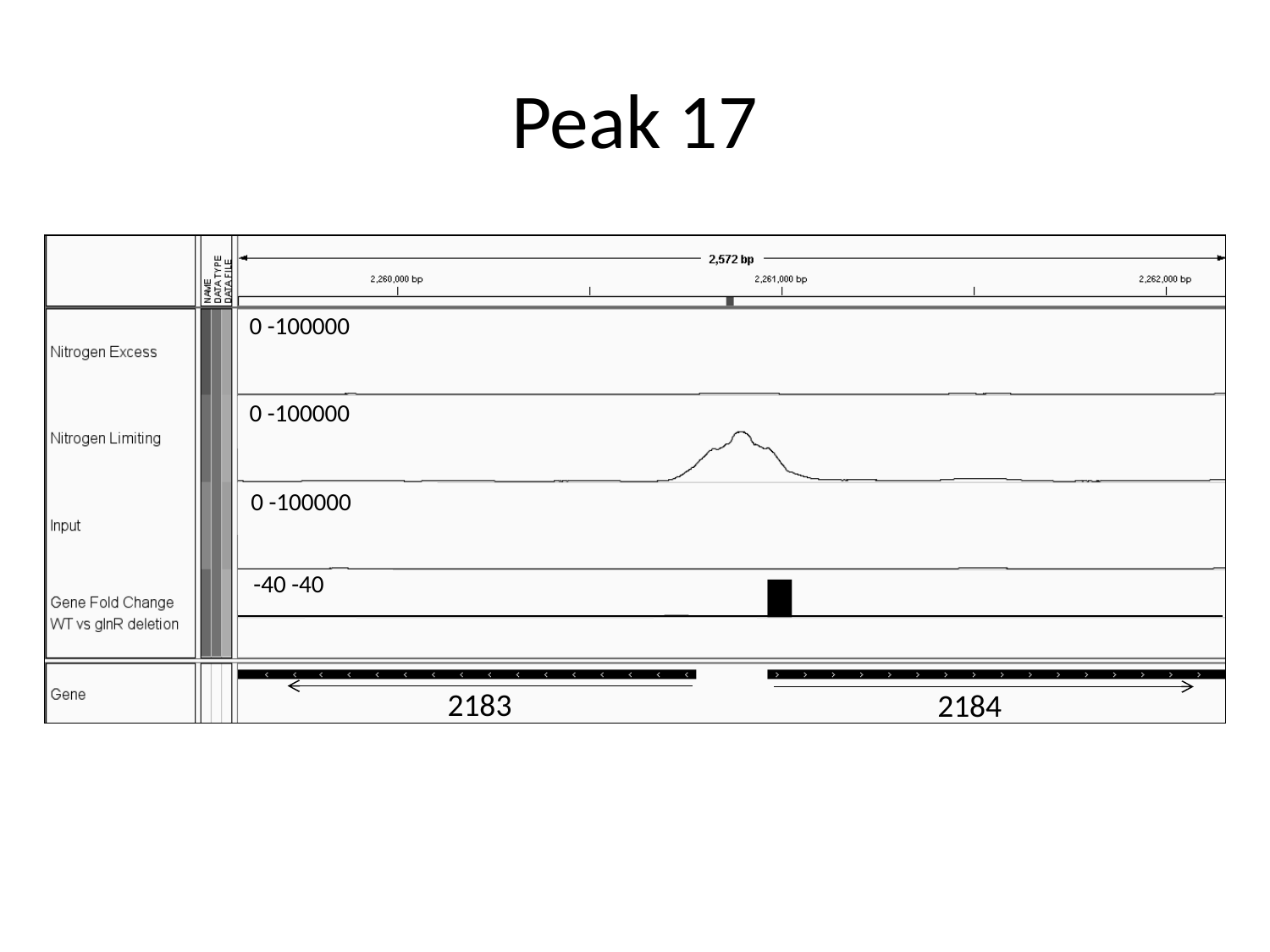

# Peak 17
0 -100000
0 -100000
0 -100000
-40 -40
2183
2184

## Slide 19
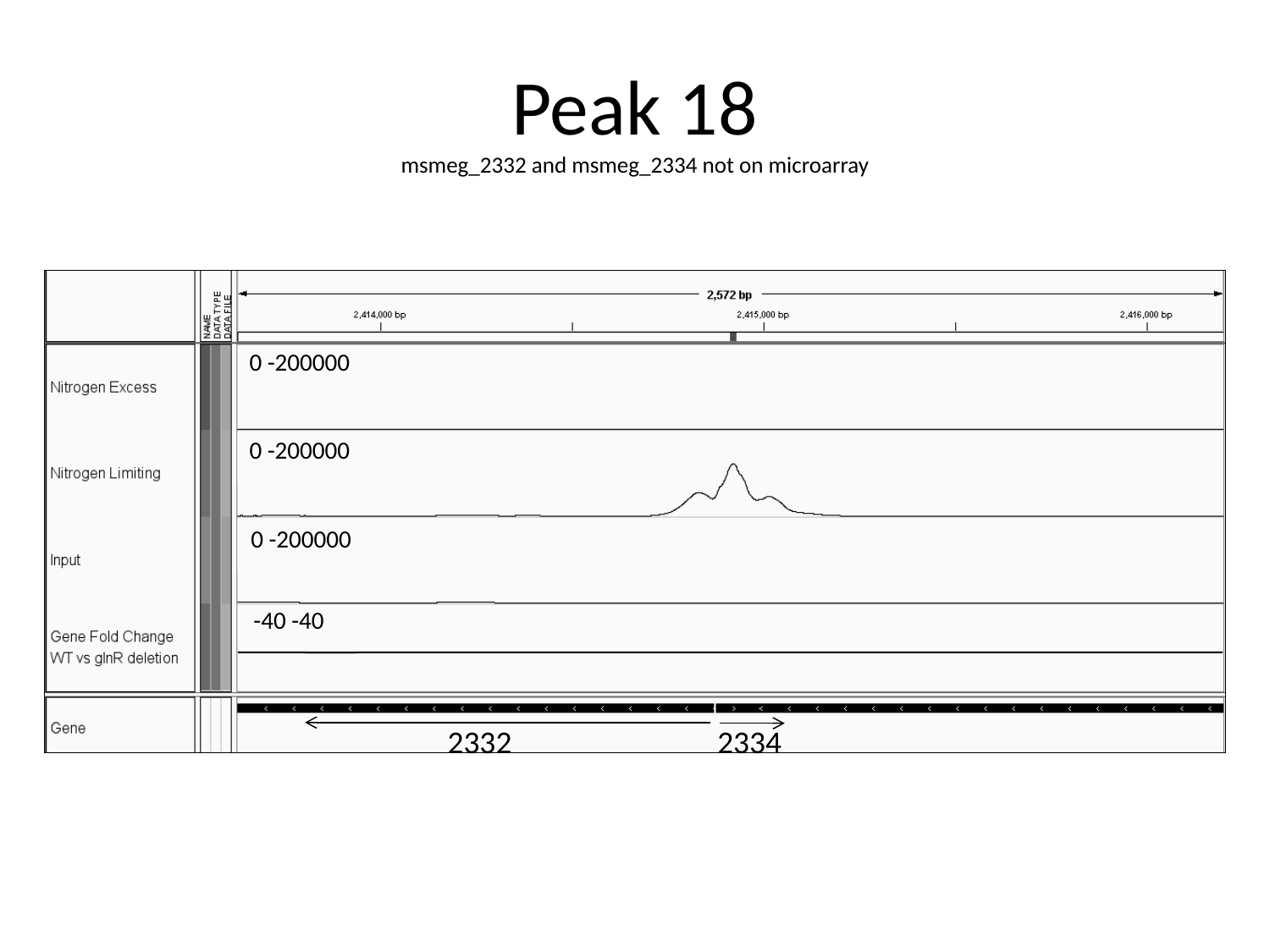

# Peak 18 msmeg_2332 and msmeg_2334 not on microarray
0 -200000
0 -200000
0 -200000
-40 -40
2332
2334

## Slide 20
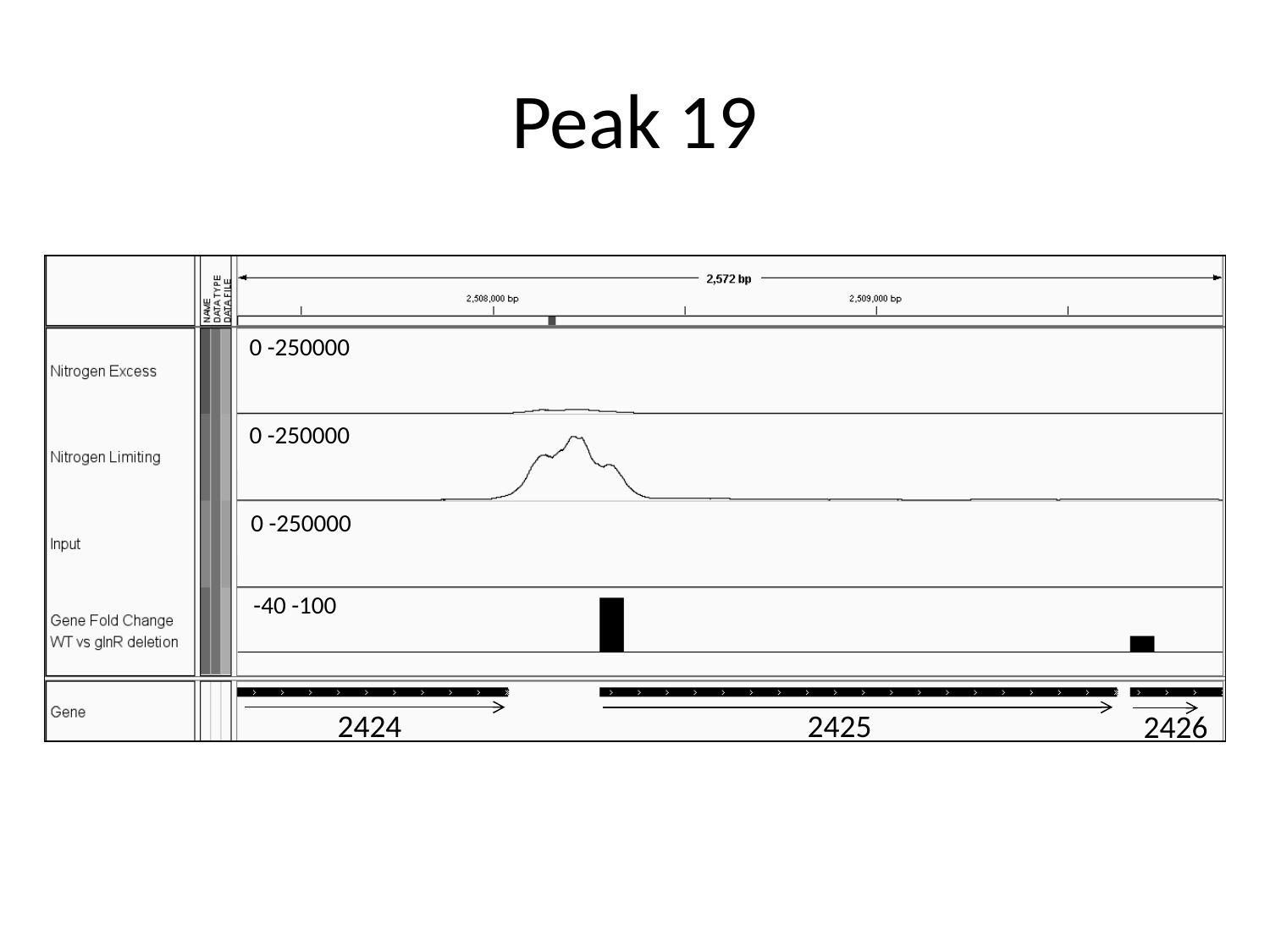

# Peak 19
0 -250000
0 -250000
0 -250000
-40 -100
2424
2425
2426

## Slide 21
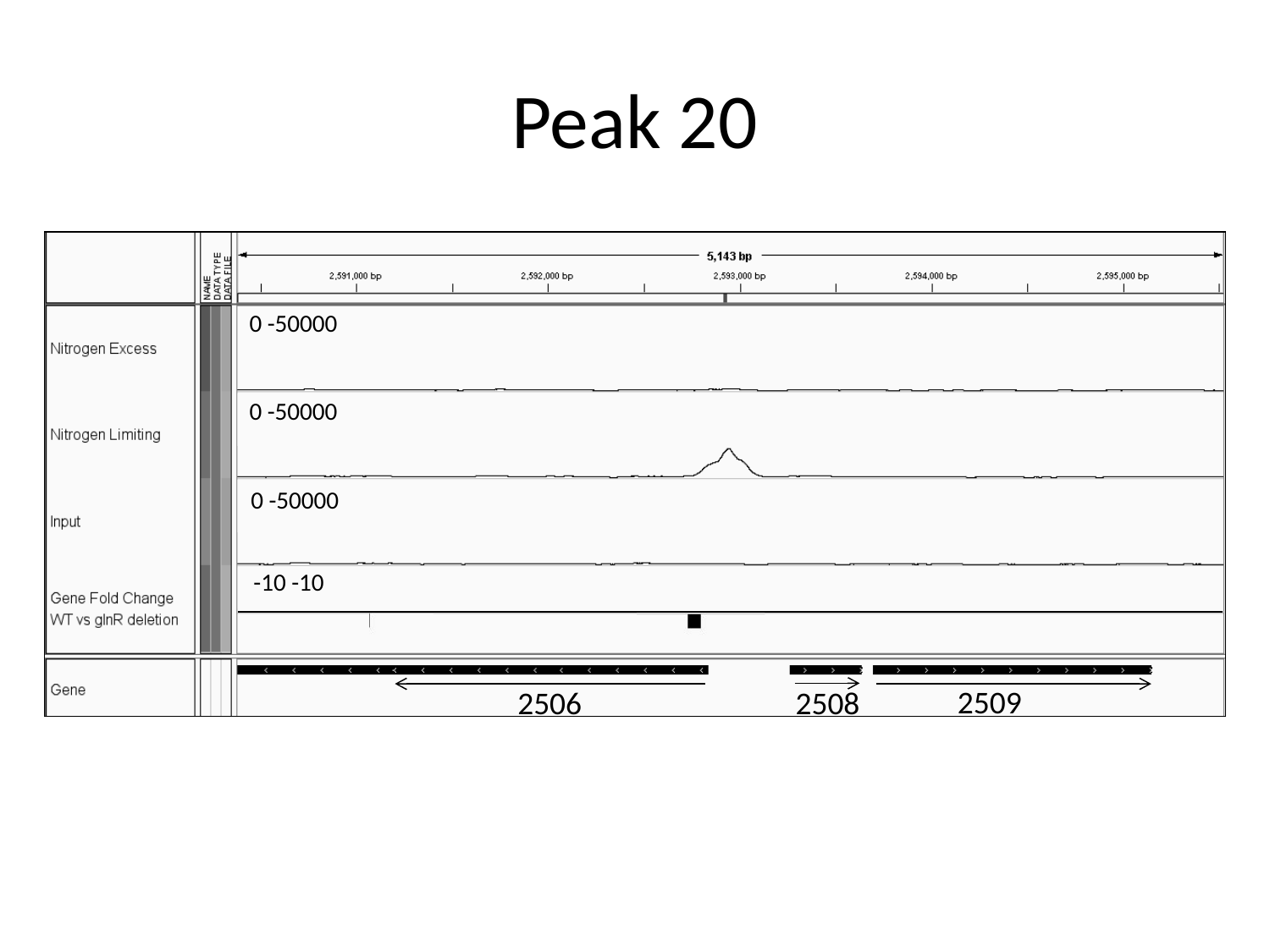

# Peak 20
0 -50000
0 -50000
0 -50000
-10 -10
2509
2506
2508

## Slide 22
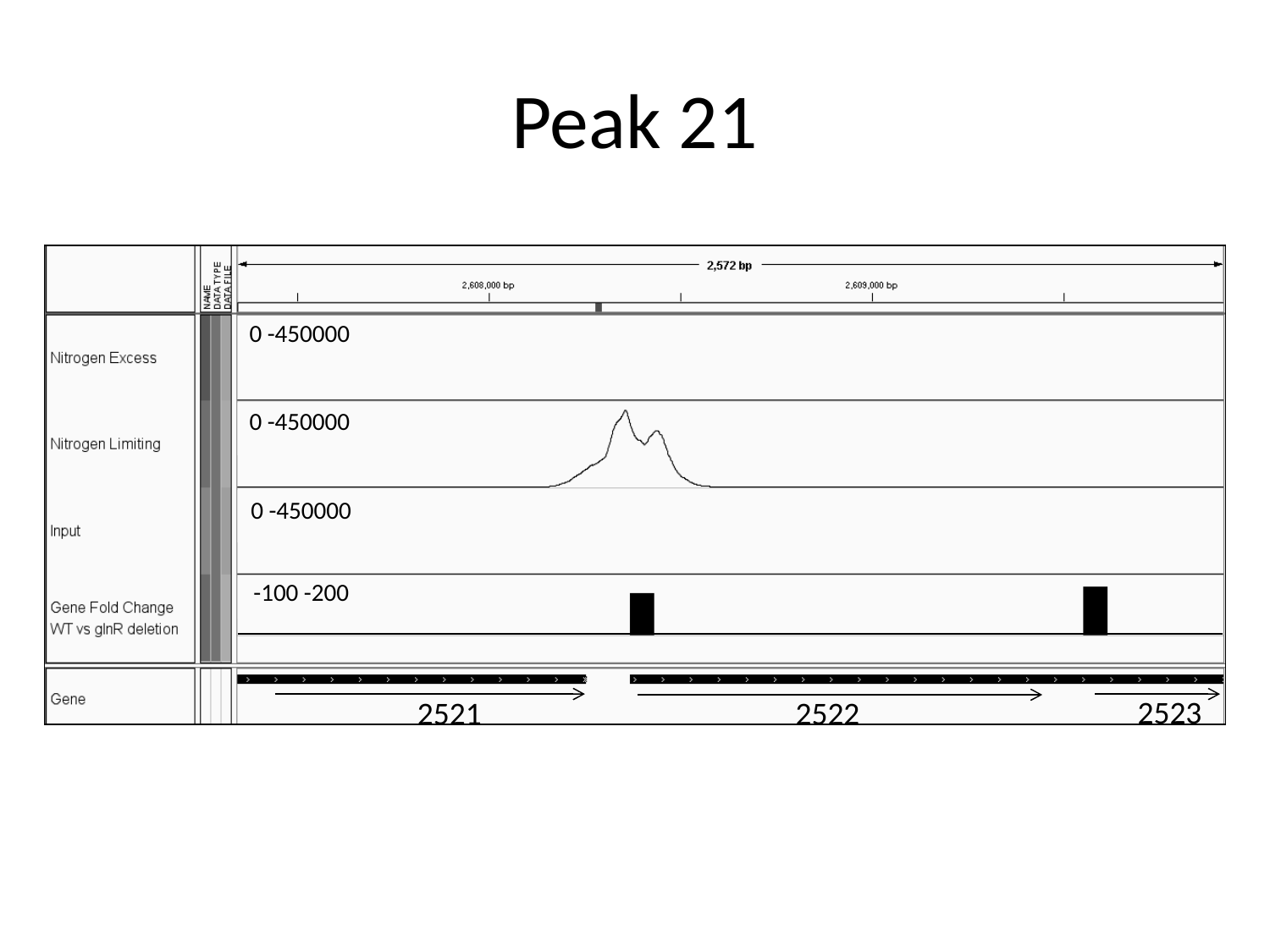

# Peak 21
0 -450000
0 -450000
0 -450000
-100 -200
2523
2521
2522

## Slide 23
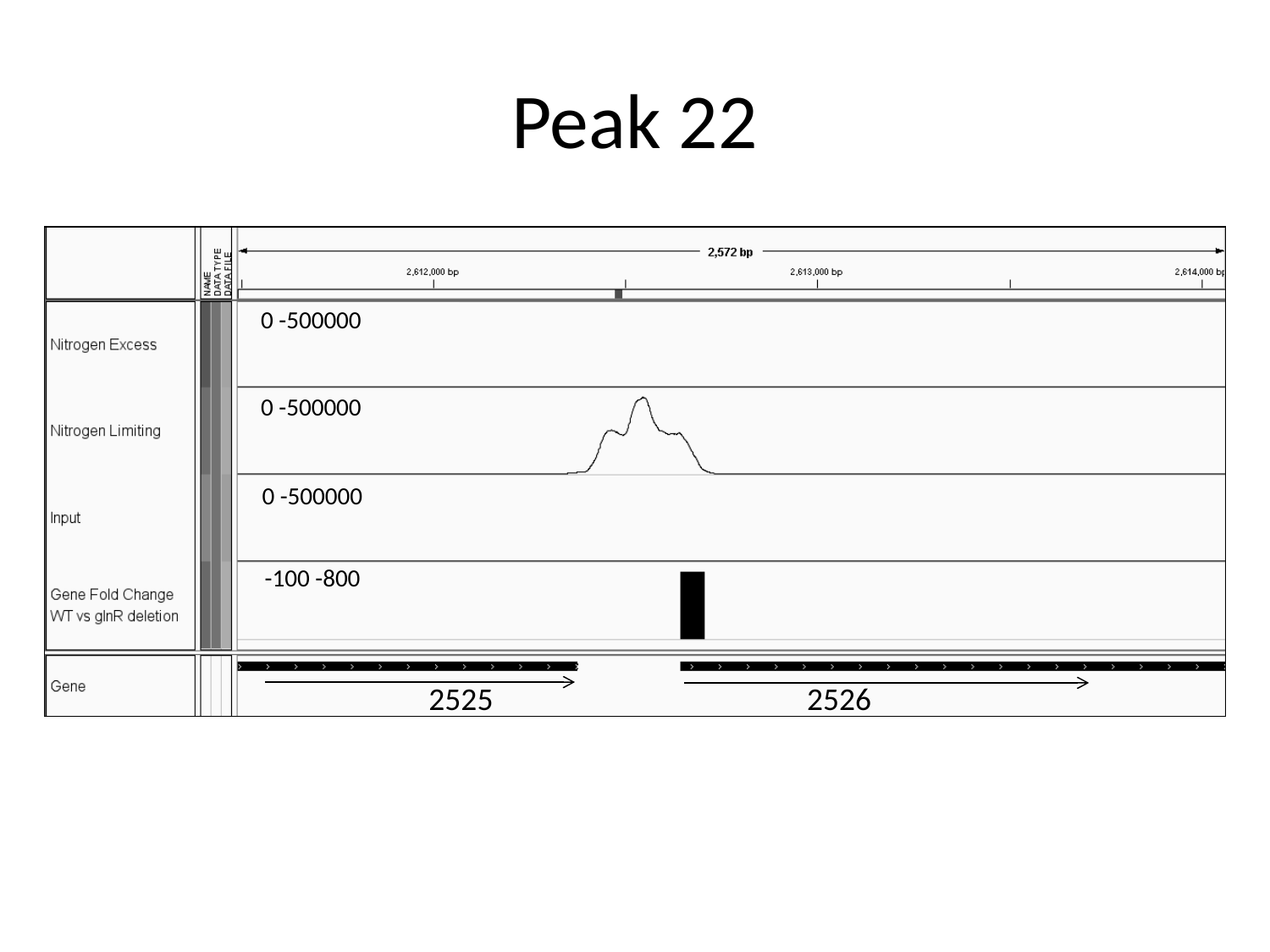

# Peak 22
0 -500000
0 -500000
0 -500000
-100 -800
2525
2526

## Slide 24
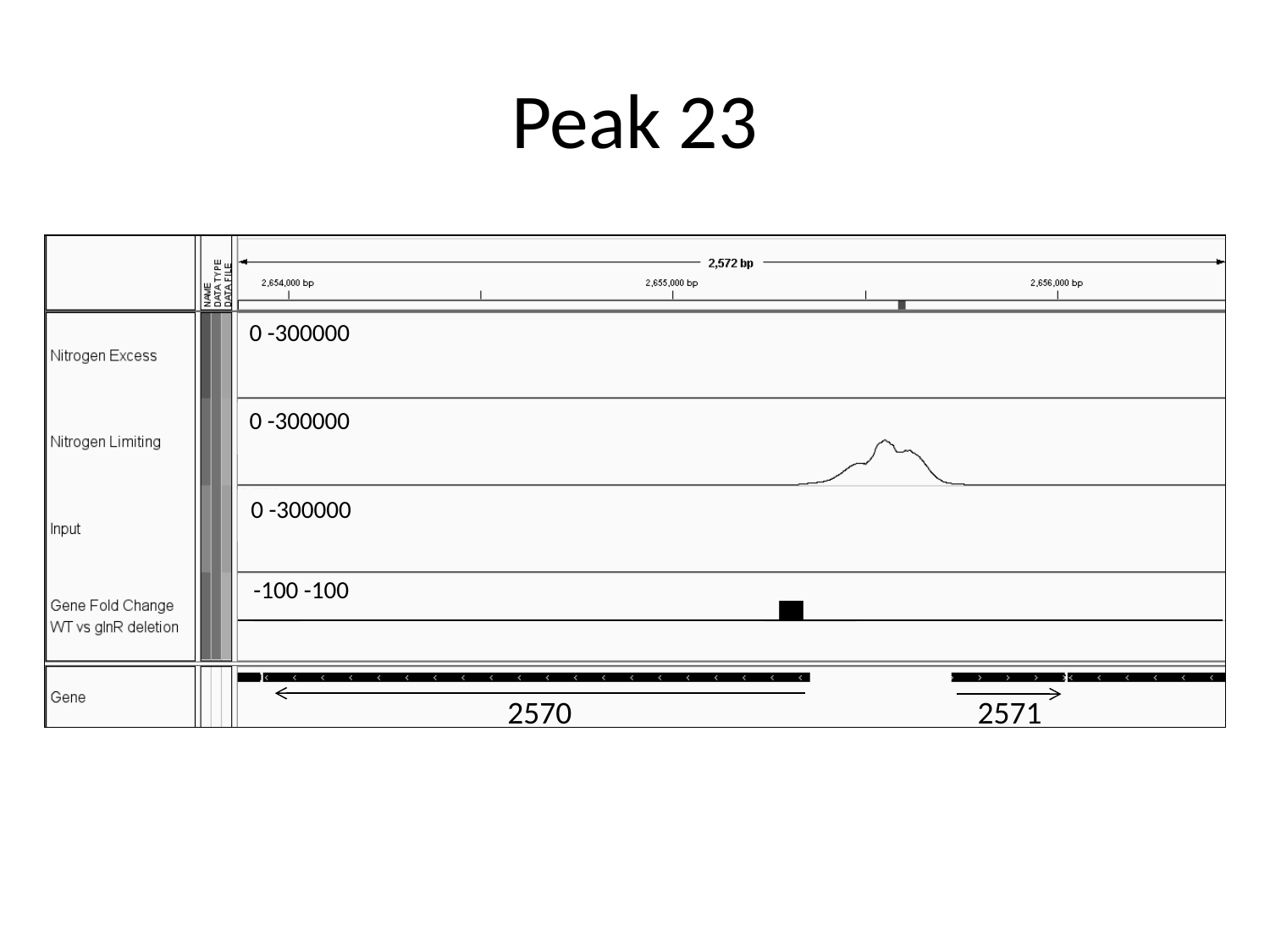

# Peak 23
0 -300000
0 -300000
0 -300000
-100 -100
2570
2571

## Slide 25
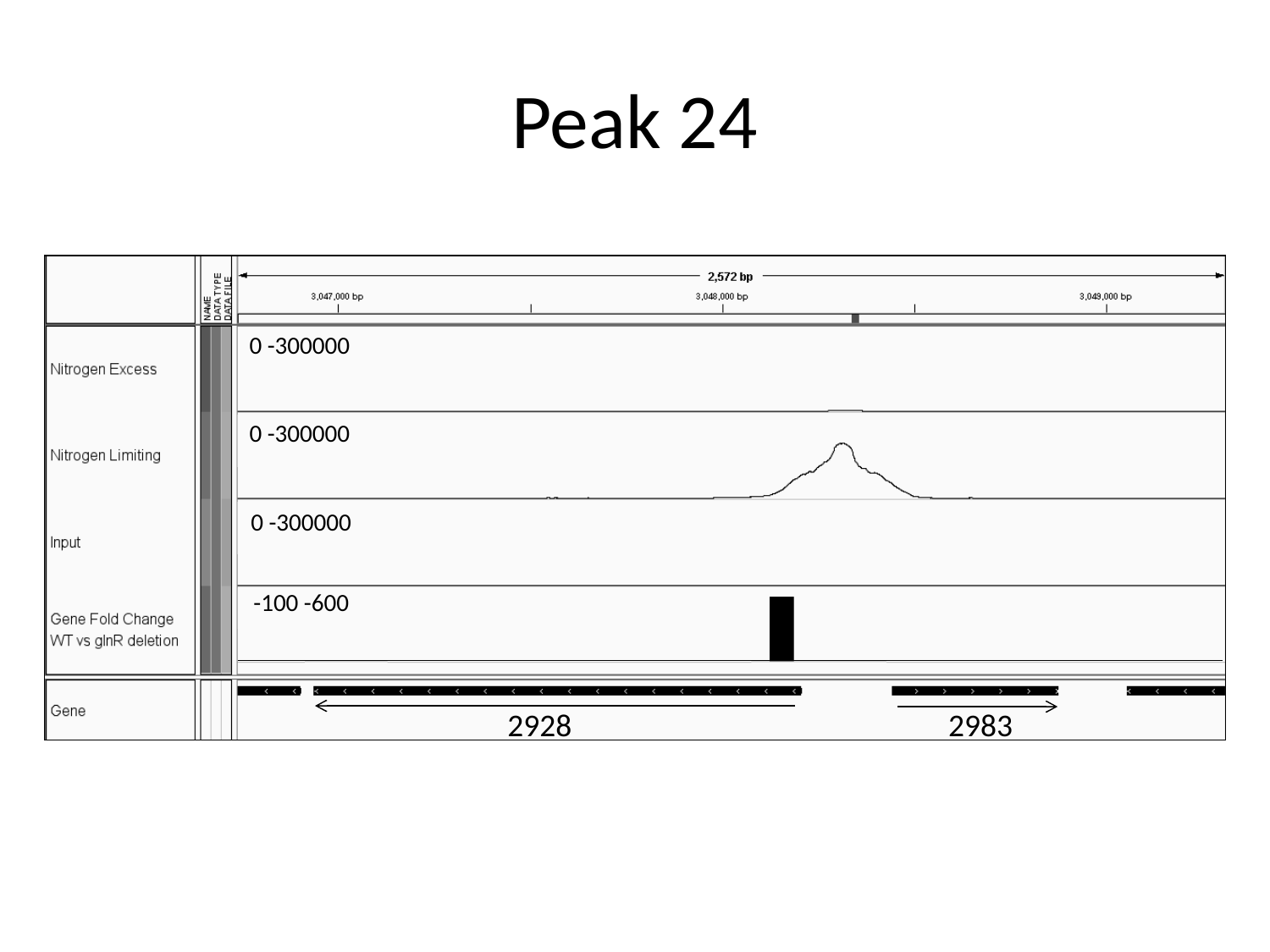

# Peak 24
0 -300000
0 -300000
0 -300000
-100 -600
2928
2983

## Slide 26
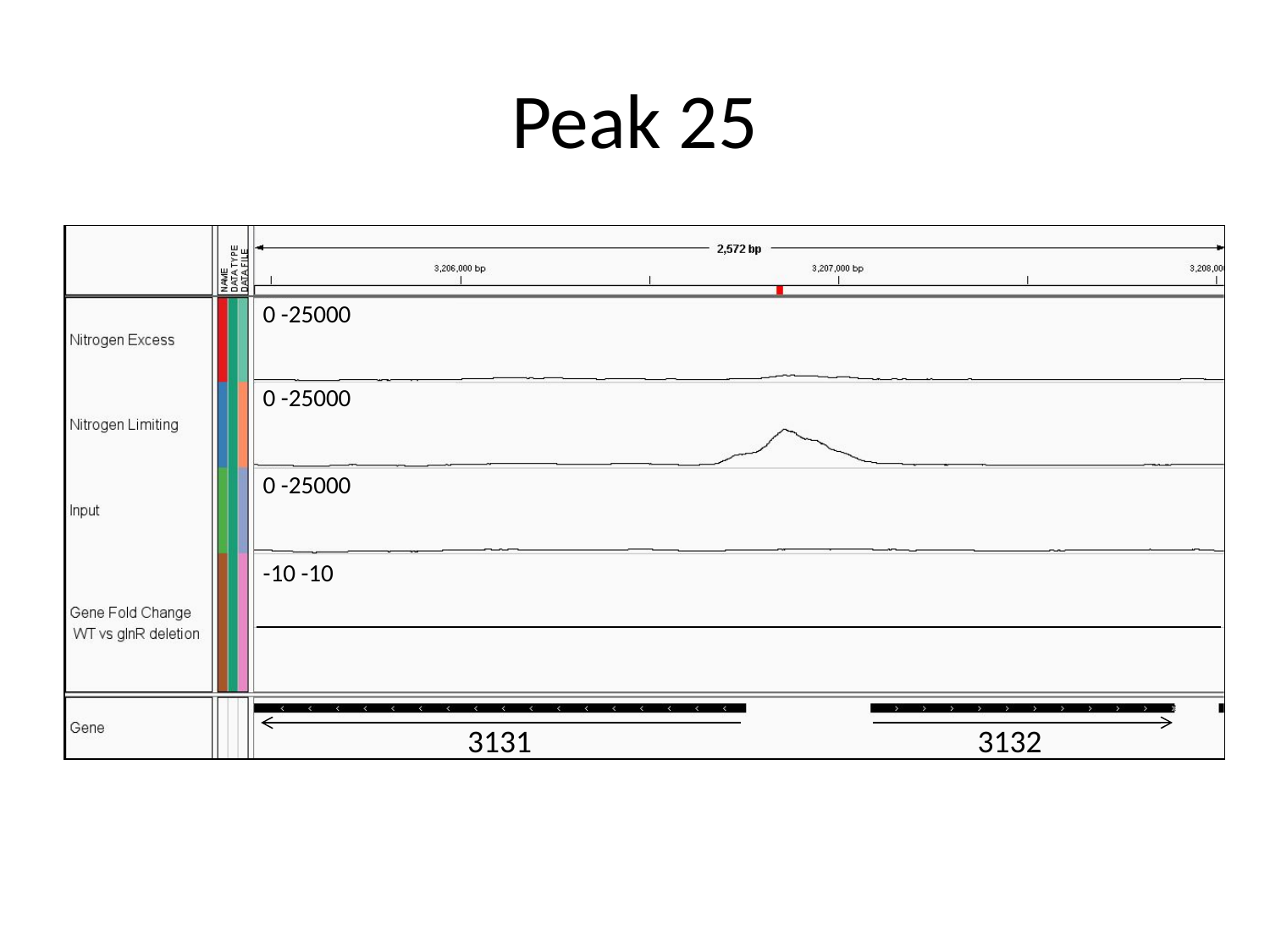

# Peak 25
0 -25000
0 -25000
0 -25000
-10 -10
3132
3131

## Slide 27
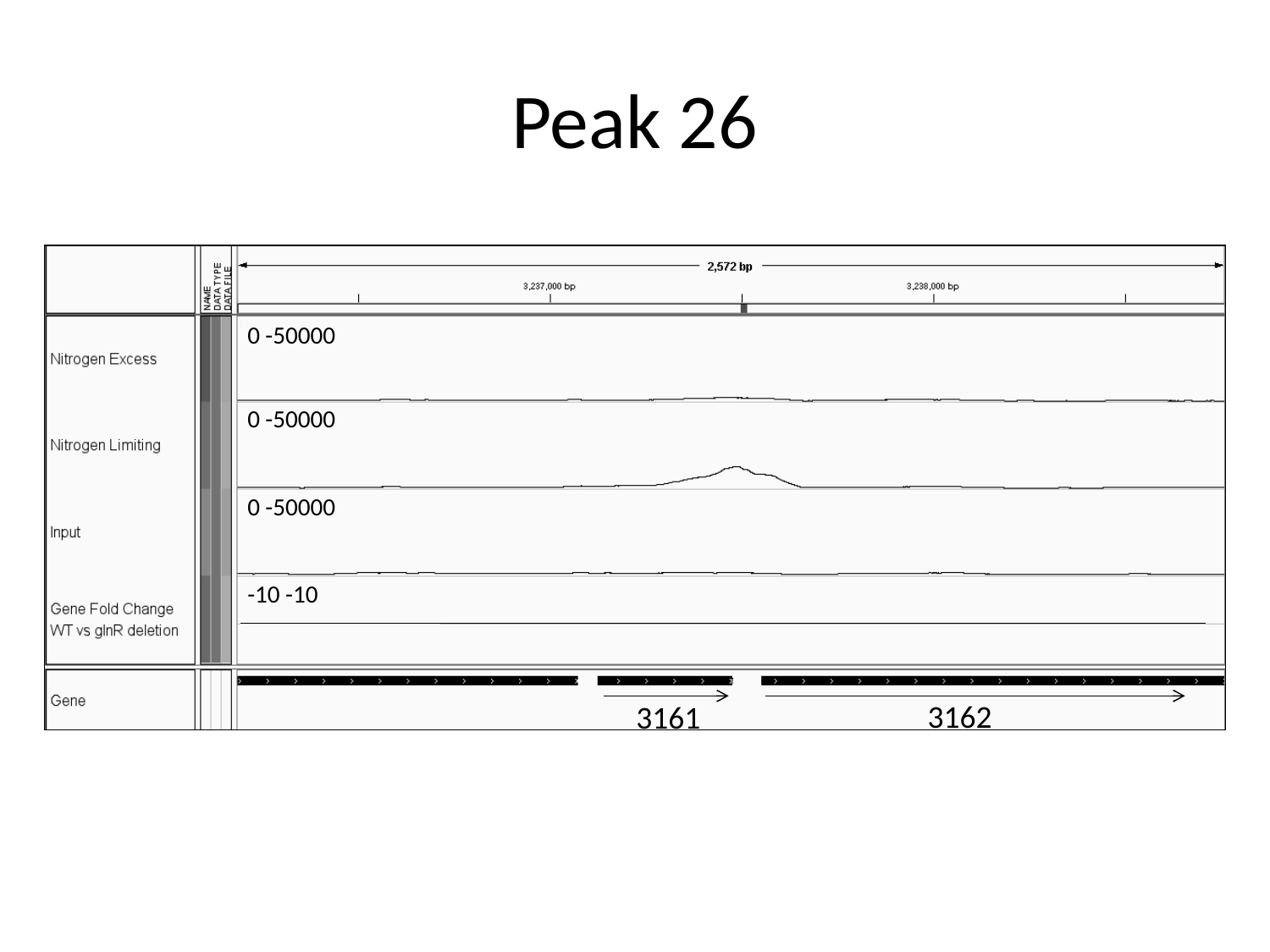

# Peak 26
0 -50000
0 -50000
0 -50000
-10 -10
3162
3161

## Slide 28
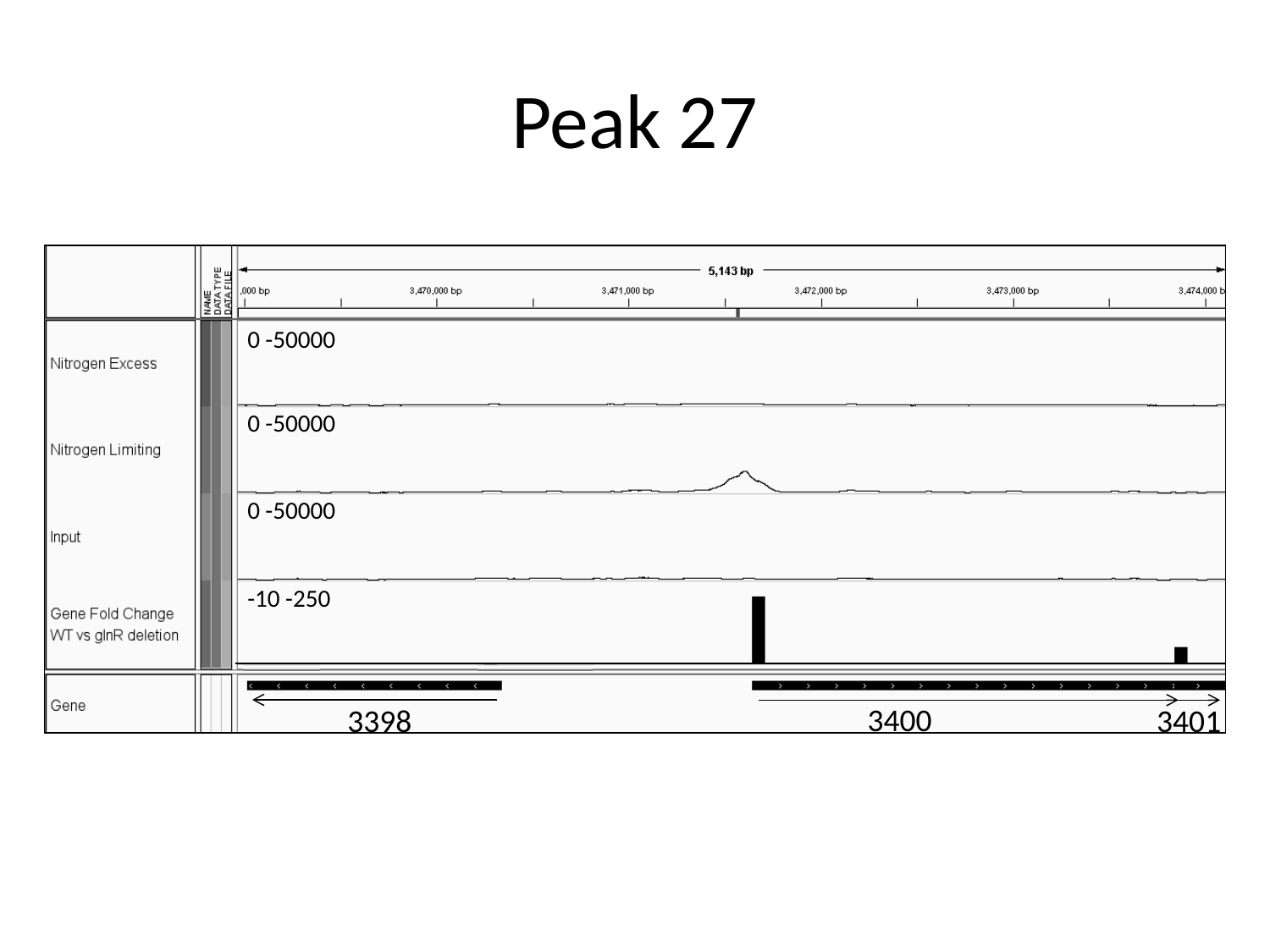

# Peak 27
0 -50000
0 -50000
0 -50000
-10 -250
3400
3401
3398

## Slide 29
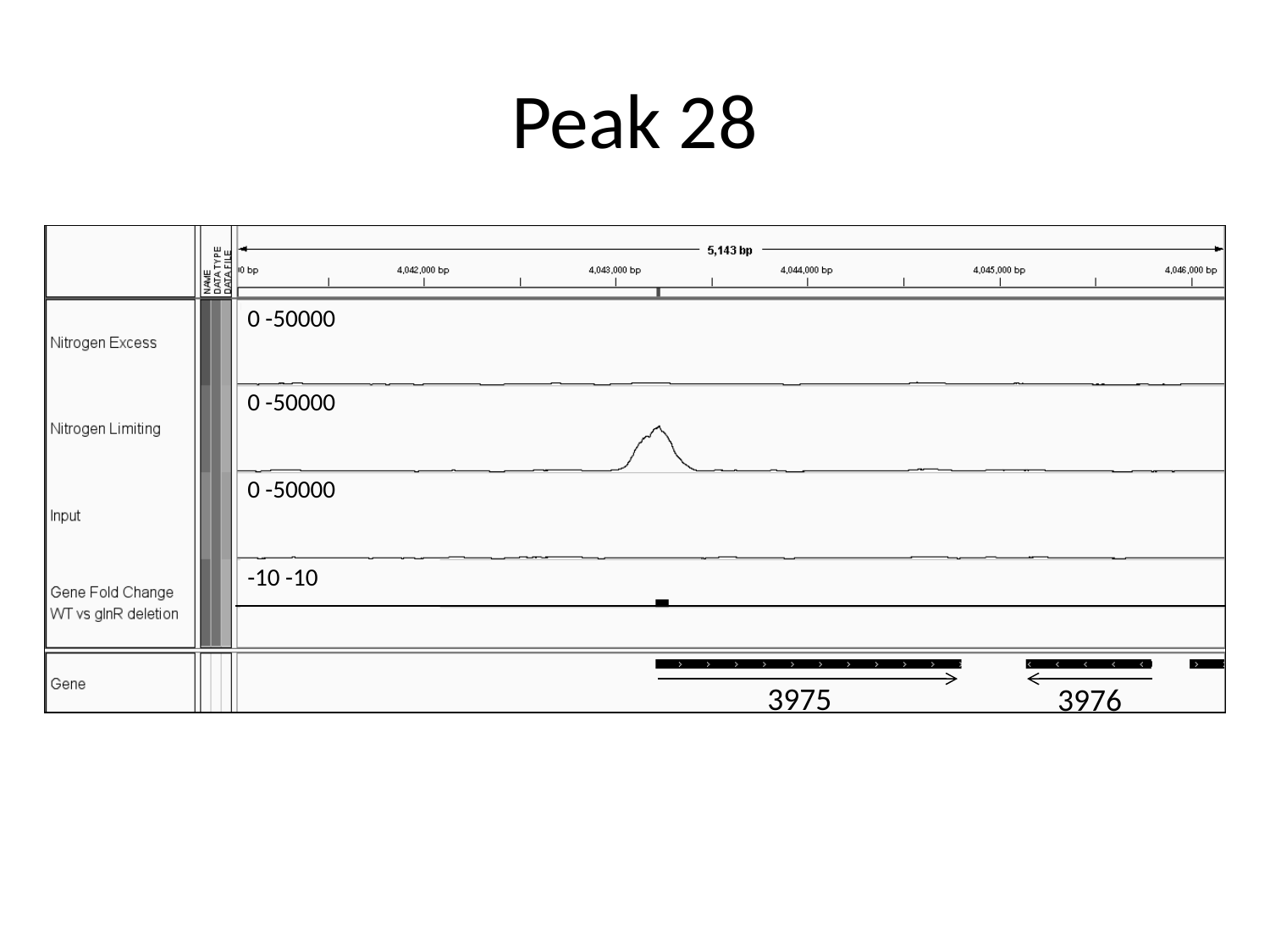

# Peak 28
0 -50000
0 -50000
0 -50000
-10 -10
3975
3976

## Slide 30
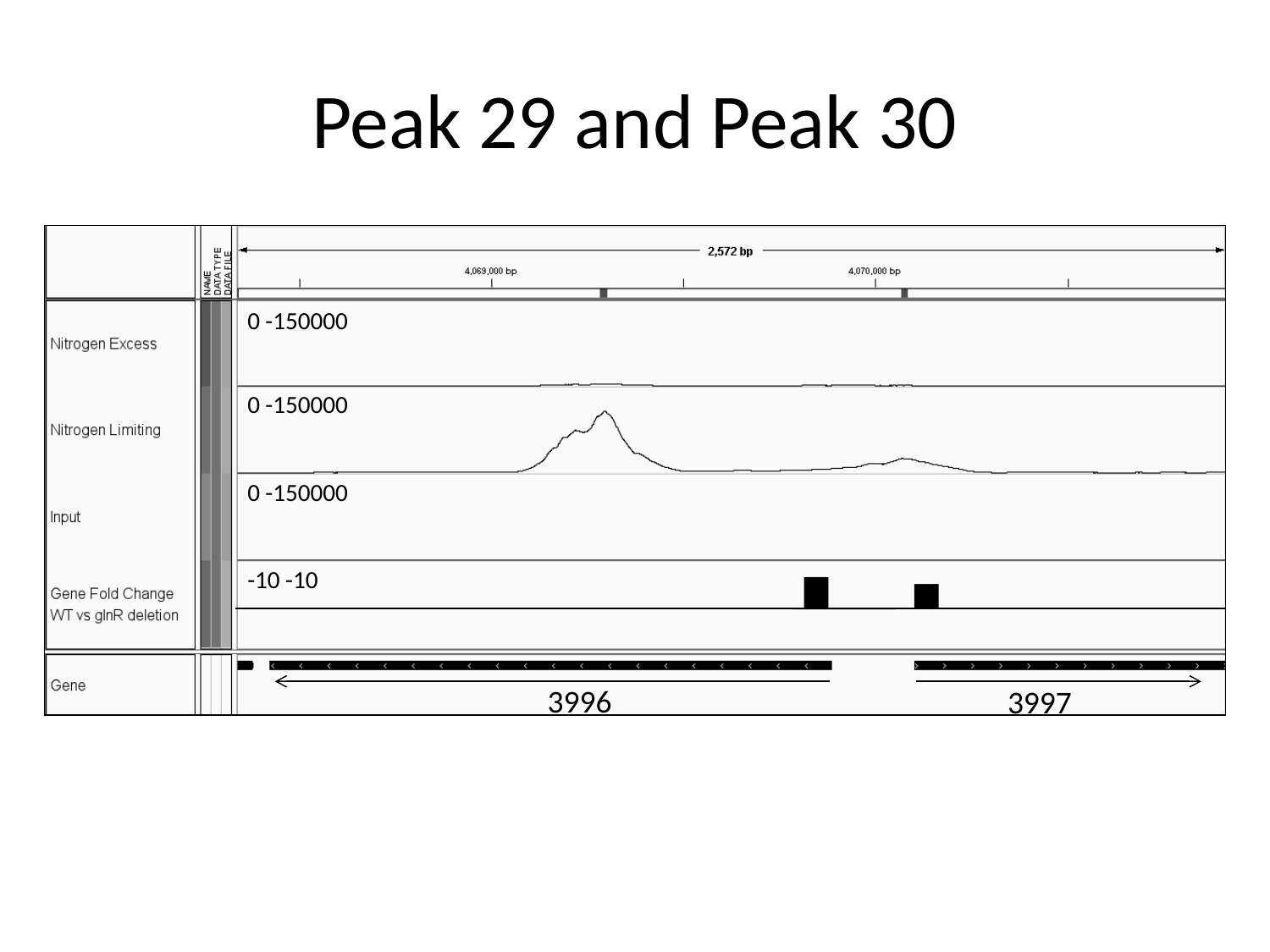

# Peak 29 and Peak 30
0 -150000
0 -150000
0 -150000
-10 -10
3996
3997

## Slide 31
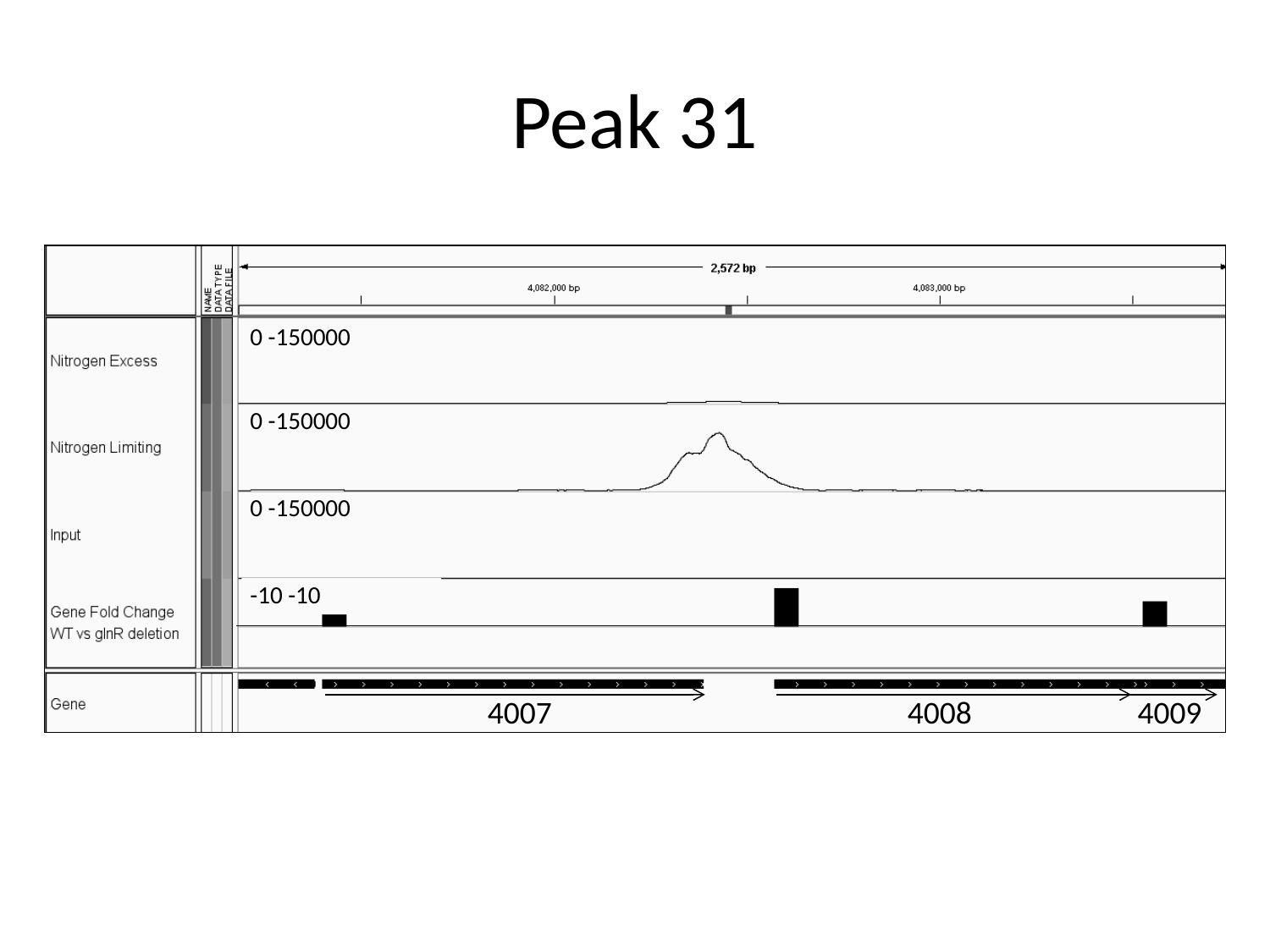

# Peak 31
0 -150000
0 -150000
0 -150000
-10 -10
4008
4009
4007

## Slide 32
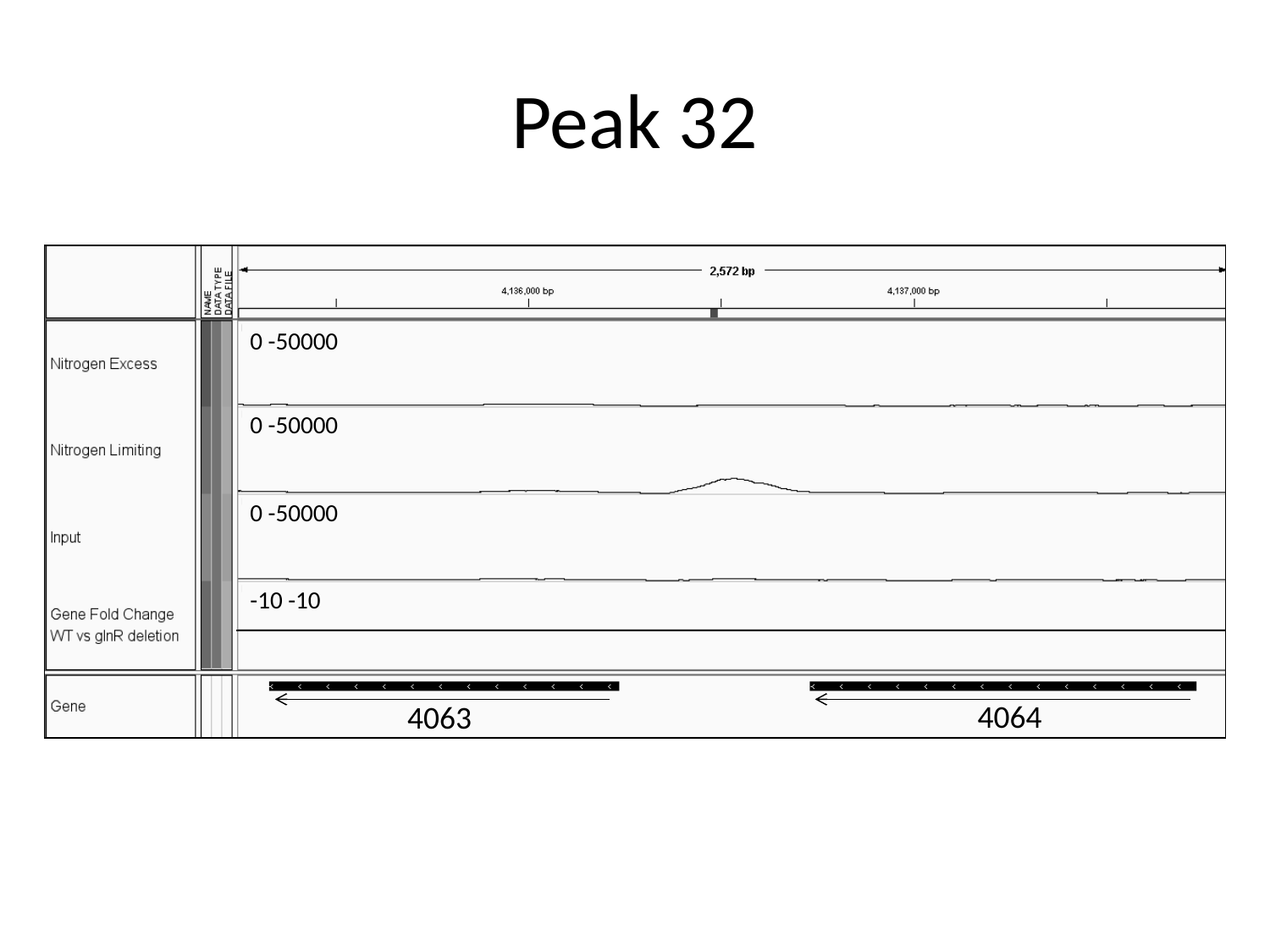

# Peak 32
0 -50000
0 -50000
0 -50000
-10 -10
4064
4063

## Slide 33
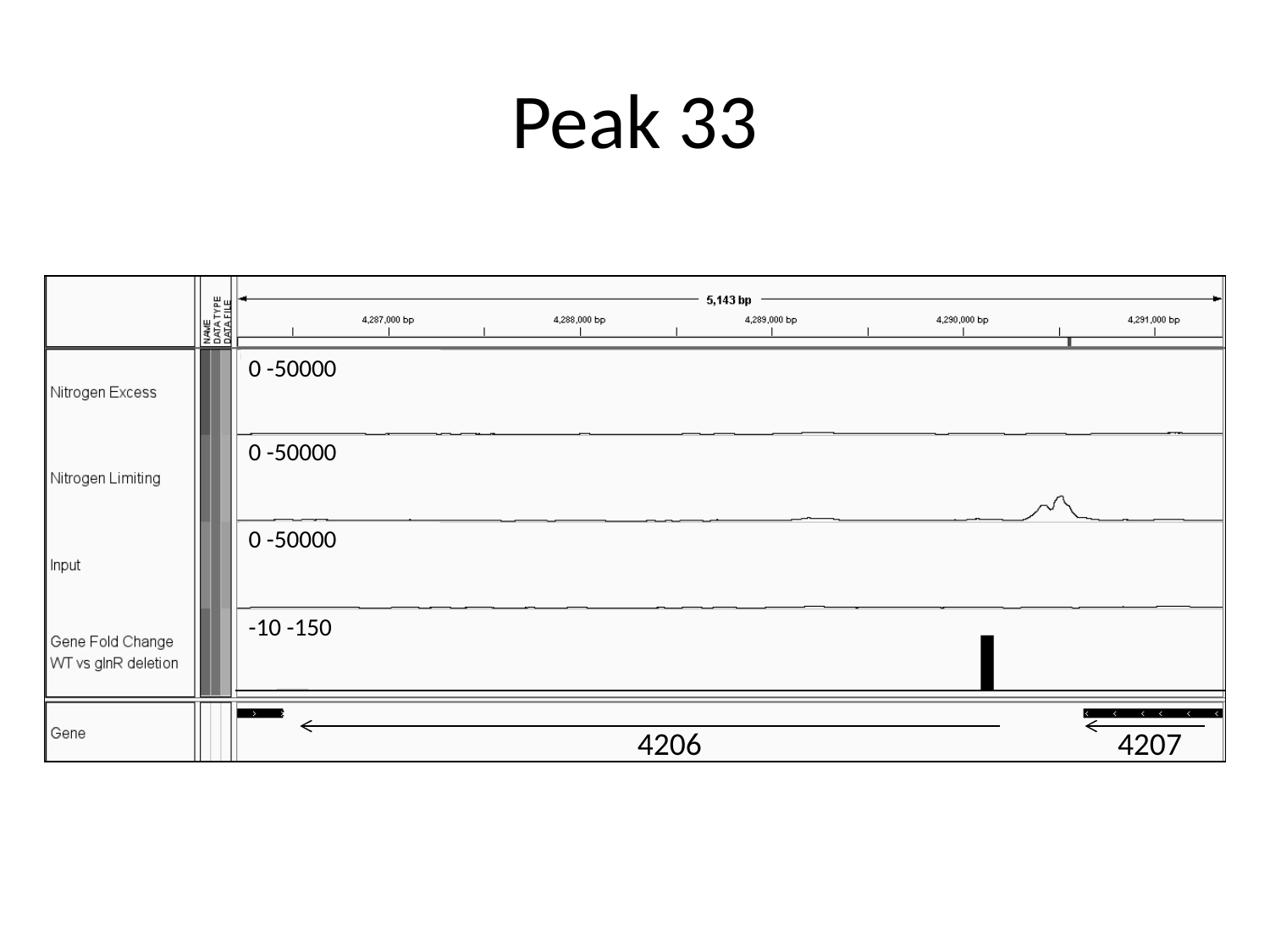

# Peak 33
0 -50000
0 -50000
0 -50000
-10 -150
4207
4206

## Slide 34
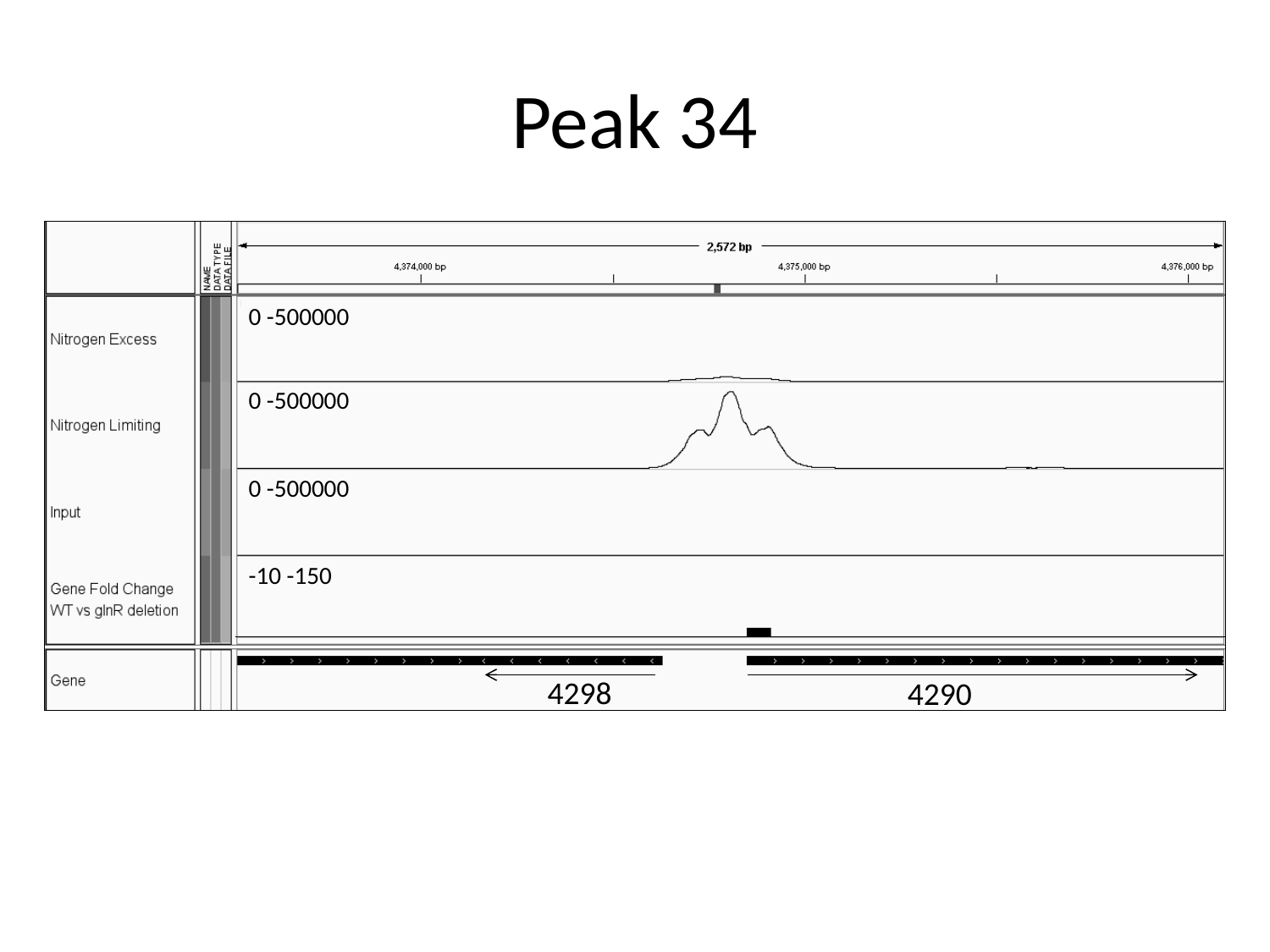

# Peak 34
0 -500000
0 -500000
0 -500000
-10 -150
4298
4290

## Slide 35
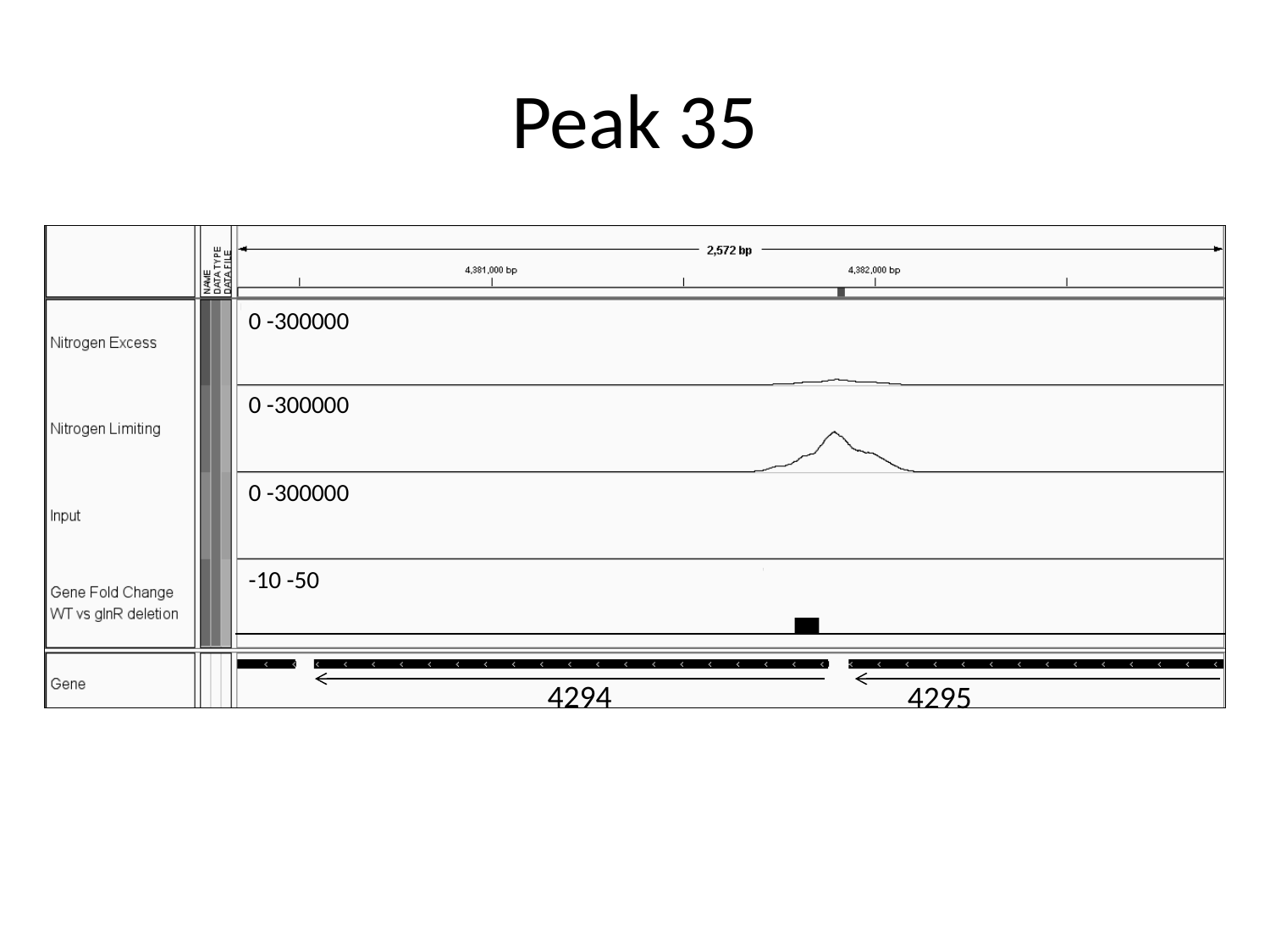

# Peak 35
0 -300000
0 -300000
0 -300000
-10 -50
4294
4295

## Slide 36
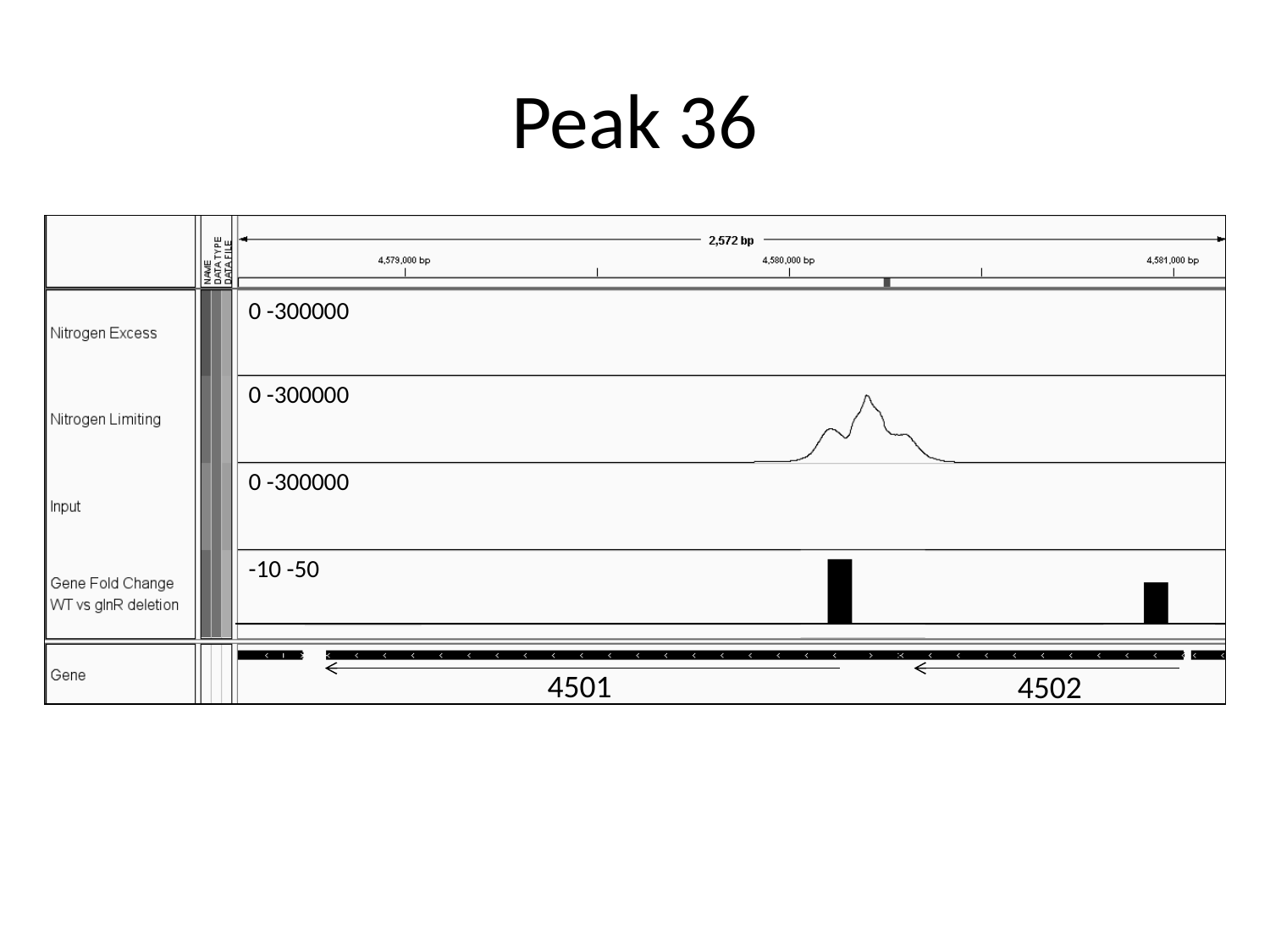

# Peak 36
0 -300000
0 -300000
0 -300000
-10 -50
4501
4502

## Slide 37
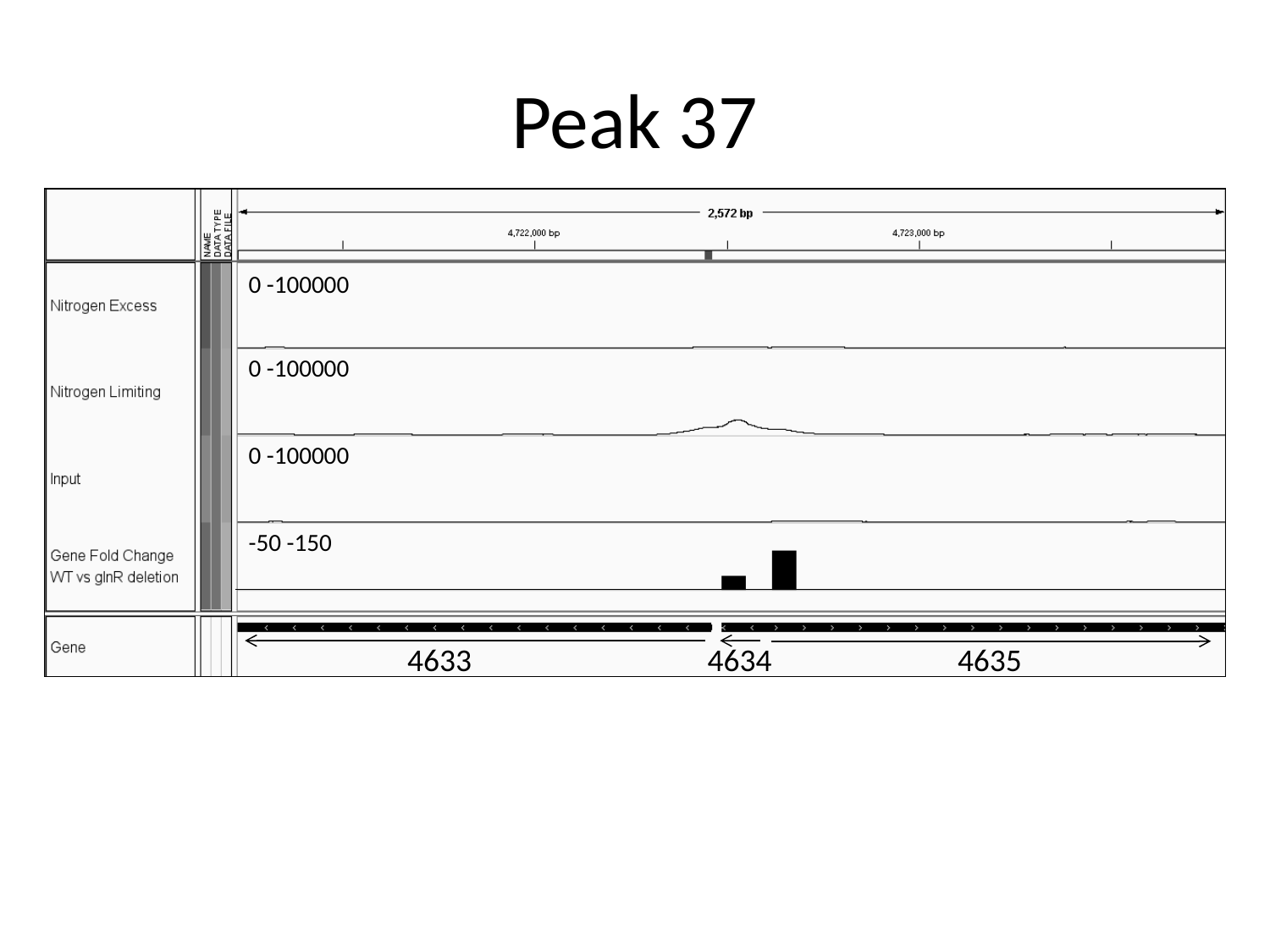

# Peak 37
0 -100000
0 -100000
0 -100000
-50 -150
4634
4635
4633

## Slide 38
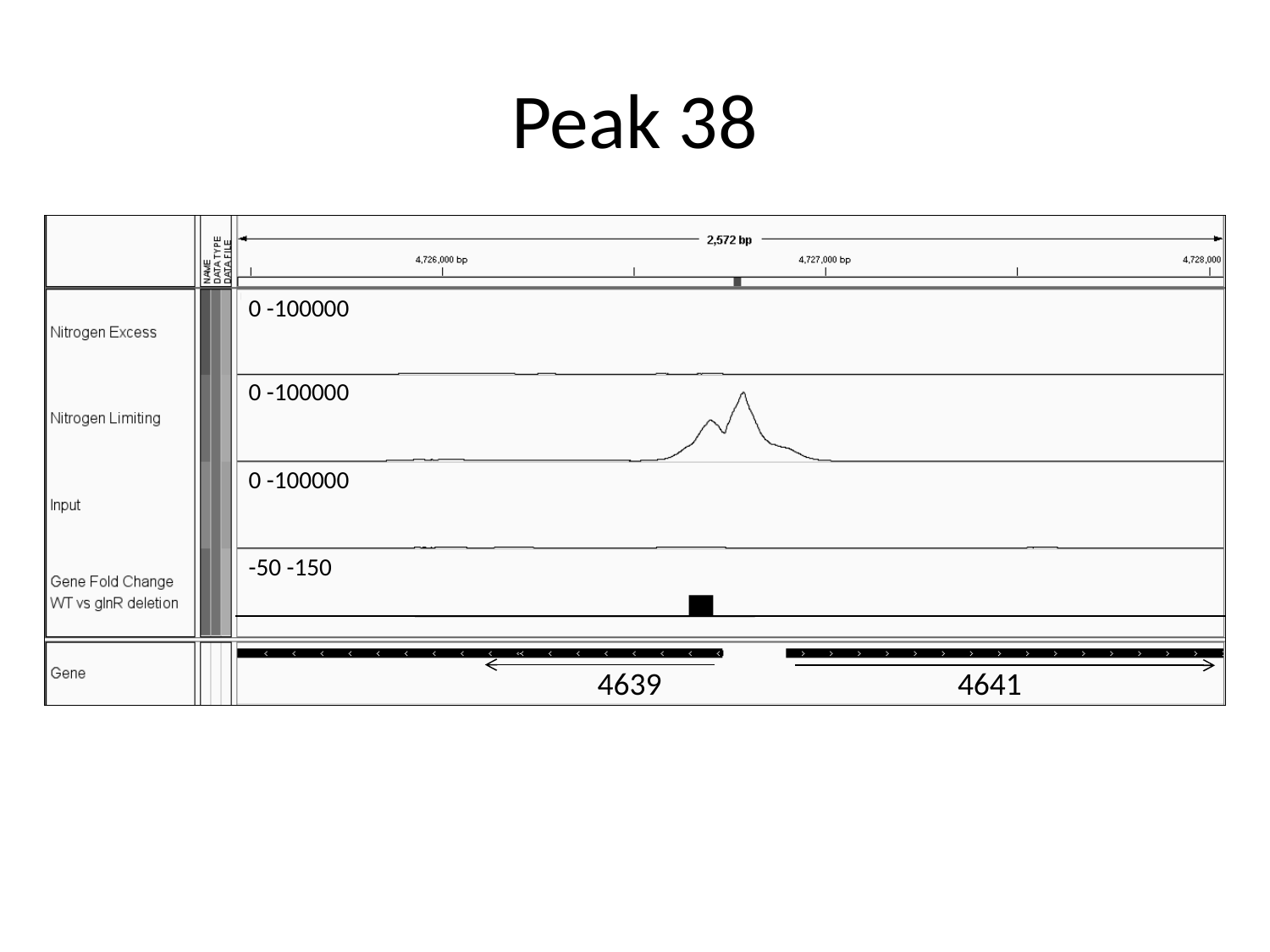

# Peak 38
0 -100000
0 -100000
0 -100000
-50 -150
4641
4639

## Slide 39
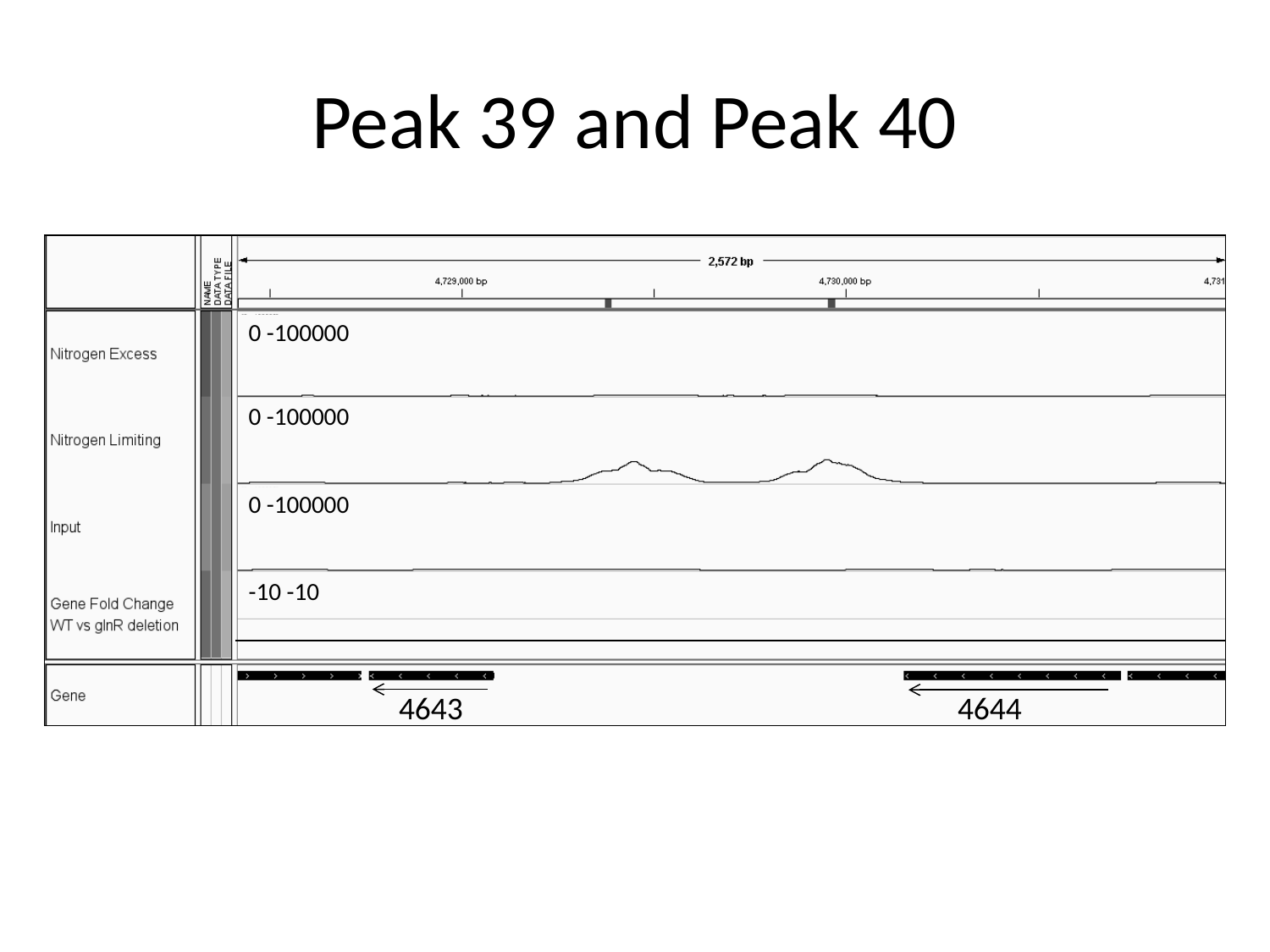

# Peak 39 and Peak 40
0 -100000
0 -100000
0 -100000
-10 -10
4644
4643

## Slide 40
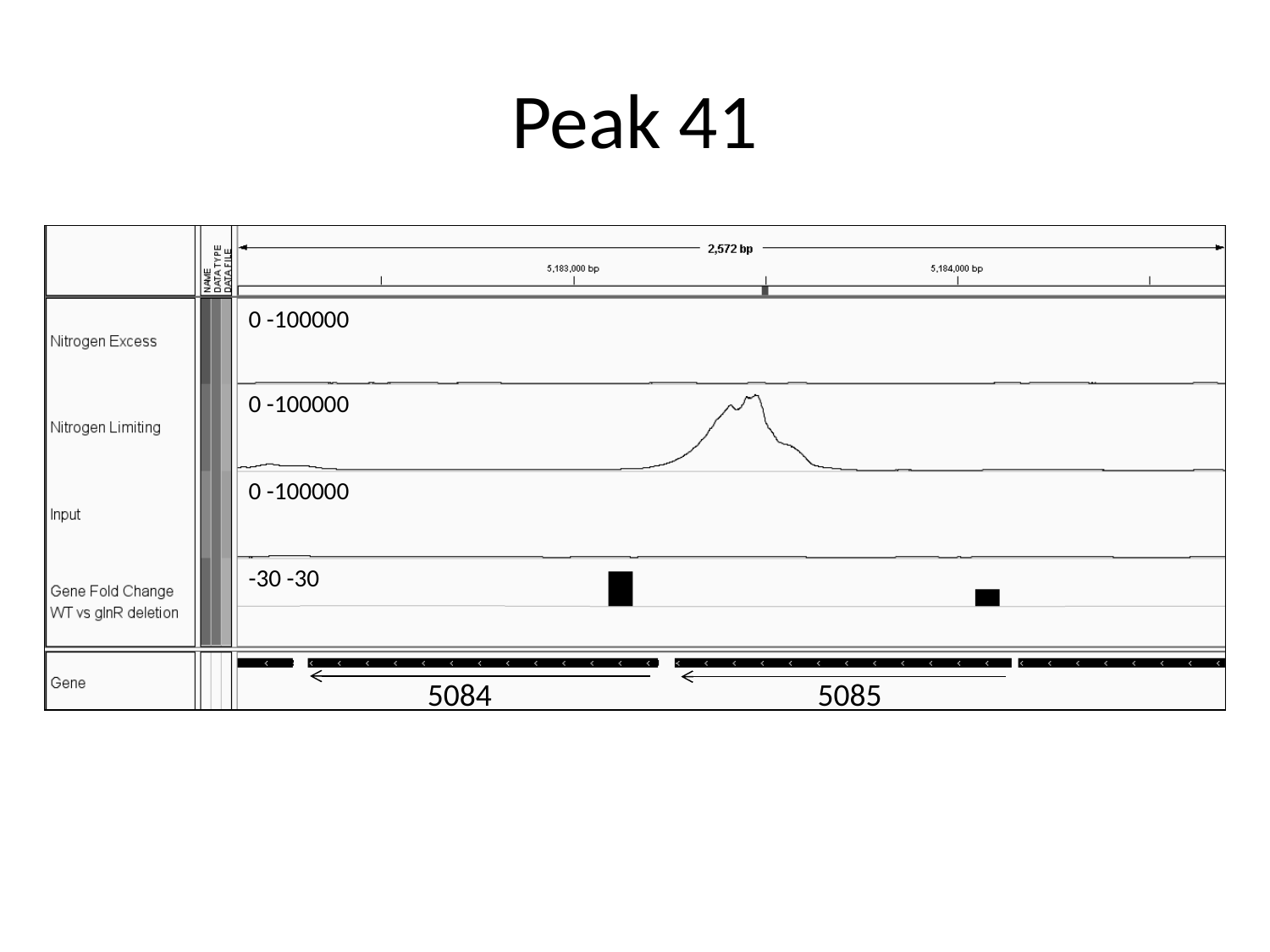

# Peak 41
0 -100000
0 -100000
0 -100000
-30 -30
5085
5084

## Slide 41
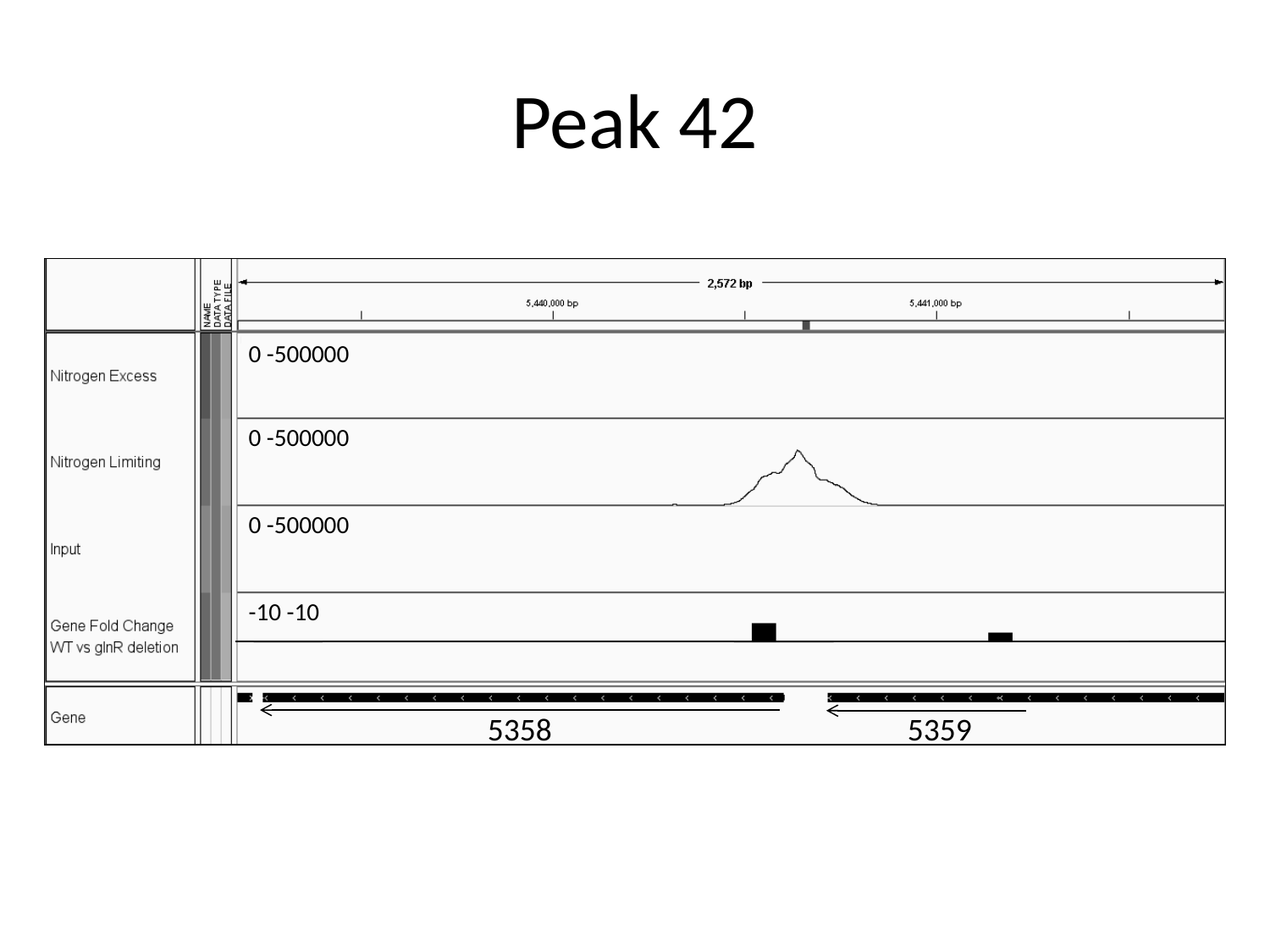

# Peak 42
0 -500000
0 -500000
0 -500000
-10 -10
5359
5358

## Slide 42
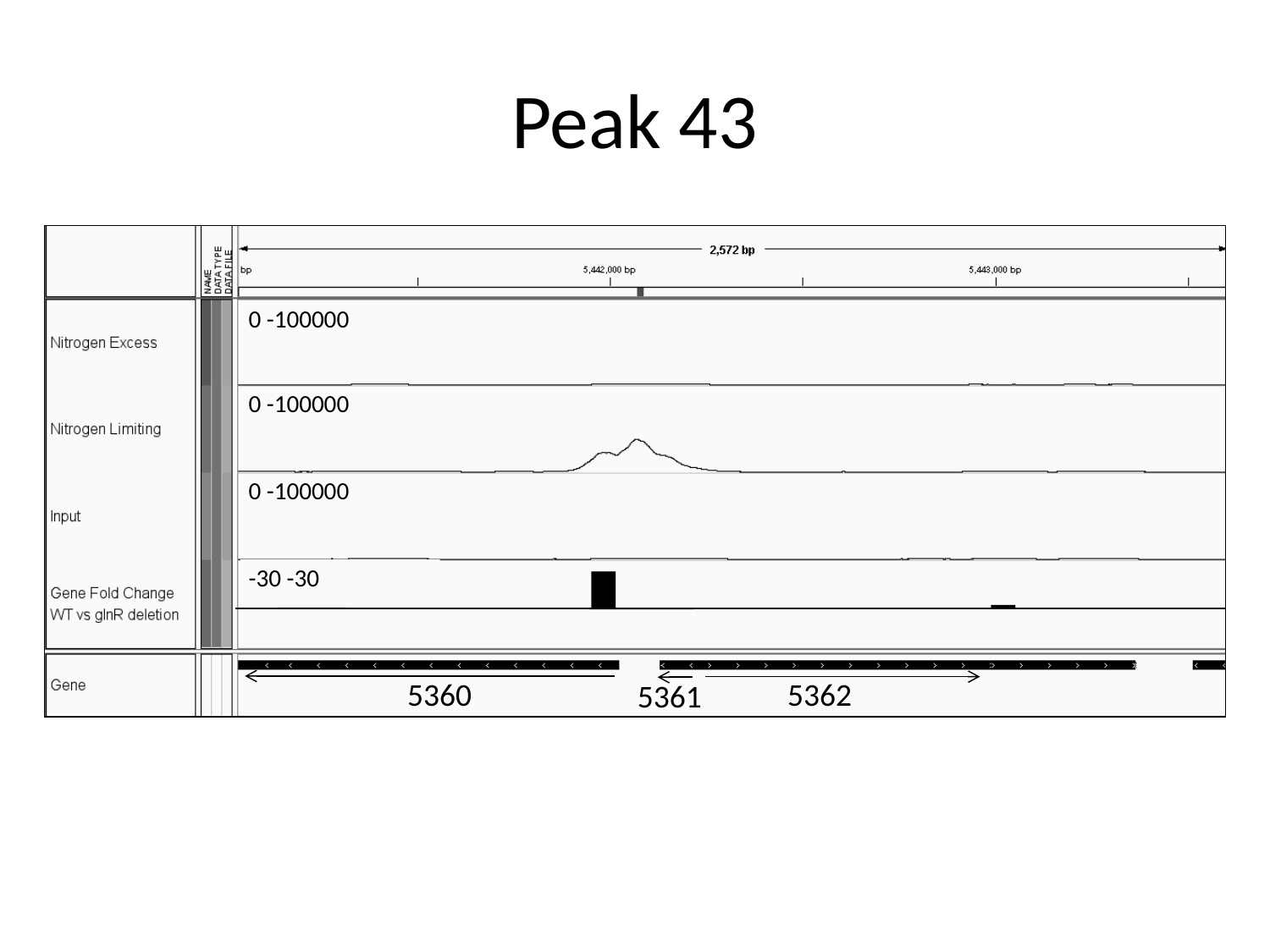

# Peak 43
0 -100000
0 -100000
0 -100000
-30 -30
5362
5360
5361

## Slide 43
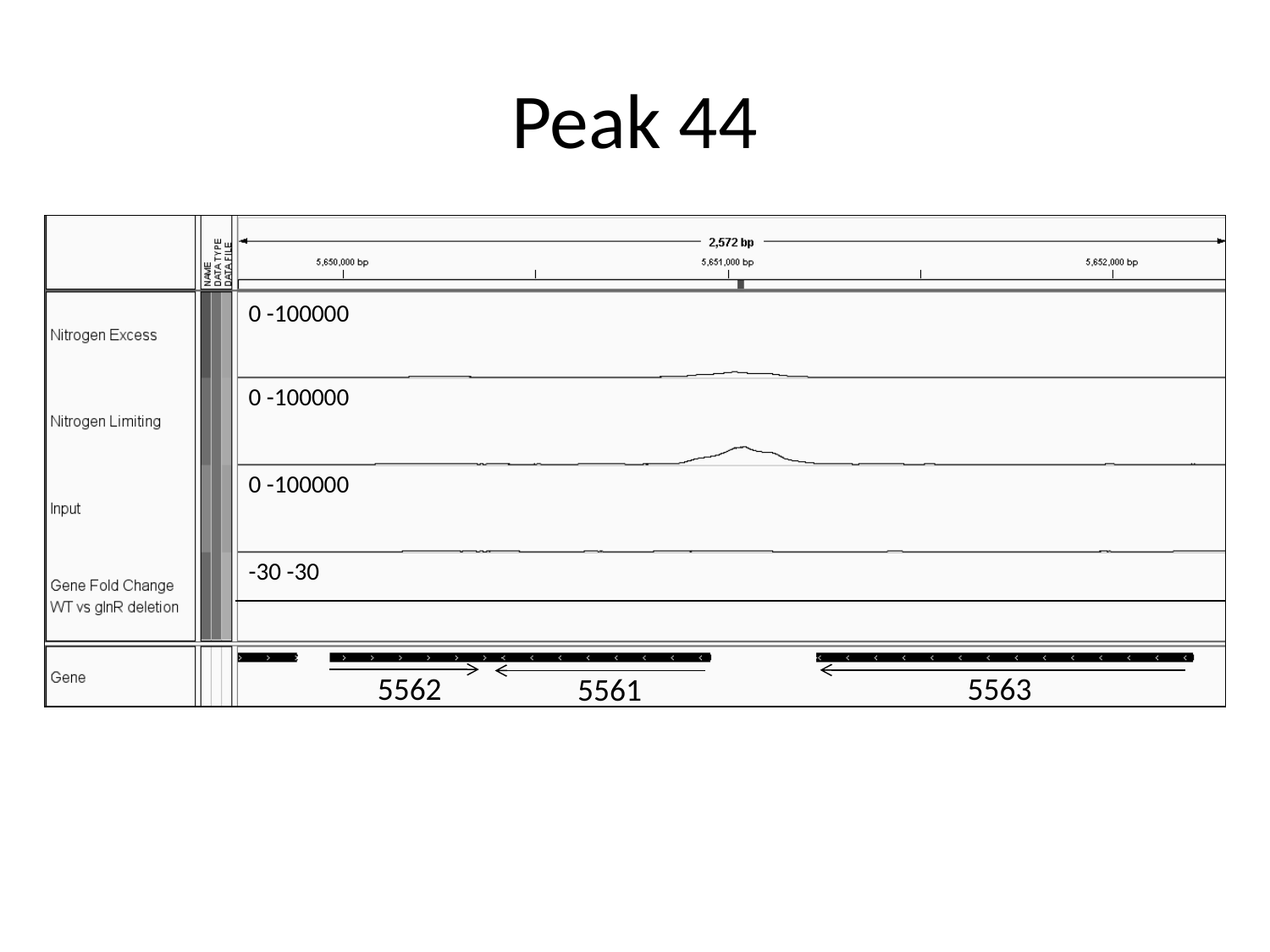

# Peak 44
0 -100000
0 -100000
0 -100000
-30 -30
5563
5562
5561

## Slide 44
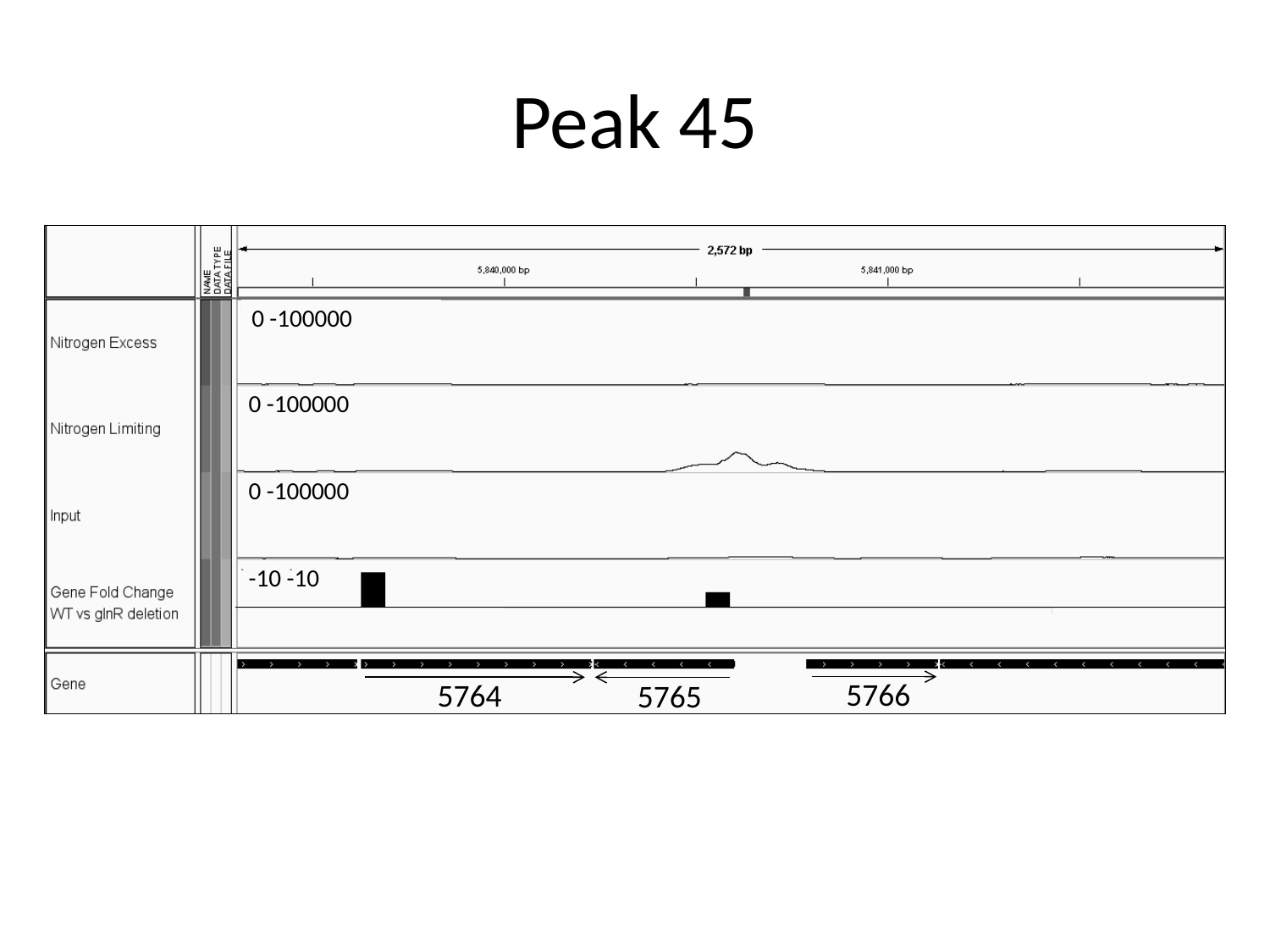

# Peak 45
0 -100000
0 -100000
0 -100000
-10 -10
5766
5764
5765

## Slide 45
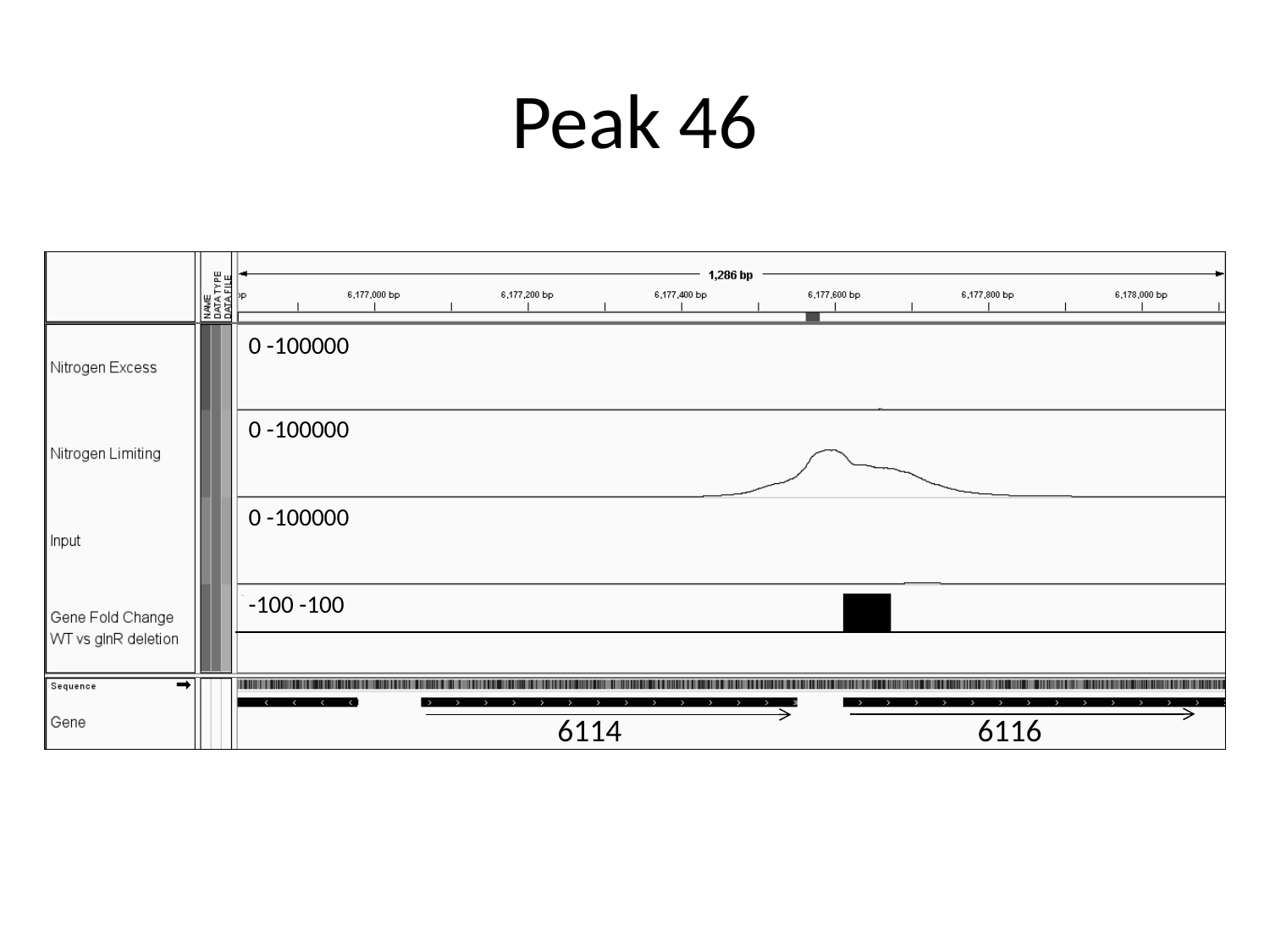

# Peak 46
0 -100000
0 -100000
0 -100000
-100 -100
6116
6114

## Slide 46
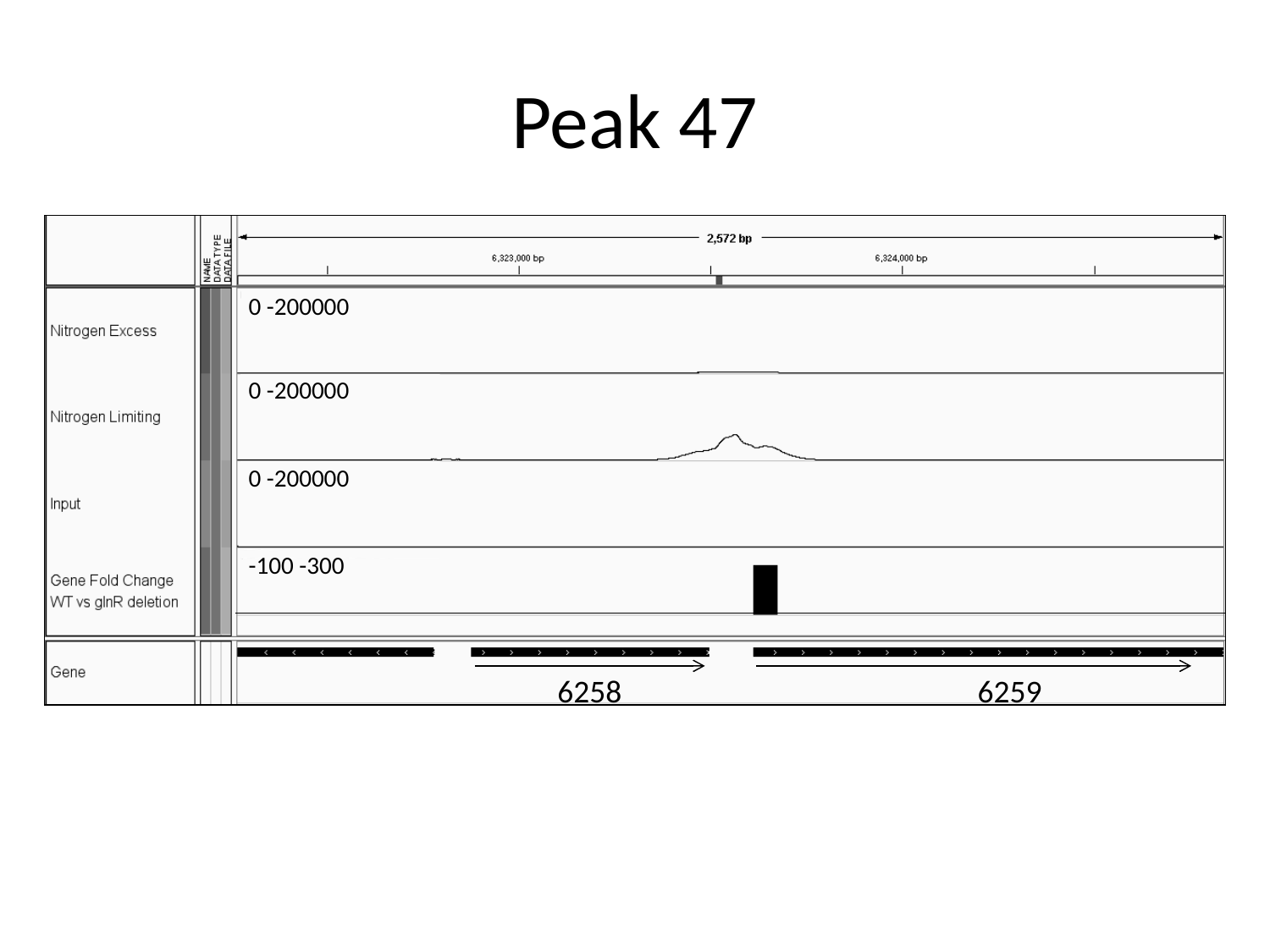

# Peak 47
0 -200000
0 -200000
0 -200000
-100 -300
6259
6258

## Slide 47
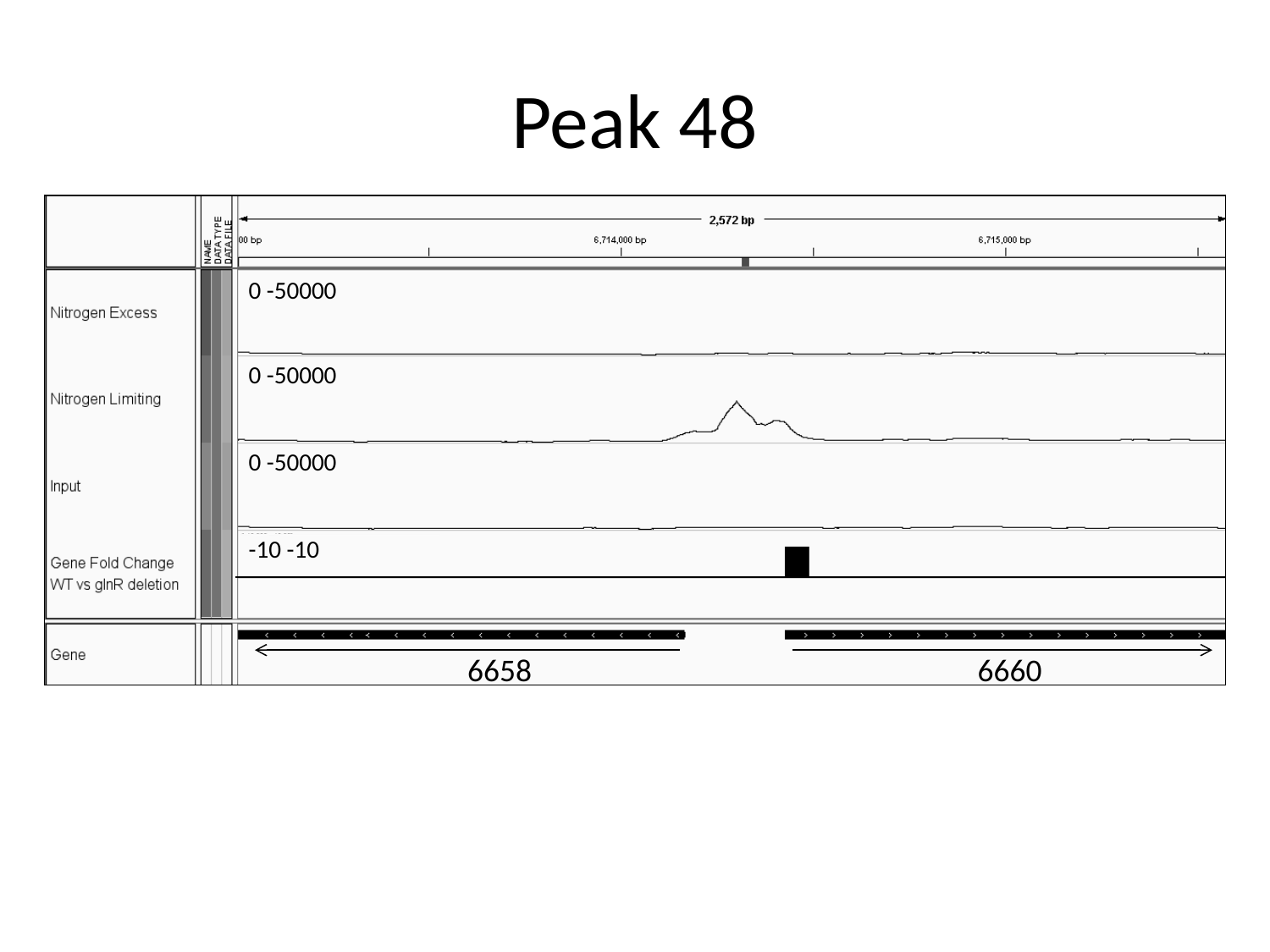

# Peak 48
0 -50000
0 -50000
0 -50000
-10 -10
6660
6658

## Slide 48
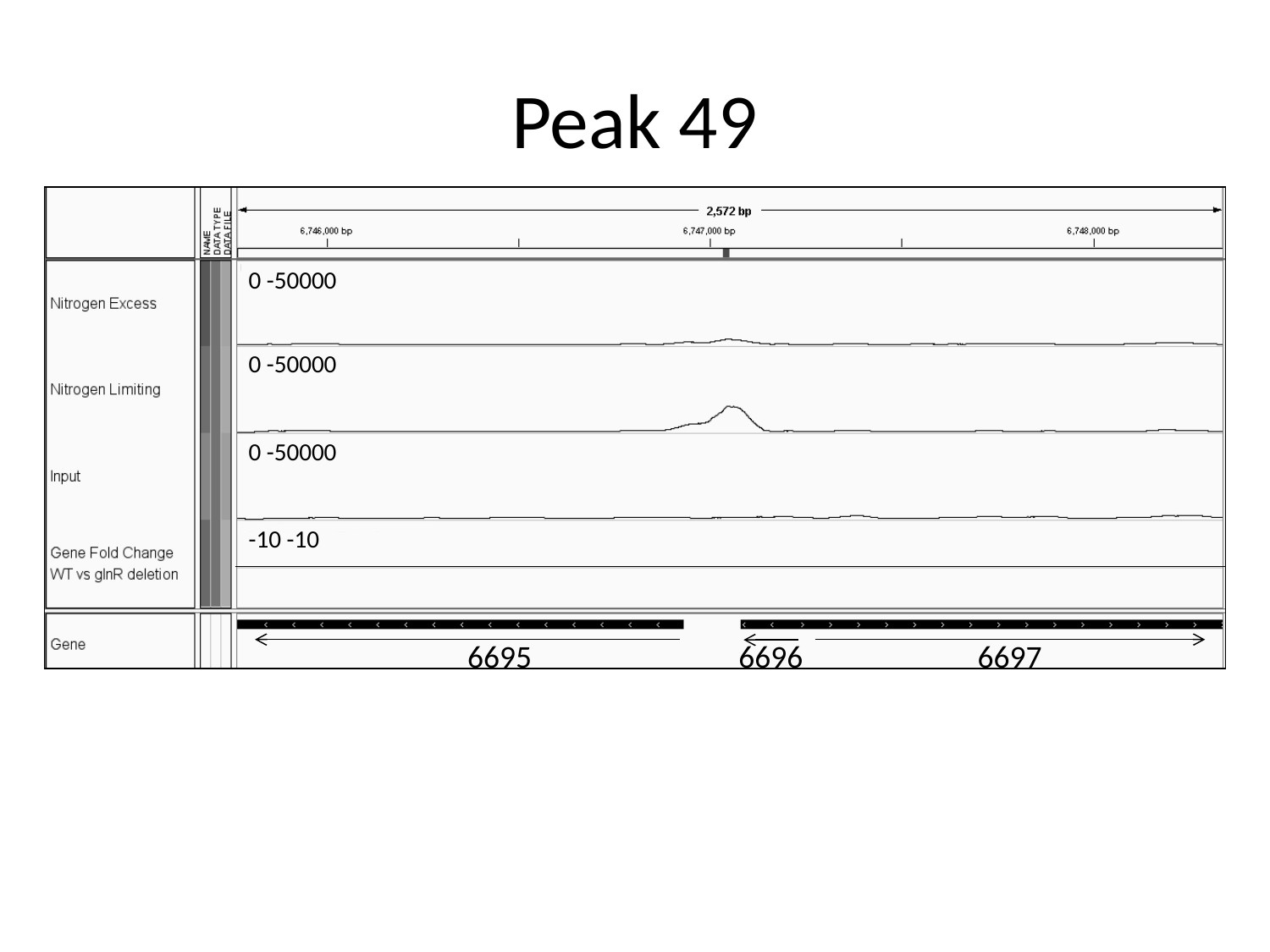

# Peak 49
0 -50000
0 -50000
0 -50000
-10 -10
6697
6695
6696

## Slide 49
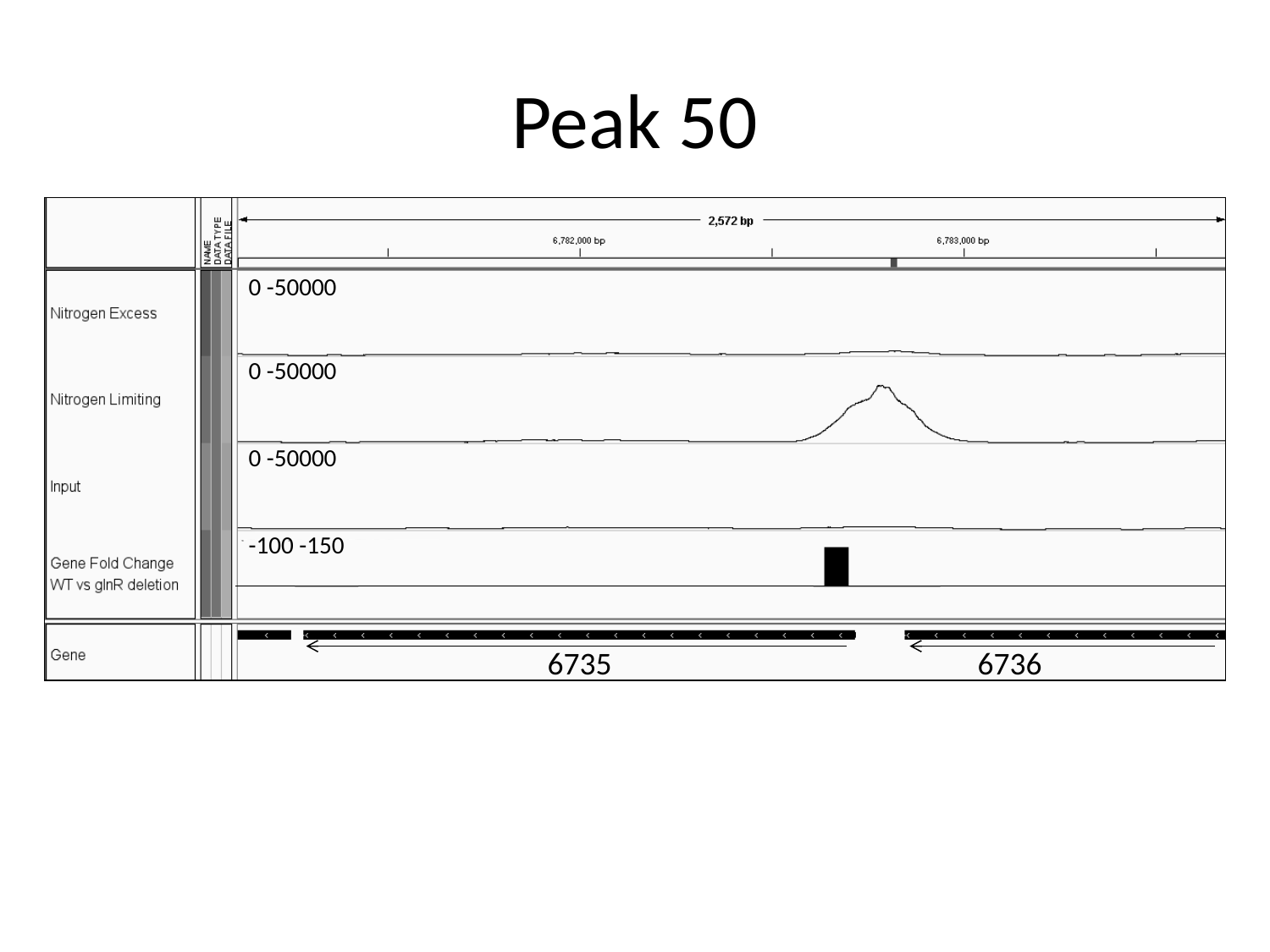

# Peak 50
0 -50000
0 -50000
0 -50000
-100 -150
6736
6735

## Slide 50
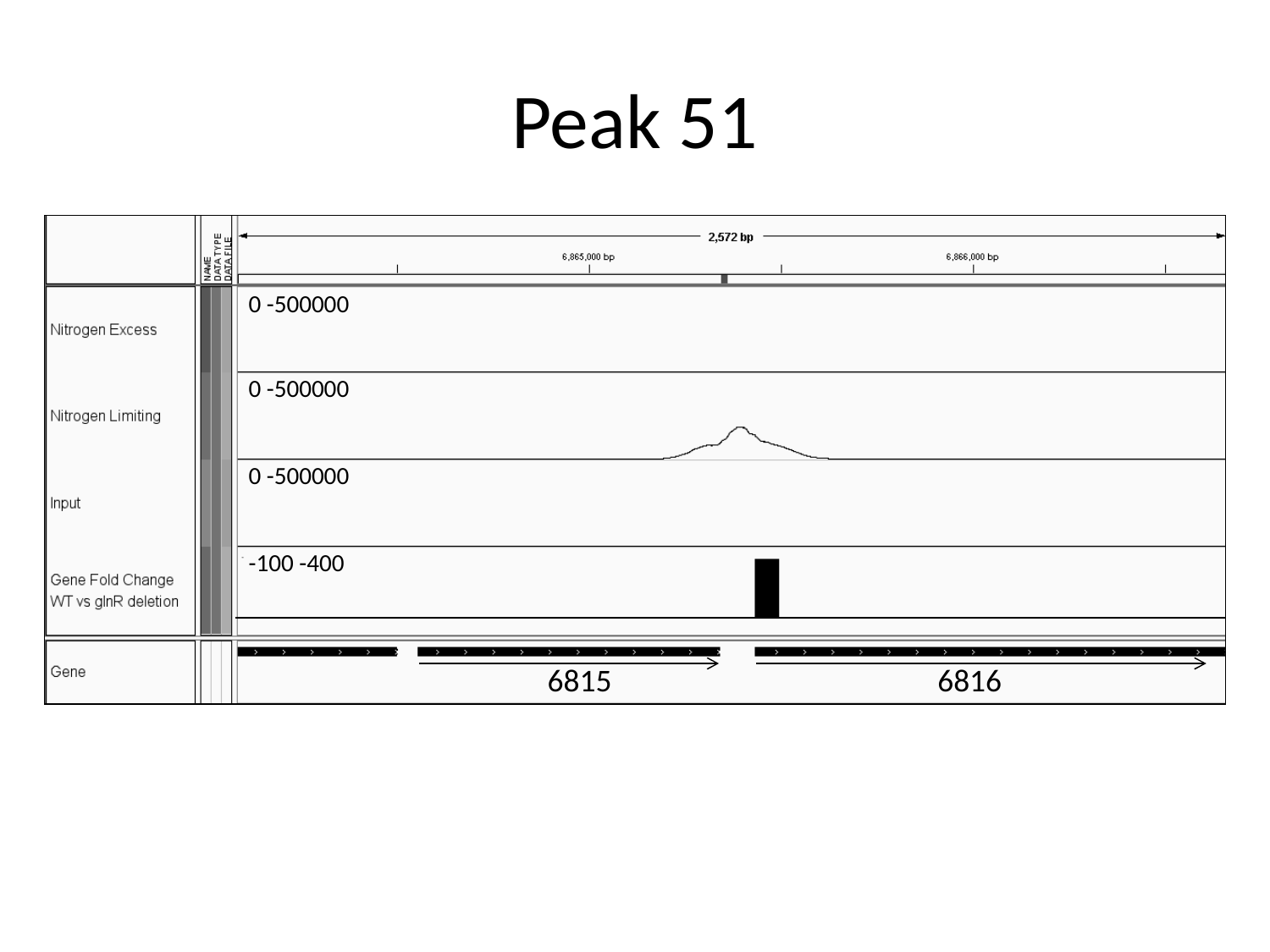

# Peak 51
0 -500000
0 -500000
0 -500000
-100 -400
6816
6815

## Slide 51
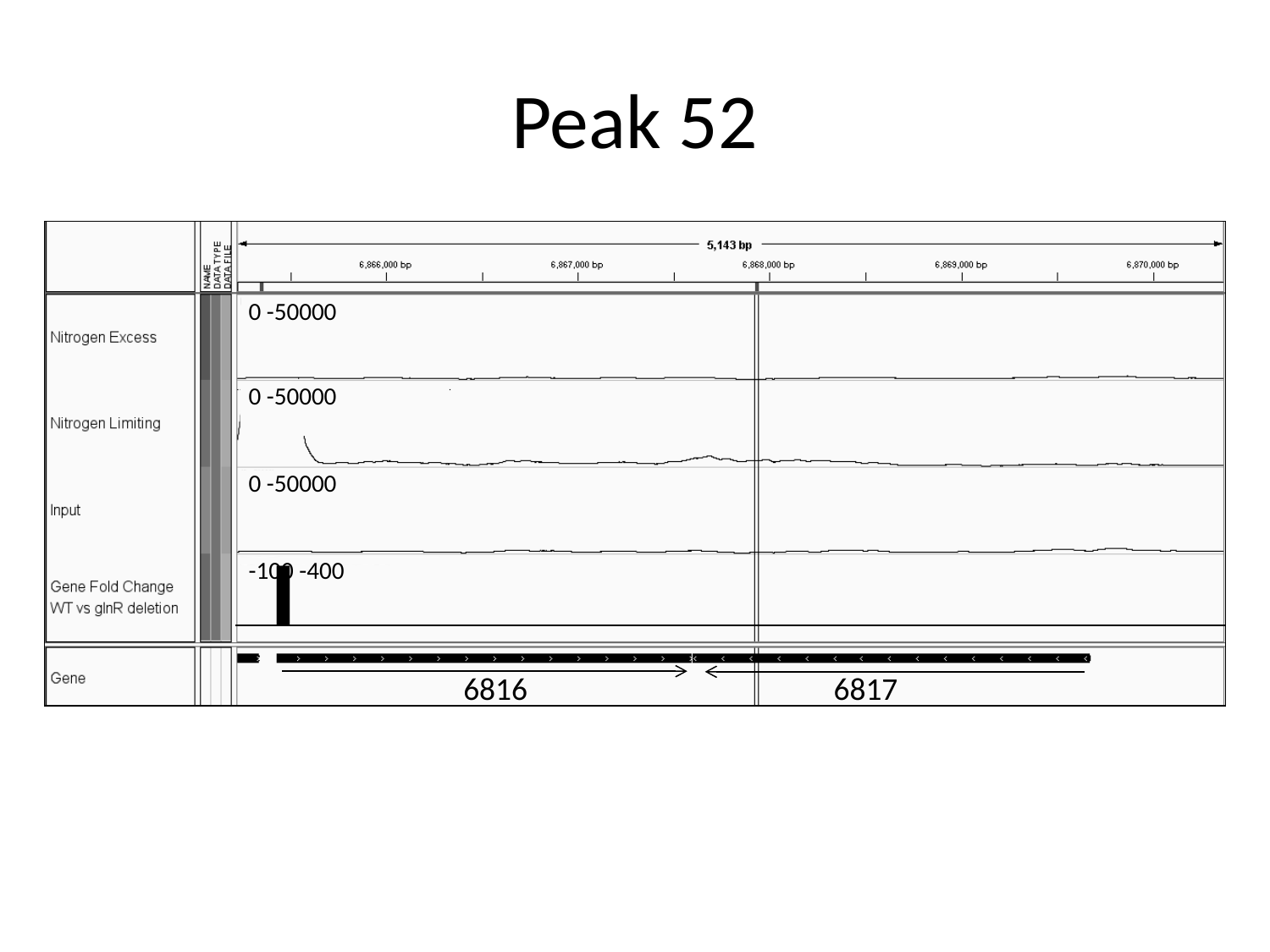

# Peak 52
0 -50000
0 -50000
0 -50000
-100 -400
6816
6817

## Slide 52
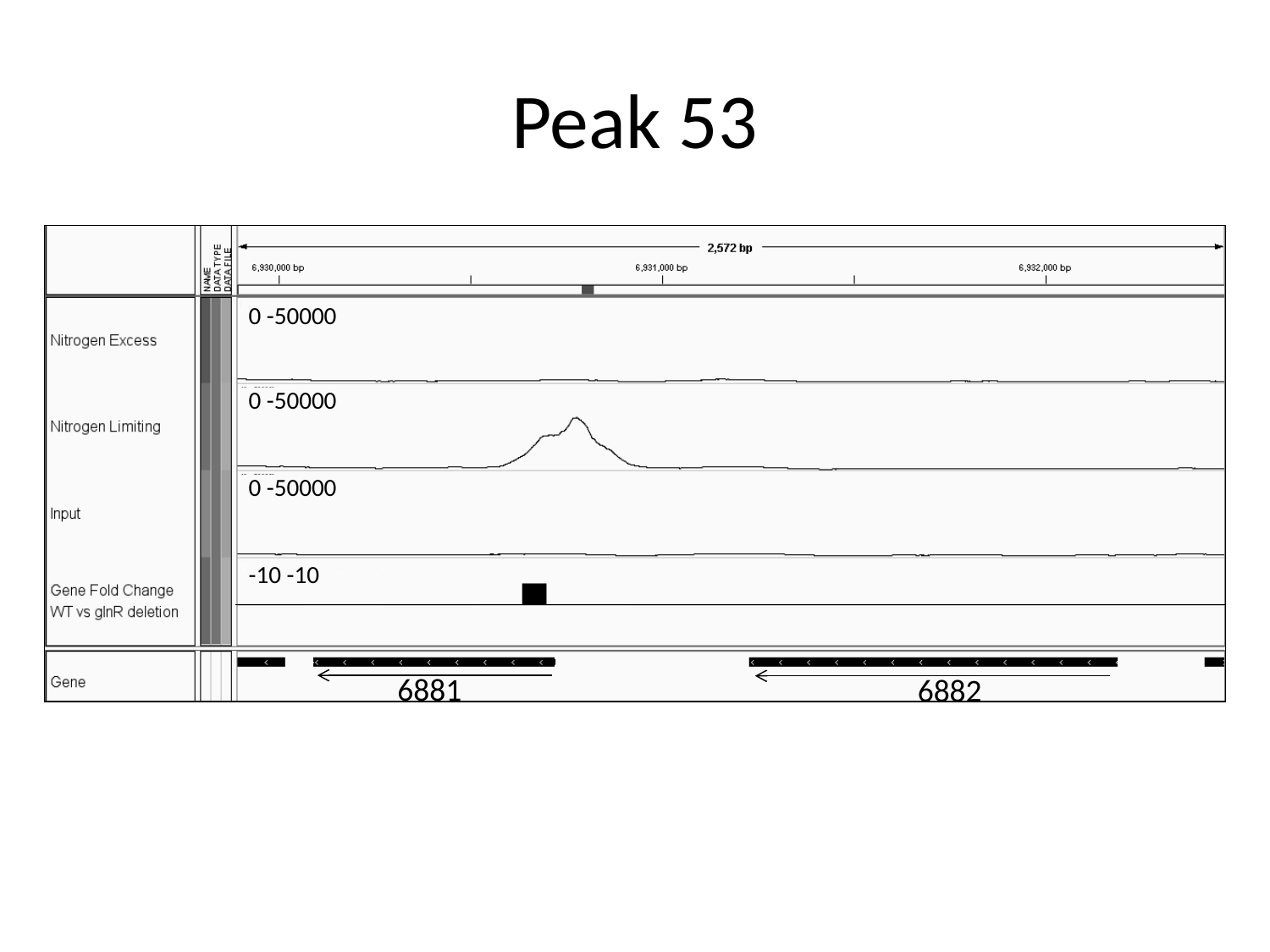

# Peak 53
0 -50000
0 -50000
0 -50000
-10 -10
6881
6882
