# Supplementary figures and images for: Genome wide analysis of the complete GlnR nitrogen-response regulon in Mycobacterium smegmatis
Source: BMC Genomics. 2013 May 4;14:301. doi: 10.1186/1471-2164-14-301 (PMC3662644; doi:10.1186/1471-2164-14-301)

## Slide 1
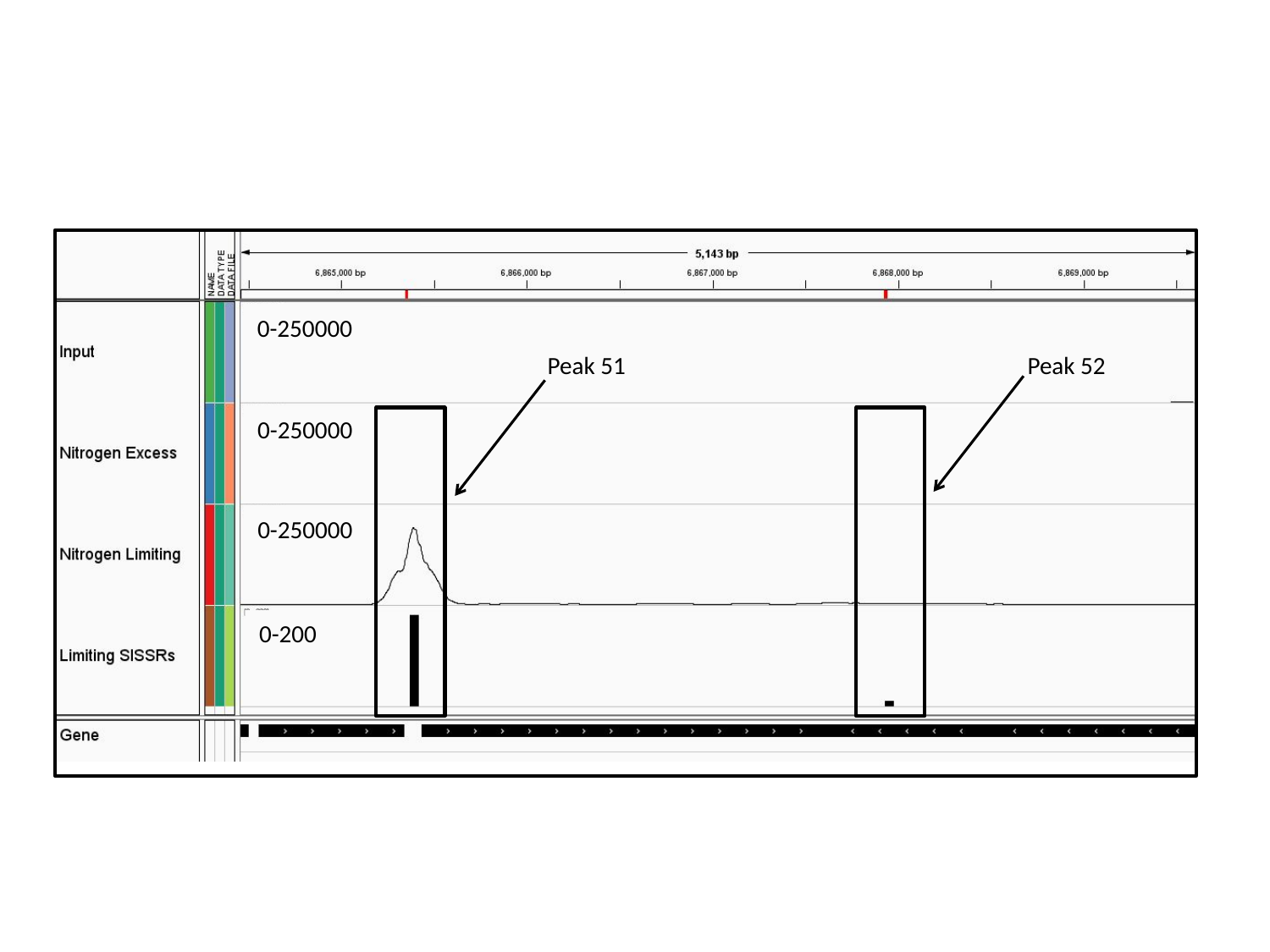

0-250000
Peak 51
Peak 52
0-250000
0-250000
0-200

Supplement: Additional file 4: Figure S3 — Screenshot from IGV showing peaks 51 and 52 and highlighting the mis-calling of peak 52 by SISSRs. Binding data was visualised using IGV. Upper track indicates ChIP-seq data from the Input sample representing the total DNA, middle track is nitrogen excess conditions and then ChIP-seq data from nitrogen limiting conditions. Aligned to the bottom track is the SISSRs value for the peaks highlighted by the vertical black bars. [file 1471-2164-14-301-S4.pptx]
