# Supplementary material for: Genome wide analysis of the complete GlnR nitrogen-response regulon in Mycobacterium smegmatis
Source: BMC Genomics. 2013 May 4;14:301. doi: 10.1186/1471-2164-14-301 (PMC3662644; doi:10.1186/1471-2164-14-301)
Supplement: Additional file 5: Figure S4 — Rate limiting qPCR confirmed enrichment of the 8 putative GlnR binding sites during nitrogen limitation. (A) Promoter region of MSMEG3224 (negative control), (B) Promoter region of peak 13, (C) Promoter region of peak 14, (D) Promoter region of peak 26, (E) Promoter region of peak 32, (F) Promoter region of peak 39, (G) Promoter region of peak 40, (H) Promoter region of peak 44 and (I) Promoter region of peak 49. Rate-limiting PCR involving 23 cycles of amplification, with 0.3 ng of GlnR-immunoprecipitated DNA from nitrogen excess and limiting conditions. Input excess and input limiting represents the total DNA subject to immunoprecipitation from the excess and limiting samples respectively. [file 1471-2164-14-301-S5.pptx]

## Slide 1
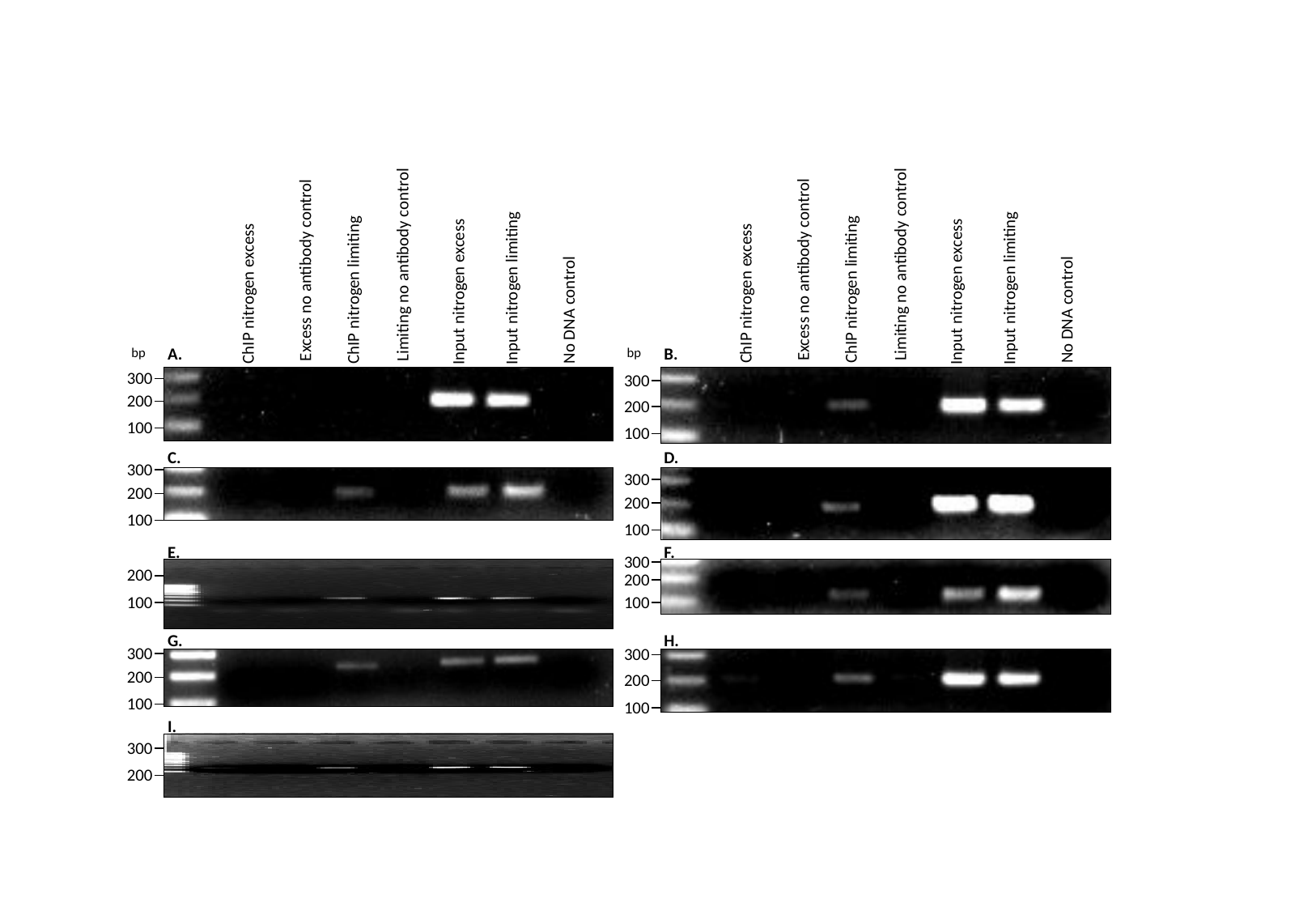

Excess no antibody control
Limiting no antibody control
Excess no antibody control
Limiting no antibody control
Input nitrogen limiting
Input nitrogen limiting
ChIP nitrogen limiting
ChIP nitrogen limiting
Input nitrogen excess
Input nitrogen excess
ChIP nitrogen excess
ChIP nitrogen excess
No DNA control
No DNA control
A.
B.
bp
bp
300
300
200
200
100
100
C.
D.
300
300
200
200
100
100
E.
F.
300
200
200
100
100
G.
H.
300
300
200
200
100
100
I.
300
200
