# Supplementary material for: Genome wide analysis of the complete GlnR nitrogen-response regulon in Mycobacterium smegmatis
Source: BMC Genomics. 2013 May 4;14:301. doi: 10.1186/1471-2164-14-301 (PMC3662644; doi:10.1186/1471-2164-14-301)
Supplement: Additional file 6: Figure S5 — Confirmation of specific GlnR binding to the 200 bp region representing peak 13 by EMSA with the corresponding peak in nitrogen limiting conditions in IGV. EMSAs were performed by incubating increasing amounts of His-GlnR recombinant protein with labelled DNA corresponding to the GlnR binding site peak 13. The addition of non-specific DNA did not affect GlnR binding, confirming this as a specific GlnR binding site. GlnR binding was visualised in IGV. Upper track indicates ChIP-seq data from the Input sample representing the total DNA, middle track is nitrogen excess conditions and the ChIP-seq data from nitrogen limiting conditions aligned at the third track. Levels of gene expression are indicated in the bottom track. Vertical line through the peak indicates the GlnR binding site. [file 1471-2164-14-301-S6.pptx]

## Slide 1
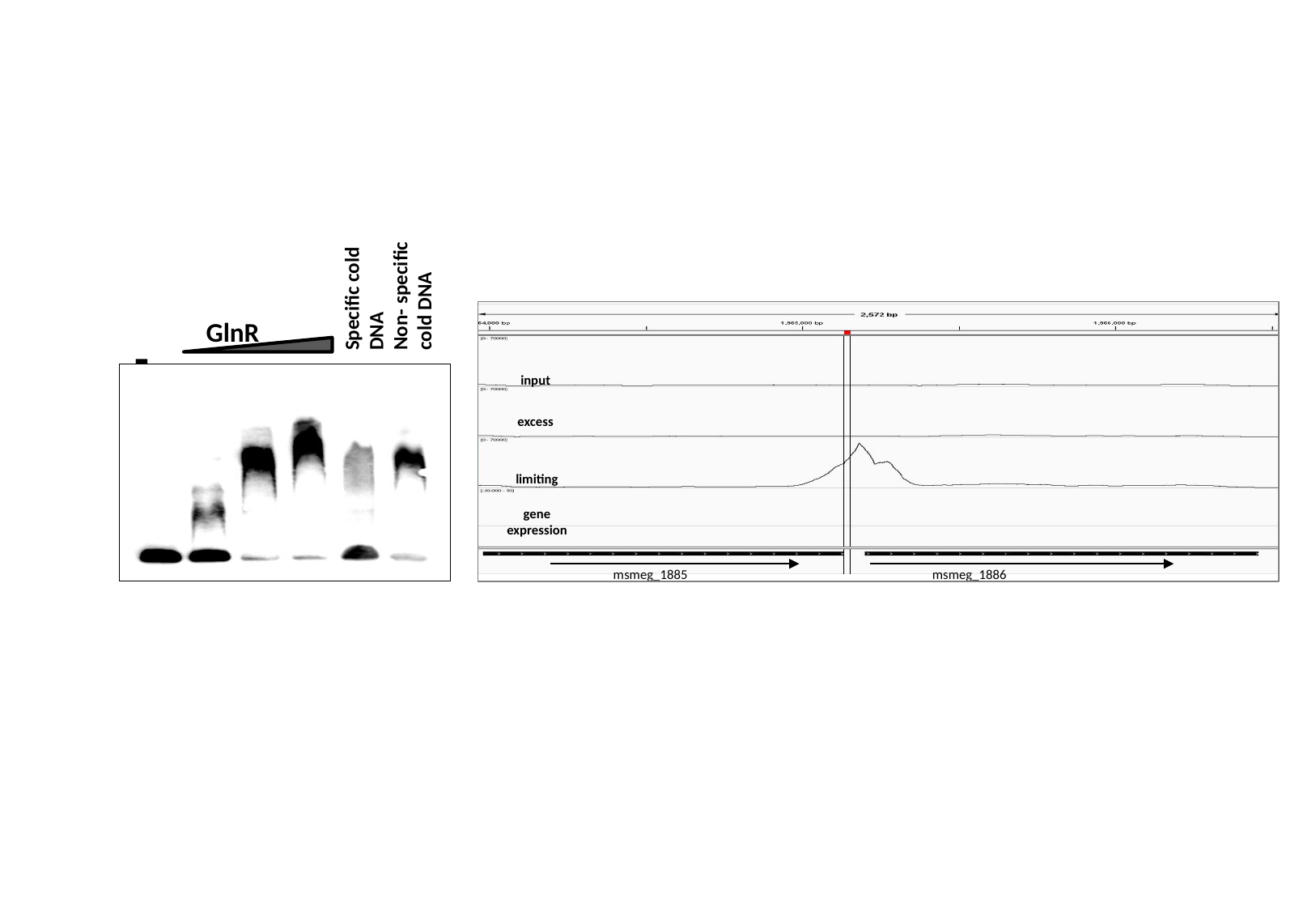

Non- specific cold DNA
Specific cold DNA
GlnR
-
input
excess
limiting
gene
expression
msmeg_1885
msmeg_1886
