# Supplementary material for: Genome wide analysis of the complete GlnR nitrogen-response regulon in Mycobacterium smegmatis
Source: BMC Genomics. 2013 May 4;14:301. doi: 10.1186/1471-2164-14-301 (PMC3662644; doi:10.1186/1471-2164-14-301)
Supplement: Additional file 8: Table S3 — MEME-derived GlnR consensus binding site with corresponding ChIP-seq peak intensity and fold change in gene expression. [file 1471-2164-14-301-S8.doc]

**Table S3. MEME-derived GlnR consensus binding site with corresponding ChIP-seq peak intensity and fold change in gene expression.**

| Strand | *p*-value |  | [Binding Site Sequence](../../../../E:%5Cvjenkins%20(icfs1sancmmiusers)%5CChIP%5Cpeaks.xlsx#RANGE!sites_doc) |  | Peak number | Peak fold change  (N Limiting vs Input) | Fold change in gene expression (WT vs *glnR* ko) |
| --- | --- | --- | --- | --- | --- | --- | --- |
| - | 1.70E-08 | **GCAATCGCGG** | **GGTA AC GCCGTGGAA AC A** | **GAGCCTGCCT** | 2 | 42.9 | 18.30 |
| - | 1.70E-08 | **GCAATGTGCC** | **GGTA AC GGCGCGTTA AC A** | **ATGATGCACG** | 29 | 58.91 | 9.72 |
| - | 3.49E-08 | **TGTCAGCATG** | **GGAT AC ATGGCCGTA AC A** | **CCCCCGAAAC** | 12 | 46.51 | -13.15 |
| + | 1.46E-07 | **TGGGGTCCCA** | **GTCA AC GTCGAGGAA AC A** | **CGTCTGTCAA** | 20 | 18.6 | -4.08 |
| - | 1.75E-07 | **GCCTTACATC** | **GGAA AC CTGTATGTA AC A** | **GACGATCACA** | 31 | 77.17 | 49.19 |
| - | 1.75E-07 | **CGAACGCAAC** | **CGCA AC GTCGTCGCA AC A** | **TGGCGTGGGT** | 42 | 233.93 | 14.88 |
| + | 2.09E-07 | **ATACCGCCCG** | **CGAA AC ACCTCCGAA AC A** | **TCAACGGAGC** | 40 | 34.38 | No DE |
| + | 2.49E-07 | **CTCCCAGCTT** | **CGAA AC ATGTTTGCA AC A** | **AACCCGCATA** | 5 | 6.13 | 22.97 |
| + | 2.49E-07 | **AGCAATGCTC** | **CGCA AC ACTGATGTA AC A** | **ACGGCGCGGC** | 10 | 6.48 | 2.41 |
|  |  |  |  |  | 10 |  | 4.22 |
| + | 2.49E-07 | **AAACCGATTG** | **CGTA AC GTCGGCGCA AC A** | **TCGGGTTGAC** | 34 | 184.71 | 19.96 |
| + | 2.95E-07 | **TTTGTCGGCG** | **GGAA AC ATGAGCGTA AC A** | **GTGATCGGGA** | 19 | 101.46 | 98.84 |
| - | 3.49E-07 | **CGATAACCGG** | **CTTA AC ATTCCGTTA AC A** | **TCGTTGGGGC** | 44 | 18.6 | No DE |
| + | 4.08E-07 | **CCGCGCCTTG** | **GTTA AC AGATAGGTA AC A** | **CATCGAACAT** | 26 | 6.46 | No DE |
| + | 1.00E-06 | **GCTTACCGAC** | **GGCT AC ATGAACGAA AC A** | **TTCGGGTGAC** | 17 | 39.94 | 38.83 |
| - | 1.00E-06 | **CTTGCACCCC** | **CGTA AC ACAGATTTA AC A** | **GCCGGTGCAT** | 35 | 49.84 | 12.61 |
| - | 1.51E-06 | **TACGTCGTTC** | **CGAA AC GTCCAGGAA AT A** | **CTGGCGCCCG** | 7 | 6.47 | -3.80 |
|  |  |  |  |  | 7 |  | 3.35 |
| - | 1.96E-06 | **TTCTAACAGG** | **CGTA AT GGAGCCTTA AC A** | **AAAACGCCGA** | 49 | 9.9 | No DE |
| - | 2.23E-06 | **GACATGTTCT** | **GTAA AC GCTCACGAA AC A** | **TTTGCCGTGT** | 48 | 16.34 | 8.06 |
| + | 3.22E-06 | **TTTCACCTCA** | **GGCA AC ACCTACGAA AC C** | **GTTCATCGCG** | 15 | 9.94 | 120.71 |
| - | 3.63E-06 | **AAACATCAGA** | **TGCA AC AGTGCAGAA AC A** | **TTTGTGTGCA** | 14 | 10.94 | No DE |
| + | 4.08E-06 | **ATCTCACAGC** | **GGCA AT GTGCTCGTA AT A** | **AGTGCAGCAT** | 4 | 27.07 | 263.40 |
| - | 4.58E-06 | **GCGAAACGGC** | **GTTC AC CCGTTCGTA AC A** | **CGATCTACCC** | 25 | 8.66 | No DE |
| - | 6.42E-06 | **ATATTTCGCG** | **CGTT TC GCCGCCGAA AC A** | **TCGCGAACAC** | 36 | 384.39 | 103.26 |
| - | 7.17E-06 | **TTTTCGCCGA** | **GTTC AC AGCGAGATA AC A** | **CGCGTTGGAT** | 23 | 56.3 | 50.82 |
| - | 7.17E-06 | **TGAACAGCGG** | **CGTT AC GGTGTGTTA AC T** | **GCGGGCTAAA** | 46 | 31.59 | 24.75 |
| - | 7.17E-06 | **TCAATCAGCC** | **GGAA AT CGTCCTTTA AC A** | **CGTTTGTGAC** | 28 | 22.78 | 2.12 |
| + | 1.21E-05 | **GATGTTTAGA** | **CTTT AC TGCTTGGTA AC C** | **TACGGAGCCG** | 13 | 19.25 | No DE |
| + | 1.21E-05 | **GGCCCGTTCG** | **GGAC AC CTCGGGTAA AC A** | **CGTATCGCCG** | 38 | 63.56 | 57.30 |
| - | 1.48E-05 | **GAAAAATTTG** | **CGTT AC AAGAAATTA AC A** | **AGCACGATTG** | 16 | 19.49 | -2.07 |
| + | 1.48E-05 | **CGGTTTGAGT** | **TTTA AC CACGCTGCA AC A** | **CTTGGCGACC** | 47 | 23.65 | 255.89 |
| + | 1.81E-05 | **CCGTGGCGTG** | **ATTT AC GGCATGGAA AC A** | **GGCTCTGAAC** | 8 | 71.93 | 277.38 |
| - | 1.81E-05 | **CACGGCCCCG** | **CGTT AC GTTGTGGTG AC C** | **TGACGCAACG** | 22 | 331.2 | 782.41 |
| + | 2.19E-05 | **CGACTGATGA** | **CGTC AC ATTCTTGAA AC T** | **TCACGACAAC** | 27 | 8.19 | 227.95 |
| - | 2.89E-05 | **TGTAGCGAGC** | **GGTA AC AGGAACGTT AC T** | **GTGGCGGCCA** | 50 | 17.72 | 128.25 |
| + | 2.89E-05 | **CCACCCCTGA** | **GGTC AC ATCACTTTA AT C** | **TCGACGCAAT** | 51 | 199.66 | 385.33 |
| - | 3.47E-05 | **TCATGTCGAG** | **GTTA AT TTGTTCGTC AC A** | **CACAGACATT** | 24 | 105.92 | 583.80 |
| + | 3.79E-05 | **CTTCACACAG** | **CGCG AC ATCGCGGCA AT A** | **TCGGGTTCTT** | 1 | 8.41 | 76.38 |
| + | 3.79E-05 | **CTGGCAGTTA** | **GTTG AC ACGCCAGTA AC A** | **ATCGCGGCAT** | 11 | 64.64 | 2.77 |
| + | 3.79E-05 | **TCAGTCTGTG** | **GGTT AC GTTTGCGAA AA A** | **TTTCTGTTGC** | 53 | 10.78 | 5.78 |
| + | 6.86E-05 | **CATCAGGACC** | **GGCC AT CCGGTATTA AC A** | **AGATCTTTTA** | 41 | 57.47 | 27.12 |
| - | 8.73E-05 | **CTGGTGACCG** | **CGTA AC GGCTTTCTC AC A** | **GGTGCCCGCC** | 3 | 8.4 | 24.62 |
| + | 1.02E-04 | **AGCCCCTGCC** | **TTCA AC TGGGTTGCA AC C** | **GTCGAAATGT** | 32 | 7.38 | No DE |
| - | 1.10E-04 | **CTTCGTGTGA** | **GGAT TT GGCGCGGCA AC C** | **GTGCAAGATC** | 6 | 54.34 | 6.31 |
| - | 1.10E-04 | **GGTACGCCTG** | **GGCA AT GGTGCGGCA AC G** | **GGGTTCTCCG** | 45 | 11.62 | 4.09 |
| - | 1.10E-04 | **ACTTGTCACC** | **CGTC AC CGCGACGAC AT A** | **CCGAGTCCGT** | 9 | 19.49 | 10.73 |
|  |  |  |  |  | 9 |  | 3.47 |
| - | 1.10E-04 | **CTTCGTGTGA** | **GGAT TT GGCGCGGCA AC C** | **GTGCAAGATC** | 18 | 67.87 | 10.06 |
| - | 1.59E-04 | **GTCGCCGAAC** | **CGCA AT CTGGGCGCG AC A** | **CGGTCTAGGG** | 43 | 27.24 | 29.14 |
| + | 2.11E-04 | **GCGTGGCGCG** | **TGAC AT GACCCCGTA AC C** | **CGGGCCGTGT** | 21 | 171.12 | 165.90 |
| - | 2.42E-04 | **AACGGACCTT** | **GGTA TC GGCGCGACT AC A** | **GGCCAAACTG** | 30 | 13.19 | 8.33 |
|  |  |  |  |  | 30 |  | 6.49 |
| - | 2.95E-04 | **TTTCCTGACG** | **CTAA CC CCGCGGGAA AC A** | **TCCCGGAACA** | 33 | 7.98 | 115.66 |
| + | 2.95E-04 | **GTCGAGGTCG** | **TGAA AC GGCGTGGAA TT C** | **ATGCTTCGTT** | 37 | 17.09 | 102.03 |
| **-** | **4.05E-04** | **CTCATCGAGT** | **CGTT AT CGGACCGTT AT C** | **TGCCCGGGAT** | **39** | **11.12** | **No DE** |
| **-** | **2.22E-03** | **GCAGCAGTTC** | **CTCA CC GCGGCCTCG AC C** | **GGCGACATGG** | **52** | **12.65** | **No DE** |
