# Supplementary material for: Genome wide analysis of the complete GlnR nitrogen-response regulon in Mycobacterium smegmatis
Source: BMC Genomics. 2013 May 4;14:301. doi: 10.1186/1471-2164-14-301 (PMC3662644; doi:10.1186/1471-2164-14-301)
Supplement: Additional file 9: Table S4 — Primer sequences used in this study. [file 1471-2164-14-301-S9.doc]

**Table S4**. Primer sequences used in this study.

| **Application** | **Name** | **Sequence (5’ – 3’)** |
| --- | --- | --- |
| Cloning *M. smegmatis* *glnR* with N terminal His_6_ tag into pET28b | MS_His-GlnR_F  MS_His-GlnR_R | GACTATCATATGTTGGATCTACTGCTACTG  GACTATCTCGAGTCACTGACTGGTCAACCG |
| Cloning *M. tuberculosis* *glnR* with N terminal His_6_ tag into pET28b | TB_His-GlnR_F  TB_His-GlnR_R | GACTATCATATGTTGTTGGAGTTATTACTG  GACTATCTCGAGTCACTGACTGCGCAACGG |
| Amplification of MS peak 9 for EMSA | Peak 9F  Peak 9R | GAGTGTTTGCGGGGCGTTAC  TTTGTGTGAACCTCCTTGG |
| Amplification of MS peak 17 for EMSA | Peak 17F  Peak 17R | CGTCGATGTGGCGCTGCAC  GCGCTGCTGGTCATGG |
| Amplification of MS peak 21 for EMSA | Peak 21F  Peak 22R | AGCTTGCCTACGAGCTCG  AATGAGGGATGCTGCGAG |
| Amplification of MS peak 22 for EMSA | Peak 22F  Peak 22R | CTACCGGACACACAACG  AACGGTGTGCTTCCTCC |
| Amplification of MS peak 42 for EMSA | Peak 42F  Peak 42R | CATGAGCGCCATCAACTTC  GACGCGTCCATTCGGTTGTC |
| Amplification of MSMEG3224 upstream region for EMSA and rate limiting PCR | 3224 F  3224 R | GCCTGTTGCAGTTGATCG  GTACGGGTCGCGCACCTTGTC |
| Amplification of MS peak 1 for rate limiting PCR | Peak 1F  Peak 1R | TTGTGGCCTGACTGTGGTCC  AGGCTAAGAACCCGATATTG |
| Amplification of MS peak 34 for rate limiting PCR | Peak 34F  Peak 34R | ATAGGCGCGTGCGGATGTC  AACCCGATGTTGCGCCGAC |
| Amplification of MS peak 13 for EMSA and rate limiting PCR | Peak 13F  Peak 13R | AAGCCGGATCCAGACGTG  GCTCGATACCCAGGTTCTC |
| Amplification of MS peak 14 for rate limiting PCR | Peak 14F  Peak 14R | ACTCGACAGGCGATCGGAAG  GAAACAGCGTTTCTTAC |
| Amplification of MS peak 26 for rate limiting PCR | Peak 26F  Peak 26R | GTCACGGCAAGGGTGGAC  GTTGTGACCGGACACAC |
| Amplification of MS peak 32 for rate limiting PC | Peak 32F  Peak 32R | CGACAAGAGAAATGGCCGAG  AAGGCAAGAGTGCGAATGAC |
| Amplification of MS peak 39 for rate limiting PCR | Peak 39F  Peak 39R | AGATAACGGTCCGATAAC  TTGCCGTCTACCTGCATG |
| Amplification of MS peak 40 for rate limiting PCR | Peak 40F  Peak 40R | CAACAAACCCCGTGGTCAG  GAATTTATCGTTTCGAG |
| Amplification of MS peak 44 for rate limiting PCR | Peak 44F  Peak 44R | GATGTCTCGGCATCGAGCAAC  CGATAACCGGTGTCGATC |
| Amplification of MS peak 49 for rate limiting PCR | Peak 49F  Peak 49R | TCAGTGCTACCTCCAAG  CGACGACCTCTACACC |
| WT oligonucleotides for MS binding site analysis of peak 2 EMSA | Peak 2WT F  Peak 2WT R | GCAATCGCGGGGTAACGCCGTGGAAACAGAGCCTGCCT  AGGCAGGCTCTGTTTCCACGGCGTTACCCCGCGATTGC |
| AC to GG mutation for MS binding site analysis of peak 2 EMSA | Peak 2GG F  Peak 2GG F | GCAATCGCGGGGTA**GG**GCCGTGGAA**GG**AGAGCCTGCCT  AGGCAGGCTCT**CC**TTCCACGGC**CC**TACCCCGCGATTGC |
| A to G mutation for MS binding site analysis of peak 2 EMSA | Peak 2G F  Peak 2G R | GCAATCGCGGGGTA**G**CGCCGTGGAA**G**CAGAGCCTGCCT  AGGCAGGCTCTG**C**TTCCACGGCG**C**TACCCCGCGATTGC |
| WT oligonucleotides for MS binding site analysis of peak 24 EMSA | Peak 24WT F  Peak 24WT R | TCATGTCGAGGTTAATTTGTTCGTCACACACAGACATT  AATGTCTGTGTGTGACGAACAAATTAACCTCGACATGA |
| AT to GG mutation for MS binding site analysis of peak 24 EMSA | Peak 24GG F  Peak 24GG R | TCATGTCGAGGTTA**GG**TTGTTCGTC**GG**ACACAGACATT  AATGTCTGTGT**CC**GACGAACAA**CC**TAACCTCGACATGA |
| A to G mutation for MS binding site analysis of peak 24 EMSA | Peak 24G F  Peak 24G R | TCATGTCGAGGTTA**G**TTTGTTCGTC**G**CACACAGACATT  AATGTCTGTGTG**C**GACGAACAAA**C**TAACCTCGACATGA |
| Oligonucleotides increasing ACn9AC distance to ACn12AC | Peak 2long F  Peak 2long R | GCAATCGCGGGGTAAC**GCCGTCCCGGAA**ACAGAGCCTGCCT  AGGCAGGCTCTGT**TTCCGGGACGGC**GTTACCCCGCGATTGC |
| Oligonucleotides decreasing ACn9AC distance to ACn6AC | Peak 2short F  Peak 2short R | GCAATCGCGGGGTAAC**GTGGAA**ACAGAGCCTGCCT  AGGCAGGCTCTGT**TTCCAC**GTTACCCCGCGATTGC |
