# Supplementary material for: Genome wide analysis of the complete GlnR nitrogen-response regulon in Mycobacterium smegmatis
Source: BMC Genomics. 2013 May 4;14:301. doi: 10.1186/1471-2164-14-301 (PMC3662644; doi:10.1186/1471-2164-14-301)
Supplement: Additional file 10: Table S5 — Custom Taqman M. smegmatis gene expression primer and probe sequences used in this study. [file 1471-2164-14-301-S10.doc]

**Table S5. Custom Taqman *M. smegmatis*** gene expression primer and probe sequences used in this study.

| **Gene** | **Forward primer (5’-3’)** | **Reverse primer (5’-3’)** | **FAM Reporter probe (5’-3’)** |
| --- | --- | --- | --- |
| MSMEG2332 | CGTTCGGCGCGATCATC | CCGGCCTCGTTGGAGTAG | ACGCCCCACATCACG |
| MSMEG6697 | TGGACGTTGTTGCCATGGA | GTGGCGGCGGTCTTG | AACCCGGAGAACCC |
| MSMEG2758 (*sigA*) | CGAGAAGGGCGAGAAGCT | CGCCTCCAGCAGATGGTTTT | CAGCGCCGCGACATG |
